# Efficacy and Safety of Cilostazol in Mild Cognitive Impairment: A Randomized Clinical Trial

**DOI:** 10.1001/jamanetworkopen.2023.44938

**Published:** 2023-12-04

**Authors:** Satoshi Saito, Keisuke Suzuki, Ryo Ohtani, Takakuni Maki, Hisatomo Kowa, Hisatsugu Tachibana, Kazuo Washida, Nobuya Kawabata, Toshiki Mizuno, Rie Kanki, Shinji Sudoh, Hiroshi Kitaguchi, Katsuro Shindo, Akihiro Shindo, Nobuyuki Oka, Keiichi Yamamoto, Fumihiko Yasuno, Chikage Kakuta, Ryosuke Kakuta, Yumi Yamamoto, Yorito Hattori, Yukako Takahashi, Yuriko Nakaoku, Shuichi Tonomura, Naoya Oishi, Toshihiko Aso, Akihiko Taguchi, Tatsuo Kagimura, Shinsuke Kojima, Masanori Taketsuna, Hidekazu Tomimoto, Ryosuke Takahashi, Hidenao Fukuyama, Kazuyuki Nagatsuka, Haruko Yamamoto, Masanori Fukushima, Masafumi Ihara

**Affiliations:** 1 Department of Neurology, National Cerebral and Cardiovascular Center, Suita, Japan; 2 Innovation Center for Translational Research, National Center for Geriatrics and Gerontology, Obu, Japan; 3 Department of Neurology, National Hospital Organization Kyoto Medical Center, Kyoto, Japan; 4 Department of Neurology, Graduate School of Medicine, Kyoto University, Kyoto, Japan; 5 Division of Neurology, Kobe University Hospital, Kobe, Japan; 6 Division of Neurology, Yachiyo Hospital, Anjo, Japan; 7 Department of Neurology, Kyoto Prefectural University of Medicine, Kyoto, Japan; 8 Department of Neurology, Osaka City General Hospital, Osaka, Japan; 9 Department of Neurology, National Hospital Organization, Utano National Hospital, Kyoto, Japan; 10 Department of Neurology, Kurashiki Central Hospital, Kurashiki, Japan; 11 Department of Neurology, Graduate School of Medicine, Mie University, Tsu, Japan; 12 Department of Neurology, National Hospital Organization Minami Kyoto Hospital, Joyo, Japan; 13 Internal Medicine and Neurology, Nara Midori Clinic, Nara, Japan; 14 Department of Psychiatry, National Center for Geriatrics and Gerontology, Obu, Japan; 15 Department of Data Science, National Cerebral and Cardiovascular Center, Suita, Japan; 16 Department of Molecular Innovation in Lipidemiology, National Cerebral and Cardiovascular Center, Suita, Japan; 17 Department of Psychiatry, Graduate School of Medicine, Kyoto University, Kyoto, Japan; 18 Laboratory for Brain Connectomics Imaging, RIKEN Center for Biosystems Dynamics Research, Kobe, Japan; 19 Department of Regenerative Medicine Research, Institute of Biomedical Research and Innovation, Kobe, Japan; 20 Translational Research Center for Medical Innovation, Foundation for Biomedical Research and Innovation at Kobe, Kobe, Japan; 21 Research and Educational Unit of Leaders for Integrated Medical System, Kyoto University, Kyoto, Japan; 22 Foundation of Learning Health Society Institute, Nagoya, Japan

## Abstract

**Importance:**

Recent evidence indicates the efficacy of β-amyloid immunotherapy for the treatment of Alzheimer disease, highlighting the need to promote β-amyloid removal from the brain. Cilostazol, a selective type 3 phosphodiesterase inhibitor, promotes such clearance by facilitating intramural periarterial drainage.

**Objective:**

To determine the safety and efficacy of cilostazol in mild cognitive impairment.

**Design, Setting, and Participants:**

The COMCID trial (A Trial of Cilostazol for Prevention of Conversion from Mild Cognitive Impairment to Dementia) was an investigator-initiated, double-blind, phase 2 randomized clinical trial. Adult participants were registered between May 25, 2015, and March 31, 2018, and received placebo or cilostazol for up to 96 weeks. Participants were treated in the National Cerebral and Cardiovascular Center and 14 other regional core hospitals in Japan. Patients with mild cognitive impairment with Mini-Mental State Examination (MMSE) scores of 22 to 28 points (on a scale of 0 to 30, with lower scores indicating greater cognitive impairment) and Clinical Dementia Rating scores of 0.5 points (on a scale of 0, 0.5, 1, 2, and 3, with higher scores indicating more severe dementia) were enrolled. The data were analyzed from May 1, 2020, to December 1, 2020.

**Interventions:**

The participants were treated with placebo, 1 tablet twice daily, or cilostazol, 50 mg twice daily, for up to 96 weeks.

**Main Outcomes and Measures:**

The primary end point was the change in the total MMSE score from baseline to the final observation. Safety analyses included all adverse events.

**Results:**

The full analysis set included 159 patients (66 [41.5%] male; mean [SD] age, 75.6 [5.2] years) who received placebo or cilostazol at least once. There was no statistically significant difference between the placebo and cilostazol groups for the primary outcome. The least-squares mean (SE) changes in the MMSE scores among patients receiving placebo were –0.1 (0.3) at the 24-week visit, –0.8 (0.3) at 48 weeks, –1.2 (0.4) at 72 weeks, and –1.3 (0.4) at 96 weeks. Among those receiving cilostazol, the least-squares mean (SE) changes in MMSE scores were –0.6 (0.3) at 24 weeks, –1.0 (0.3) at 48 weeks, –1.1 (0.4) at 72 weeks, and –1.8 (0.4) at 96 weeks. Two patients (2.5%) in the placebo group and 3 patients (3.8%) in the cilostazol group withdrew owing to adverse effects. There was 1 case of subdural hematoma in the cilostazol group, which may have been related to the cilostazol treatment; the patient was successfully treated surgically.

**Conclusions and Relevance:**

In this randomized clinical trial, cilostazol was well tolerated, although it did not prevent cognitive decline. The efficacy of cilostazol should be tested in future trials.

**Trial Registration:**

ClinicalTrials.gov Identifier: NCT02491268

## Key Points

**Question:**

Is cilostazol, a selective type 3 phosphodiesterase inhibitor, safe and effective for cognitive function among individuals with mild cognitive impairment?

**Findings:**

In this phase 2 randomized clinical trial among 159 adult patients with mild cognitive impairment, cilostazol exhibited good tolerability. However, it did not yield improvements in the primary outcome measure, the Mini-Mental State Examination score.

**Meaning:**

These results suggest that, although cilostazol is well tolerated, further research is needed to establish its efficacy.

## Teaser

This randomized clinical trial investigates whether cilostazol is safe and effective for cognitive function among individuals with mild cognitive impairment.

## Introduction

Cerebrovascular dysfunction significantly contributes to the pathogenesis of Alzheimer disease (AD). Epidemiological investigations have emphasized the effectiveness of strict control of vascular risk factors as a preventive strategy for dementia, highlighting the strong association between AD and cerebrovascular disease (CVD).^1,2^ One of the primary factors associated with AD is an imbalance between the production and clearance of amyloid-β (Aβ).^3,4,5^ In contrast, CVD is associated with both increased Aβ production and impaired elimination.^5,6,7,8,9,10^ Reduced cerebral blood flow is believed to influence enzymes involved in amyloid precursor protein cleavage, leading to increased Aβ production.^7,8^ A significant portion of Aβ clearance occurs through vascular-mediated systems, including active transport across the blood-brain barrier(BBB), glymphatic clearance, and intramural periarterial drainage, all of which can be disrupted by CVD.^5,6,9,10^

Emerging evidence supporting the effectiveness of Aβ immunotherapy^11,12^ underscores the need to investigate novel therapies that enhance the Aβ clearance.^4,13^ Interstitial fluids, including antibody-solubilized Aβ derived from the parenchymal plaque, enter the intramural periarterial drainage pathways within the basement membranes of capillaries and are drained through the basement membranes of surrounding vascular smooth muscle cells and, finally, through cervical lymph nodes.^14,15^ The disassembly of Aβ plaques achieved by the anti-Aβ antibody is associated with a paradoxical increase in cerebral amyloid angiopathy (CAA), owing to the mobilization of excessive Aβ.^16,17^ A major factor associated with sporadic CAA is intramural periarterial drainage dysfunction, which may further result in Aβ elimination failure.^14,18^ Sporadic AD is mainly associated with decreased Aβ clearance.^3,4^ The high comorbidity of AD and CAA suggests that promoting Aβ clearance through the intramural periarterial drainage should be explored as a therapeutic approach for dementia.^19,20^

In previous studies by some of us,^21,22^ it was previously reported that cilostazol, a selective type 3 phosphodiesterase inhibitor, was associated with Aβ clearance through intramural periarterial drainage, ameliorating Aβ deposition and cognitive impairments in *APP* transgenic mice. Cilostazol has been approved by the US Food and Drug Administration for treating intermittent claudication, and it is also used in Asia for the secondary prevention of ischemic stroke.^19^ Vascular Aβ has been associated with impaired vasodilation,^23^ whereas cilostazol has been associated with exerting vasodilatory effects by increasing cyclic adenosine monophosphate levels in vascular smooth muscle cells.^24^ Furthermore, cilostazol may alleviate Aβ production and suppress tau phosphorylation by inhibiting glycogen synthase kinase 3β.^25,26^ These findings suggest that cilostazol may be effective for treating patients with dementia. Several studies have examined the beneficial effects of cilostazol on cognitive impairment, but the results are inconsistent.^27,28,29,30,31,32^ To our knowledge, clinical trials specifically targeting patients with neurocognitive disorders who do not have a history of stroke have yet to be conducted. We therefore conducted a randomized clinical trial to assess the safety and efficacy of cilostazol in patients with mild cognitive impairment (MCI).

## Methods

### Trial Design

A Trial of Cilostazol for Prevention of Conversion from MCI to Dementia (COMCID) was a phase 2, investigator-initiated, double-blind randomized clinical trial, approved by the Institutional Review Board of the National Cerebral and Cardiovascular Center, Suita, Japan, and 14 other participating hospitals. Written informed consent was obtained from all patients. We followed the Consolidated Standards of Reporting Trials (CONSORT) reporting guideline. The trial protocol has been previously published^33^ and is provided in Supplement 1.

### Participants

Patients with MCI were screened according to the core clinical criteria of the National Institute on Aging and the Alzheimer’s Association.^34^ Mini-Mental State Examination (MMSE) scores of 22 to 28 points (inclusive; scores range from 0 to 30, with lower scores indicating greater cognitive impairment, and Clinical Dementia Rating (CDR) scores of 0.5 points (on a scale of 0, 0.5, 1, 2, and 3, with higher scores indicating more severe dementia), combined with other eligibility criteria (eTable 1 in Supplement 2), were required for registration. The enrolled participants were randomly assigned to the cilostazol or placebo groups (eMethods in Supplement 2).

### Interventions

Between May 25, 2015, and March 31, 2018, participants were treated for up to 96 weeks with 1 placebo tablet twice daily or 1 cilostazol tablet, 50 mg twice daily; in Japan, the approved cilostazol dosage for peripheral arterial disease and ischemic stroke is 200 mg daily. Dose reduction during the treatment protocol was not allowed. Cilostazol and the placebo were provided by Otsuka Pharmaceutical Co, Ltd.

The intervention ceased when patients were diagnosed with dementia because the secondary end points included the conversion to dementia. In Japan, cholinesterase inhibitors and memantine have been widely administered to patients with dementia but not approved for those with MCI. To minimize the effects of these antidementia drugs on evaluation of cognitive function after conversion to dementia, conversion was included as a criterion for discontinuation.

### End Points

The primary end point was the change in the MMSE score^35,36^ from baseline to final observation. The secondary end points included time to conversion to dementia and changes in the CDR Sum of Box (CDR-SB) scores^37,38^; the 14-item Alzheimer’s Disease Assessment Scale-Cognitive Subscale^39^; Wechsler Memory Scale-Revised, Logical Memory II^40^; and Alzheimer’s Disease Cooperative Study-Activities of Daily Living for Mild Cognitive Impairment.^41^ Hippocampal volume was evaluated using magnetic resonance imaging. In addition to the Trail Making Test, Part B^42^ and the Free and Cued Selective Reminding Test,^43^ the blood levels of Aβ-albumin complexes were assessed as exploratory end points.^44^ The schedule of the efficacy assessments is described in eFigure 1 in Supplement 2, and the details of the psychological examinations are provided in the eMethods in Supplement 2.

### Safety Profile

Safety analyses included all adverse events, which were categorized into 3 classifications: seriousness, severity, and causal relationship. The details are described in the eMethods in Supplement 2. Clinical laboratory tests, including complete blood cell count, glucose and lipid level, electrolyte concentration, and liver and renal function measurements, were performed at baseline and the 4-week visit.

### Hippocampal Volumetry

Hippocampal volumetry was conducted at baseline, at the 96-week visit, or at early termination.^45,46^ The details are described in the eMethods in Supplement 2.

### Amyloid β-Albumin Complex Blood Levels

The blood levels of the Aβ-albumin complex were measured at baseline and at the 24-week visit by a sandwich ELISA.^44^ The details are described in the eMethods in Supplement 2.

### Statistical Analyses

Data were analyzed from May 1, 2020, to December 1, 2020. All analyses were performed as specified in the trial protocol and statistical analysis plan (Supplement 1). Prior to trial initiation, we estimated that more than 80 patients per group were required to show a statistically significant efficacy of cilostazol as the primary end point.^33^ The full analysis set was used for efficacy analyses. However, the results after discontinuation of the treatment protocol due to dementia conversion were not included in the analyses because antidementia drugs had been widely administered to patients with dementia, which could have improved the psychological examination scores. Changes in the psychological examination scores over the trial course were analyzed using a linear mixed-effects model for repeated measures that included the treatments, time points, sex, and interaction of treatments with time points as fixed effects; patients as a random effect; and baseline scores as a covariate. The time to convert to all-cause dementia was analyzed using the Kaplan-Meier and log-rank tests. We analyzed the yearly loss of hippocampal volume and changes in Aβ-albumin complex blood levels from baseline to the 24-week visit using analysis of covariance (ANCOVA), with baseline values as a covariate. The interaction of the treatments with the baseline values of hippocampal volume and Aβ-albumin complex blood levels was also evaluated. The level of significance was set as 2-sided *P* < .05.

## Results

### Patients

**Figure 1.**
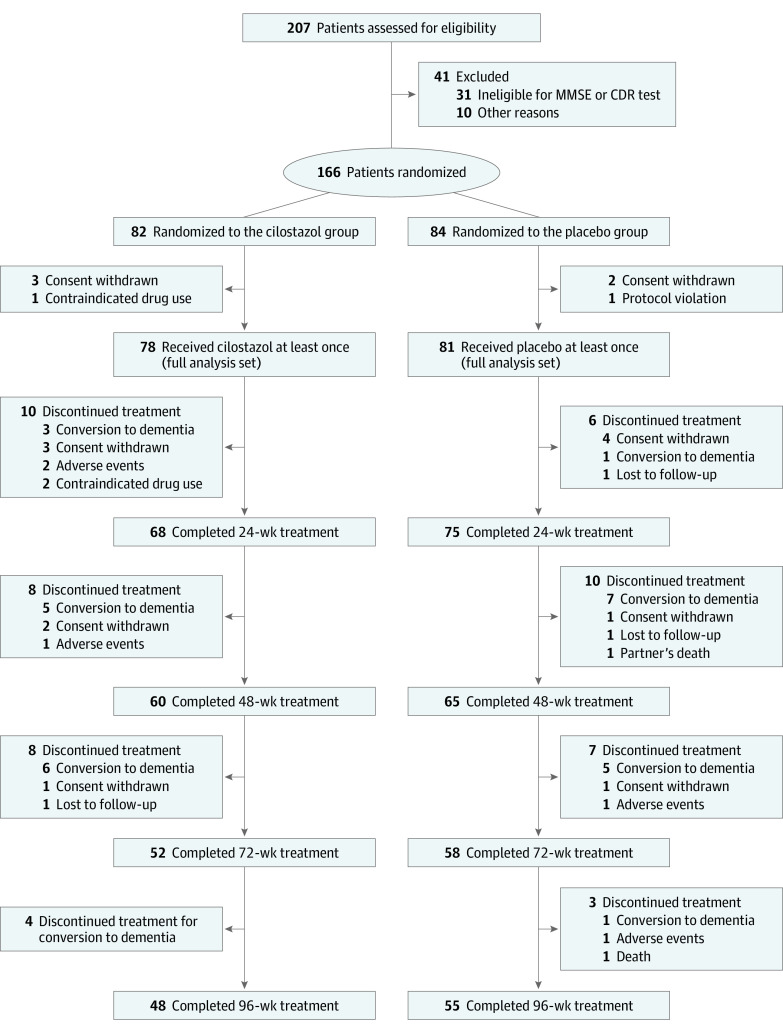
CONSORT Diagram Patients who received cilostazol or placebo at least once comprised the full analysis set (N = 159). CDR indicates Clinical Dementia Rating; CONSORT, Consolidated Standards of Reporting Trials; MMSE, Mini-Mental State Examination.

Between May 2015 and March 2018, 207 patients were screened for study entry and provisionally registered. After a central review of the MMSE and CDR results, 166 patients (mean [SD] age, 75.6 [5.2] years) were enrolled and randomly allocated to the cilostazol or placebo group (Figure 1). Because 4 patients in the cilostazol group and 3 patients in the placebo group were withdrawn before initiating the treatment protocol, the full analysis set included 159 patients who received cilostazol (n = 78) or placebo (n = 81) at least once (93 [58.5%] female and 66 [41.5%] male; mean [SD] age, 75.6 [5.2] years). The mean (SD) MMSE score was 25.5 (1.9), and the mean (SD) CDR-SB score was 2.6 (1.0). The demographic characteristics are summarized in Table 1. Antithrombotic agents were administered to 13 patients (16.7%) in the cilostazol group and to 15 (18.5%) in the placebo group.

**Table 1.** Clinical Characteristics

Characteristic	Participants, No. (%)	
	Placebo (n = 81)	Cilostazol (n = 78)
Age, mean (SD), y	75.4 (5.6)	75.7 (4.8)
Sex		
Female	47 (58.0)	46 (58.9)
Male	34 (42.0)	32 (41.0)
Educational level, y		
≤7	1 (1.2)	0
8-12	50 (61.7)	50 (64.1)
13-15	12 (14.8)	13 (16.7)
≥16	18 (22.2)	15 (19.2)
Familial history of AD	15 (18.5)	16 (20.5)
Consanguineous parents	0	1 (1.3)
Current smoker	4 (4.9)	3 (3.8)
Alcohol consumer	38 (46.9)	28 (35.9)
Body mass index, mean (SD)^a^	22.6 (4.1)	22.2 (3.2)
Medical history		
Hypertension	42 (51.9)	36 (46.2)
Dyslipidemia	46 (56.8)	41 (52.6)
Diabetes	11 (13.6)	11 (14.1)
Stroke	10 (12.3)	7 (9.0)
General anesthesia	36 (44.4)	28 (35.9)
Head trauma	6 (7.4)	3 (3.8)
Baseline psychological test score, mean (SD)		
MMSE^b^	25.5 (2.0)	25.6 (1.8)
CDR-SB^c^	2.6 (1.0)	2.5 (0.9)
ADAS-Cog 14^d^	25.2 (6.7)	25.9 (7.1)
WMS-R Logical Memory II^e^	2.8 (2.8)	3.1 (3.1)
ADCS-ADL-MCI^f^	41.2 (5.8)	41.3 (5.3)
Hippocampal volume, mean (SD), mL	6.19 (1.15)	6.01 (0.93)
Blood Aβ-albumin complex level, mean (SD), mg/mL	11.22 (5.93)	10.31 (4.50)
Blood albumin level, mean (SD), g/dL	4.13 (0.27)	4.20 (0.24)

Abbreviations: Aβ, amyloid β; AD, Alzheimer disease; ADAS-Cog 14, 14-item Alzheimer’s Disease Assessment Scale-Cognitive Subscale; ADCS-ADL-MCI, Alzheimer’s Disease Cooperative Study-Activities of Daily Living for Mild Cognitive Impairment; CDR-SB, Clinical Dementia Rating Sum of Box; MMSE, Mini-Mental State Examination; WMS-R, Wechsler Memory Scale-Revised.

SI conversion factor: To convert blood albumin level to grams per liter, multiply by 10.

^a^ Calculated as weight in kilograms divided by height in meters squared.

^b^ Scores range from 0 to 30, with lower scores indicating greater cognitive impairment.

^c^ Scores range from 0 to 18, with higher scores indicating severe dementia.

^d^ Scores range from 0 to 90, with higher scores indicating worse cognitive function.

^e^ Scores range from 0 to 25, with lower scores indicating worse cognitive function.

^f^ Scores range from 0 to 53, with lower scores indicating severe activities of daily living impairment.

Among patients who received the treatment protocol until the 96-week visit without early termination, 48 (61.5%) were in the cilostazol group and 55 (67.9%) were in the placebo group. Among 103 patients who completed the 96-week treatment protocol, 61 (59.2%) were female and 42 (40.8%) were male. The mean (SD) age was 75.6 (5.0) years, the mean (SD) baseline MMSE score was 25.9 (1.8), and the mean (SD) CDR-SB score was 2.5 (0.9). Among 56 patients with early termination, 32 (57.1%) were female and 24 (42.9%) were male. The mean (SD) age was 75.6 (5.6) years, the mean (SD) baseline MMSE score was 25.0 (1.9), and the mean (SD) CDR-SB score was 2.8 (1.0). After early termination, 8 patients in the cilostazol group and 5 in the placebo group were followed up until the 96-week visit. Of the 159 patients who had initiated the treatment protocol in this trial, 116 (73.0%) attended their final 96-week visit. The drug-adherence rates are presented in eTable 2 in Supplement 2.

### Safety

Adverse events were observed in 58 patients (71.6%) in the placebo group and 58 (74.4%) in the cilostazol group (*P* = .72) (Table 2). In the placebo group, 1 patient died due to lung cancer. Two patients (2.5%) in the placebo group and 3 (3.8%) in the cilostazol group withdrew because of adverse events. There were no serious adverse events related to the treatment protocol (ie, no causal relationship noted as yes); however, there were 5 serious adverse events possibly related to the treatment protocol (ie, causal relationship noted as possible), including 1 case of ureterolithiasis and 1 case of atrial fibrillation with heart failure in the placebo group and 2 cases of colon polyps and 1 case of subdural hematoma in the cilostazol group. The patient presenting with a subdural hematoma was a 77-year-old male with mild weakness in the left upper limb. A head computed tomographic image showed a chronic subdural hematoma in the right hemisphere without midline shift. After discontinuing the COMCID trial, the patient underwent a craniotomy with hematoma evacuation, and he fully recovered. The 2 cases of colon polyps were classified as serious adverse events because the patients were admitted for receiving endoscopic surgery. There were no complications during hospitalization, and the treatment protocol was continued. Six patients treated with cilostazol and 2 with placebo showed mild adverse events classified as cardiovascular disease. Clinical laboratory tests showed no significant changes from baseline to the 4-week visit.

**Table 2.** Incidence of Adverse Events

Adverse event	Participants, No. (%)	
	Placebo (n = 81)	Cilostazol (n = 78)
Overall adverse event		
Any	58 (71.6)	58 (74.4)
Discontinued because of adverse event	2 (2.5)	3 (3.8)
Severe	3 (3.7)	3 (3.8)
Serious	15 (18.5)	15 (19.2)
Adverse event in either treatment group^a^		
Nasopharyngitis	21 (25.9)	21 (26.9)
Headache	4 (4.9)	7 (9.0)
Contusion	3 (3.7)	5 (6.4)
Influenza	2 (2.5)	5 (6.4)
Colorectal polyp	2 (2.5)	4 (5.1)
Diarrhea	4 (4.9)	3 (3.8)
Dizziness	2 (2.5)	3 (3.8)
Pruritus	2 (2.5)	3 (3.8)
Spinal compression fracture	1 (1.2)	3 (3.8)
Palpitation	1 (1.2)	3 (3.8)
Eczema	1 (1.2)	3 (3.8)
Fatigue	0	3 (3.8)
Back pain	3 (3.7)	2 (2.6)
Osteoarthritis	3 (3.7)	2 (2.6)
Insomnia	4 (4.9)	1 (1.3)
Dyspnea	4 (4.9)	1 (1.3)
Constipation	3 (3.7)	1 (1.3)
Chest discomfort	3 (3.7)	0

^a^Adverse events were reported in 3% or more of patients in either treatment group.

### Clinical Efficacy

**Figure 2.**
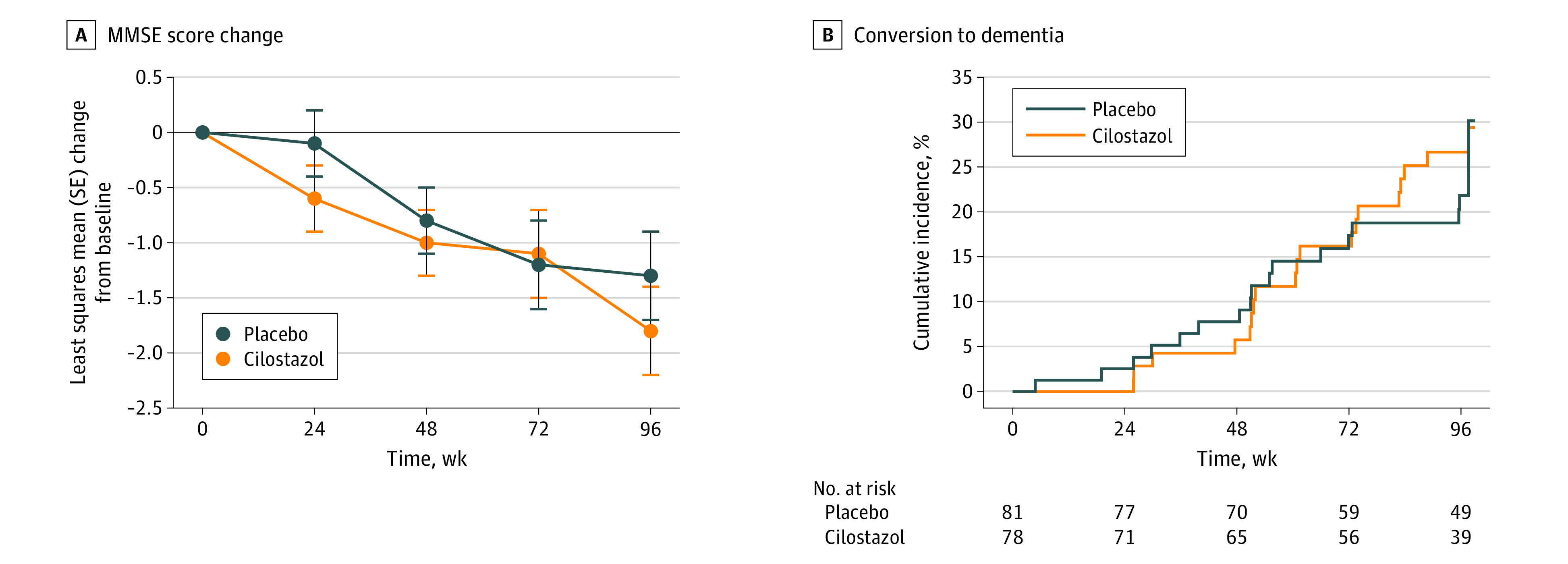
Effects of Cilostazol on Cognitive Decline A, A mixed-effects model with repeated measures was applied by considering treatments, time points, sex, and interaction of treatments and time points as fixed effects; patients as a random effect; and baseline scores as a covariate. Error bars represent SEs. B, A Kaplan-Meier plot shows the cumulative incidence of conversion to all-cause dementia (hazard ratio [cilostazol vs placebo], 1.12; 95% CI, 0.60-2.09; *P* = .71). The log-rank test was performed. MMSE indicates Mini-Mental State Examination.

Cilostazol did not significantly improve the primary end point (Figure 2A). The least-squares mean (SE) changes in the MMSE scores among patients receiving placebo were –0.1 (0.3) at the 24-week visit, –0.8 (0.3) at 48 weeks, –1.2 (0.4) at 72 weeks, and –1.3 (0.4) at 96 weeks. Among those receiving cilostazol, the least-squares mean (SE) changes in MMSE scores were –0.6 (0.3) at 24 weeks, –1.0 (0.3) at 48 weeks, –1.1 (0.4) at 72 weeks, and –1.8 (0.4) at 96 weeks. Results analyzed based on age group, sex, and educational level are summarized in eTable 3 in Supplement 2. Among the 159 patients (78 in the cilostazol group and 81 in the placebo group) in the full analysis set, conversion to all-cause dementia was observed in 20 (25.6%) in the cilostazol group and 20 (24.7%) in the placebo group (hazard ratio, 1.12; 95% CI, 0.60-2.09; *P* = .71) (Figure 2B). Conversion to Alzheimer-type dementia was observed in 20 patients (25.6%) in the cilostazol group and 19 (23.5%) in the placebo group (hazard ratio, 1.18; 95% CI, 0.63-2.22; *P* = .60). Cilostazol did not improve the scores for the CDR-SB (eFigure 2A in Supplement 2); 14-item Alzheimer’s Disease Assessment Scale-Cognitive Subscale (eFigure 2B in Supplement 2); Alzheimer’s Disease Cooperative Study-Activities of Daily Living for Mild Cognitive Impairment (eFigure 2C in Supplement 2); Wechsler Memory Scale-Revised, Logical Memory II (eFigure 2D in Supplement 2); Trail Making Test, Part B (eFigure 2E in Supplement 2); and Free and Cued Selective Reminding Test (eFigure 2F in Supplement 2).

### Biomarkers

**Figure 3.**
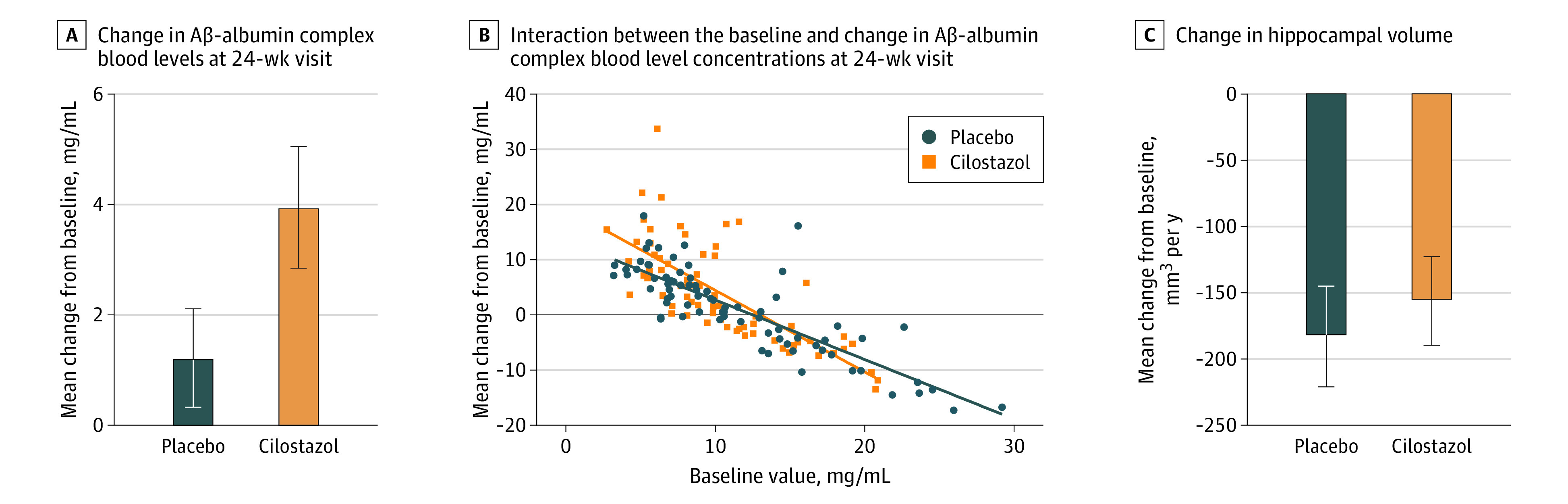
Effects of Cilostazol on Biomarkers A, Error bars represent SEs. B, The blue and orange lines represent the regression lines between the baseline Aβ-albumin complex levels and the changes in the level of Aβ-albumin complex in the placebo and cilostzol group, respectively. C, The hippocampal volume after the protocol treatment was measured at the 96-week visit (n = 35 in the placebo group; n = 30 in the cilostazol group) or early termination (n = 9 in the placebo group; n = 14 in the cilostazol group). Error bars represent SEs.

The mean (SE) incremental changes in Aβ-albumin complex blood levels’ concentration were 1.22 (0.89) mg/mL in the placebo group (n = 76) and 3.95 (1.10) mg/mL in the cilostazol group (n = 67) (Figure 3A). In the ANCOVA for change in the Aβ-albumin complex blood levels, a significant interaction was found between the treatment group and placebo group in the baseline value of the Aβ-albumin complex blood level (Figure 3B). The cilostazol group showed a significant negative correlation of the Aβ-albumin complex blood levels’ concentration between baseline and the 24-week visit (Pearson correlation coefficient, −0.34; *P* = .005), but the placebo group did not (Pearson correlation coefficient, −0.11; *P* = .34). To assess annual changes in hippocampal volumes, magnetic resonance imaging was performed in 44 patients treated with placebo and in 44 patients treated with cilostazol. The mean (SE) annual changes in hippocampal volumes were –182.8 (38.0) mm^3^ in the placebo group and –156.1 (33.4) mm^3^ in the cilostazol group (Figure 3C). In the ANCOVA for annual changes in hippocampal volumes, the interaction was significant between the treatment group and the baseline value of the hippocampal volume.

## Discussion

The COMCID phase 2 randomized clinical trial found that cilostazol was safe but did not improve cognition. A dose-dependent decrease in the incidence of dementia due to cilostazol treatment was reported in a nationwide cohort study in Taiwan^29^; however, we chose to administer a dosage of 100 mg per day (50 mg twice daily). This decision was based on previous retrospective studies by some of us,^27,28^ showing no significant difference in the yearly changes in MMSE scores between patients receiving cilostazol, 100 mg per day, and those receiving it at 200 mg per day, the approved cilostazol dosage in Japan for peripheral arterial disease and ischemic stroke. However, 1 meta-analysis study found fewer adverse events in patients treated with cilostazol, 100 mg per day, compared with those receiving it at 200 mg per day.^47^ In alignment with these observations, the current trial documented only 1 case of severe bleeding, specifically a subdural hemorrhage; the patient underwent a successful surgical procedure. Furthermore, the withdrawal rates due to adverse events were low, with only 2 patients (2.5%) discontinuing treatment in the placebo group and 3 patients (3.8%) in the cilostazol group. These results demonstrated the safety profile of cilostazol treatment, 100 mg per day, for patients with MCI. A second Cilostazol Stroke Prevention Study^48^ involving patients with cerebral infarction showed that hemorrhagic events occurred less frequently in patients with cilostazol than those with aspirin. This reduced incidence of major bleeding may be attributable to its protective effects on vascular endothelial cells.^49^ It is important to note that no serious adverse cardiovascular events were documented in patients receiving cilostazol in the COMCID study. However, it is noteworthy that cilostazol should be used with caution in patients with heart failure, as indicated by a US Food and Drug Administration black box warning.

Regrettably, cilostazol did not yield significant improvements in cognitive function. This outcome may be attributable to the possibility that the participants in the present study were in advanced stages of MCI, particularly late MCI. This becomes particularly apparent when comparing the baseline characteristics of the patients with MCI in our study (mean age, 75.6 [5.2] years; mean [SD] baseline MMSE scores, 25.5 [1.9] and mean [SD] baseline CDR-SB scores, 2.6 [1.0]) with those enrolled in the Japanese Alzheimer’s Disease Neuroimaging Initiative study, one of Japan’s largest cohort studies, who were younger and had scores indicating milder cognitive impairment (mean [SD] age, 72.5 [5.8] years; mean [SD] baseline MMSE scores, 26.6 [1.8] and mean [SD] baseline CDR-SB scores, 1.6 [0.9]).^50^ Earlier initiation of cilostazol treatment, from early or preclinical stages of MCI, might have yielded more favorable results. In addition, the need for alternative neuropsychological tests should be considered. Selecting the MMSE as the primary end point was mainly due to the lack of clinical studies using assessments other than the MMSE to evaluate cilostazol’s association with cognition^27^ when the present study was initially planned. However, a recent study by some of us^30^ reported that transitioning from cilostazol to 3,4-dehydrocilostazol was associated with preserving cognitive function, as evaluated by the Montreal Cognitive Assessment. Mild cognitive impairment is a complex condition encompassing various pathological factors, including vascular diseases^20,51^; therefore, neuropsychological tests more sensitive to information-processing speed and executive function may be needed in future trials.

Another study by some of us,^44^ reported that patients with AD demonstrated significantly lower levels of the blood Aβ-albumin complex in their blood than age-matched control individuals without cognitive impairment, although free Aβ concentrations in the blood did not differ between patients with AD and controls. The Aβ-albumin complex blood levels were positively associated with the Aβ concentration and negatively correlated with phosphorylated tau in the cerebrospinal fluid.^44^ Circulating Aβ peptides are predominantly bound to plasma proteins and erythrocytes. Interaction of Aβ peptide to plasma proteins, including albumin, can occur rapidly and remain stable for more than 24 hours.^52,53^ Therefore, in the present study, we measured the blood’s Aβ-albumin complex levels and observed a mean (SE) 3-fold increment, without statistical significance, in the cilostazol group (3.95 [1.10] mg/mL) compared with the placebo group (1.22 [0.89] mg/mL). This result may indicate that cilostazol affected the blood levels of the Aβ-albumin complex, potentially facilitating the clearance of Aβ. Our initial hypothesis was that cilostazol would promote Aβ clearance through the intramural periarterial drainage from the brain to the blood, leading to an increase in the concentration of Aβ-albumin complex levels in the blood. While we did observe this trend in the COMCID study, these results should be interpreted with caution. First, we did not conduct cerebrospinal fluid analysis or positron emission tomography analysis, which limited our ability to draw direct conclusions about the Aβ metabolism in the brain. Second, recent advances in mass spectrometry and highly sensitive immunoassays may provide a more accurate evaluation of blood Aβ levels.^32,54^ Therefore, the impact of cilostazol on Aβ metabolism will require further investigation.

### Limitations

The present study has several limitations. First, this study was conducted exclusively in Japan, and multinational validation studies are required. Second, we did not perform apolipoprotein E (*APOE*) genotyping; the *APOE* ε4 allele has been associated with AD conversion and accelerated hippocampal atrophy,^55^ although hippocampal atrophy has been associated with both AD and vascular cognitive impairment.^56^ Novel variants associated with dementia have been continuously reported,^57^ and genome-based medicine is a major challenge in future studies. Third, amyloid and tau positron emission tomography were not performed. Mild cognitive impairment does not always mean prodromal AD, and multiple pathological complexes should be considered.^20,51^ We hypothesized that cilostazol would be effective for both AD and vascular cognitive impairment^31^ because its antiplatelet effect has been associated with the prevention of ischemic stroke.^19,20^ However, the etiology of MCI can involve various neurodegenerative diseases. Future trials should account for these potential cofounders. Fourth, only a fraction of cilostazol can penetrate the BBB,^58^ limiting its target engagement. For inhibiting Aβ production in neural cells, cilostazol needs to traverse the BBB. However, for promoting Aβ clearance through the intramural periarterial drainage or preventing ischemic stroke, it may not necessarily need to traverse the BBB. The precise mechanism underlying cilostazol’s protective effects on AD and CVD should be further investigated.

## Conclusions

The findings of this randomized clinical trial demonstrated that cilostazol was safe and well tolerated among adults in Japan with MCI. However, it did not prevent cognitive decline. The efficacy of cilostazol should be tested in future trials.

## Notes

Supplement 1.
Trial Protocol

Supplement 2. **eTable 1.** Eligibility Criteria **eTable 2.** Drug Adherence Rate **eTable 3.** Change in MMSE Scores From Baseline in Stratified Patients **eFigure 1.** Schedule of Efficacy Assessments **eFigure 2.** Effects of Cilostazol on Psychological Tests
eMethods.

eReferences

Supplement 3. **Nonauthor Collaborators.** Nonauthor members of the COMCID Trial Investigator Group

Supplement 4.
Data Sharing Statement
