# Supplementary material for: Efficacy and Safety of Cilostazol in Mild Cognitive Impairment: A Randomized Clinical Trial
Source: JAMA Netw Open. 2023 Dec 4;6(12):e2344938. doi: 10.1001/jamanetworkopen.2023.44938 (PMC10696485; doi:10.1001/jamanetworkopen.2023.44938)
Supplement: Supplement 1. — Trial Protocol [file jamanetwopen-e2344938-s001.pdf]

Supplement 1

Efficacy and safety of cilostazol in mild cognitive impairment: A randomized clinical trial

Satoshi Saito, Keisuke Suzuki, Ryo Ohtani, Takakuni Maki, Hisatomo Kowa, Hisatsugu Tachibana, Kazuo Washida, Nobuya Kawabata, Toshiki Mizuno, Rie Kanki, Shinji Sudoh, Hiroshi Kitaguchi, Katsuro Shindo, Akihiro Shindo, Nobuyuki Oka, Keiichi Yamamoto, Fumihiko Yasuno, Chikage Kakuta, Ryosuke Kakuta, Yumi Yamamoto, Yorito Hattori, Yukako Takahashi, Yuriko Nakaoku, Shuichi Tonomura, Naoya Oishi, Toshihiko Aso, Akihiko Taguchi, Tatsuo Kagimura, Shinsuke Kojima, Masanori Taketsuna, Hidekazu Tomimoto, Ryosuke Takahashi, Hidenao Fukuyama, Kazuyuki Nagatsuka, Haruko Yamamoto, and Masanori Fukushima, Masafumi Ihara, the COMCID Trial Investigator Group

- English translated edition of the COMCID trial protocol
- Original trial protocol
- Original statistical analysis plan

This document is a translated edition of the original trial protocol described in Japanese that was approved by the institutional review board.

English version

RESEARCH STUDY PROTOCOL

A Multicenter, Randomized Controlled Trial of Cilostazol in Patients with Mild Cognitive Impairment: A Trial Protocol

Protocol No. TRINEU1321

Drug development phase: 2

Principal Investigator: Masafumi Ihara, MD, PhD, FACP

National Cerebral and Cardiovascular Center

Phone number: +81-6-6833-5012

Fax number: +81-6-6833-5137

Issue Date: July 2nd, 2018

Protocol Synopsis

**Study Title**	**A Multicenter, Randomized Controlled Trial of Cilostazol in Patients with Mild Cognitive Impairment: A Trial Protocol**
**Study Phase**	Phase 2
**Protocol number**	TRINEU1321
**Study Period**	Between May 2015 and March 2020
**Study Population**	A total of 200 patients with mild cognitive impairment (MCI) were enrolled and followed-up for 2 years.
**Primary Study Objective**	**The Primary Objective of the study** is:<br>To evaluate the efficacy of cilostazol with respect to the cognitive function measured by Mini-Mental State Examination (MMSE) in patients with MCI.
**Secondary and Exploratory Study Objectives**	**The Secondary Objectives of the study** are to:<br>• evaluate the efficacy of cilostazol in preventing the conversion from MCI to “all-cause dementia”.<br>• evaluate the efficacy of cilostazol with respect to the cognitive function as measured by Clinical Dementia Rating-Sum of Boxes (CDR-SB), Alzheimer's Disease Assessment Scale-Japanese cognitive subscale (ADAS-Jcog/14), and Wechsler Memory Scale-Revised (WMS-R) logical memory in patients with MCI.<br>• evaluate the efficacy of cilostazol with respect to the activity of daily living measured by Alzheimer's Disease Cooperative Study-MCI-Activities of Daily Living (ADCS-MCI-ADL) in patients with MCI.

	• evaluate the efficacy of cilostazol with respect to the hippocampal volume measured by brain MRI in patients with MCI.<br><br>**The Exploratory Objectives** are to:<br>• explore the efficacy of cilostazol with respect to the instrumental activities of daily living of ADCS-MCI-ADL in patients with MCI.<br>• explore the efficacy of cilostazol with respect to the performance of Trail Making Test (TMT) Part A and Part B in patients with MCI.<br>• evaluate the efficacy of cilostazol with respect to the cognitive function measured by the Free and Cued Selective Reminding Test (FCSRT) in patients with MCI.<br>• evaluate the efficacy of cilostazol with respect to the serum levels of albumin-Aβ complexes in patients with MCI.<br><br>**Safety of cilostazol is also evaluated in patients with MCI.**
**Analyses & Study Variables**	The following key analyses, among others, will be performed on the data collected from the database:<br>Primary Analysis<br>• Changes in MMSE from the baseline<br><br>Secondary Analysis<br>• Time to conversion from MCI to “all-cause dementia”<br>• Changes in CDR-SB, ADAS-Jcog/14, or WMS-R logical memory

	from the baseline<br>• Changes in ADCS-MCI-ADL from the baseline<br>• Changes in hippocampal volume measured by MRI from the baseline<br><br>Exploratory Analysis<br>• Changes in the ADCS-MCI-ADL questions 19–24 from the baseline<br>• Changes in the scores from TMT Part A and Part B from the baseline<br>• Changes in the scores from the FCSRT from the baseline<br>• Changes in the serum levels of albumin-Aβ complexes from the baseline<br><br>Safety Analysis<br>• AEs
**Study Design**	Phase II Trial; Parallel Study; Concurrent placebo control; Randomized; Double Blind
**Sample Size**	A total of 200 participants comprising 100 participants for the Investigational Treatment arm and 100 participants for the Comparative Treatment arm
**Inclusion Criteria**	• Age between 55–84 years (inclusive)<br>• Patients with MCI–Core clinical criteria based on NIA/AA classification:<br>i. Memory complaint by a participant or study partner

	Type I: Memory complaint by a participant that is verified by a study partner<br>Type II: Memory complaint by a study partner with the evidence of memory impairment<br>Note: Memory complaint by a participant that is not verified by a study partner will be excluded<br>ii. MMSE scores between 22 and 28 (inclusive)<br>iii. CDR = 0.5<br>• Study partner who sufficiently knows the daily life activities of the patient<br>• Written informed consent provided for participation in the study
**Exclusion Criteria**	• Existence of Parkinson's disease, Huntington's disease, normal pressure hydrocephalus, progressive supranuclear palsy, epilepsy, multiple sclerosis, cerebral infection, neurosyphilis, or any subsequent complications caused by head trauma<br>• Cerebral multiple infarcts, brain tumor, or subdural hematoma on an MRI performed within 48 weeks of provisional registration<br>• Contraindications for MRI such as metal or magnetic implants in the body<br>• History of major depression or bipolar disorder within 48 weeks of provisional registration, a history of alcohol or other substance abuse within 96 weeks of provisional registration, or having other diseases or unstable conditions<br>• Cognitive impairment resulting from abnormal thyroid function or deficiency of vitamin B_{12} or folate<br>• Use of anti-dementia drugs, psychoactive drugs, oral anticoagulants,

	or more than two types of antiplatelet drugs within 4 weeks of provisional registration<br>• Existence of poorly controlled diabetes mellitus (HbA1c > 9.0%) or treated with insulin within 4 weeks of provisional registration<br>• Episode of hypoglycemic attack with loss of consciousness within 4 weeks of provisional registration<br>• Participation in any other drug study for Alzheimer's disease<br>• Existence of bleeding disorders, congestive heart failure, or coronary artery stenosis<br>• Sustained high blood pressure within 2 weeks of provisional registration<br>• History of drug hypersensitivity to cilostazol<br>• Pregnant or breast-feeding at the time of provisional registration<br>• Difficulty in conducting neuropsychological tests because of hearing or visual impairments<br>• Being considered by a principal investigator to be inappropriate for participation in this trial for any other reason
**Study Intervention and Follow-up**	Investigational treatment with cilostazol 50 mg twice a day per oral for 96 weeks vs. comparative treatment with placebo twice a day per oral for 96 weeks

Table of Contents

1 Introduction....10
2 Study Rationale and Objectives....12
2.1 Study Rationale....12
2.2 Study Objectives....15
3 Study Design....16
3.1 Type/Design of Study....16
3.2 Study Population....16
3.3 Eligibility Criteria....17
3.4 Primary and Secondary Endpoints....21
3.5 Randomization....22
3.6 Study Procedures....22
3.7 Schedule of Assessments....27
3.8 End of Study....29
3.9 Stopping Rules, Withdrawal Criteria, and Procedures....29
3.10 Screen Failures....31
3.11 Definition of Completed Participants....31
3.12 Definition of Lost to Follow-up....31
3.13 Participant Compliance....31
3.14 Protocol Deviations....33
4 Restrictions....34
4.1 Prohibited Medications....34
4.2 Contraindications and Precautions of Cilostazol....35
4.3 Other Restrictions....36
5 Collection and Reporting of Serious and Non-serious Adverse Events (AEs)....36
5.1 Definitions of Serious and Non-serious AE....36

5.2 Collection and Reporting of Safety Information 37
5.3 Data Collection Timeframe 40
6 Statistical Analysis 42
6.1 Analysis Set 42
6.2 Analyses and Methods 43
6.3 Sample Size 45
6.4 Source Documents 45
6.5 Data Collection 47
6.6 File Management at the Study Site 48
7 Quality Control and Quality Assurance 49
7.1. Quality Control 49
7.2 Quality Assurance 50
8 Ethics and Responsibility/Conflicts of Interest 51
8.1 Compliance with the GCP and the Applicable Regulatory Requirement 51
8.2 Compliance with the Study Protocol 51
9 Confidentiality 52
10 Amendment Policy 52
11 Other Support 53
11.1 Funding Resources and Conflict of Interest 53
11.2 Expenses of the Study 53
11.3 Compensation for Study-related Health Damages 53
12 Publication Plan 54

List of Abbreviations and Definitions of Terms

**Abbreviation**	**Definition**
Abeta	Amyloid beta (β)
AD	Alzheimer’s disease
ADCS-ADL	Alzheimer's Disease Cooperative Study-Activities of Daily Living
ADAS-JCog	Alzheimer's Disease Assessment Scale-Japanese cognitive subscale
AE	Adverse Event
BMI	Body Mass Index
CDR	Clinical Dementia Rating
CRF	Case Report Form
FAS	Full Analysis Set
FCSRT	Free and Cued Selective Reminding Test
GCP	Good Clinical Practice
IRB	Institutional Review Board
MCI	Mild cognitive impairment
MMSE	Mini-Mental State Examination
MP-RAGE	Magnetization-prepared rapid gradient-echo
MRI	Magnetic Resonance Imaging
PPS	Per Protocol Set
QALY	Quality Adjusted Life years
SAE	Serious Adverse Event
SAS	Safety Analysis Set
TMT	Trail Making Test
WMS-R	Wechsler Memory Scale-Revised

1 Introduction

To date, the effective methods of preventing the progression of mild cognitive impairment (MCI) to diseases entailing dementia, including Alzheimer's disease (AD), have not been established. Furthermore, the treatment modalities of AD that inhibit beta/gamma secretase to decrease the production of amyloid β (Abeta) or those that solubilize Abeta with immunotherapy have not been proven effective. Instead, facilitated clearance of Abeta in sporadic AD is one of the main targets to be considered[1]. A phase IIb trial using RAGE inhibitor that facilitates Abeta clearance in the patients with mild-to-moderate AD was reported to be promising, supporting the above concept[2]. Cilostazol has been shown to promote the clearance of Abeta through the perivascular drainage pathway[3], which could provide different approach to prevent the AD progression.

In our rodent pre-clinical research, cerebral hypoperfusion accelerated the deterioration of cognitive function with increased deposition of Abeta[4,5]. In contrast, administration of cilostazol, a phosphodiesterase III inhibitor, prevented the deposition of Abeta and improved the cognitive function by increasing the hemodynamic reserve and facilitating the perivascular drainage of Abeta[3]. In the Nun study, the presence of one or two small infarction lesions in the brain increased the risk of AD by 20 times[6]. Therefore, treatment that restores vascular integrity can promote Abeta clearance to prevent the conversion from MCI to diseases entailing dementia, including AD.

The above findings suggest that the preemptive medicine for dementia can be established using cilostazol to target patients with MCI.

References

1. Weller RO, Subash M, Preston SD, Mazanti I, Carare RO. Perivascular drainage of amyloid-beta peptides from the brain and its failure in cerebral amyloid angiopathy and Alzheimer’s disease. *Brain Pathol.* 2008;18(2):253-266.

2. Burstein AH, Grimes I, Galasko DR, Aisen PS, Sabbagh M, Mjalli AM. Effect of TTP488 in patients with mild to moderate Alzheimer's disease. *BMC Neurol*. 2014;14(1):12.

3. Ihara M, Okamoto Y, Carare RO, Hase Y, Hattori Y, Hawkes C, Saito S, Taguchi A, Takahashi R, Miyakawa T, Kalaria RN, Lo EH, Arai K, Ihara M. *Ann Clin Trans Neurol*, in revision.

4. Yamada M, Ihara M, Okamoto Y, et al. The influence of chronic cerebral hypoperfusion on cognitive function and amyloid β metabolism in APP overexpressing mice. *PLoS One*. 2011;6(1):e16567.

5. Okamoto Y, Yamamoto T, Kalaria RN, et al. Cerebral hypoperfusion accelerates cerebral amyloid angiopathy and promotes cortical microinfarcts. *Acta Neuropathol.* 2012;123(3):381-394.

6. Snowdon DA, Kemper SJ, Mortimer JA, Greiner LH, Wekstein DR, Marksebery WR. Linguistic ability in early life and cognitive function and Alzheimer's disease in late life: findings from the Nun Study. *JAMA.* 1996;275(7):528-532.

2 Study Rationale and Objectives

2.1 Study Rationale

Epidemiological, clinicopathological, and animal studies show that vascular disease in various forms contributes to the cognitive decline[1,2]. Increasing age is the strongest risk factor for dementia irrespective of whether it results from a vascular etiology or neurodegenerative disease processes such as in AD. AD and vascular cognitive impairment (VCI), the two most common causes of dementia, represent two extremes of a spectrum of disorders; however, a number of entities, which possess varying degrees of neurodegenerative and vascular pathologies, occur in between[3]. The pure forms of the disorders are preferred for the convenience of labeling, treating, or managing the conditions, but conditions within the spectrum are the norm rather than the exception as dementia advances[4]. Therefore, combinatorial therapy directed at both vascular and neurodegenerative aspects of dementia may be a promising approach for the treatment of dementia in the elderly patients.

Cilostazol acts as an antiplatelet agent and has other pleiotropic effects mediated by phosphodiesterase-3-dependent mechanisms[5]. Increasing evidence suggests that cilostazol offers endothelial protection, via inhibition of apoptosis in endothelial cells[6], attenuates the phenotypic modulation of vascular smooth muscle cells[7], and sustains blood flow by endothelium-independent vasodilation[8]. Intriguingly, cilostazol decreased Abeta accumulation and protected Abeta-induced cognitive deficits in an experimental model[9,10]. In a pilot study of 10 patients with moderate AD who received donepezil, cilostazol add-on treatment for 5–6 months demonstrated significantly increased MMSE score compared to the baseline treatment[11]. Moreover, cilostazol was effective in halting cognitive decline in patients with AD having cerebrovascular diseases[12], mild cognitive

impairment[13], and mild dementia who received donepezil[14].

These results highlight the need for a comprehensive prospective cohort study to analyze the effect of cilostazol on the preservation of cognitive function in patients with early-stage cognitive impairment, known as MCI.

References

1. Gorelick PB, Scuteri A, Black SE, et al. Vascular contributions to cognitive impairment and dementia: a statement for healthcare professionals from the American Heart Association/American Stroke Association. *Stroke.* 2011;42(9):2672-2713.
2. Toledo JB, Arnold SE, Raible K, et al. Contribution of cerebrovascular disease in autopsy confirmed neurodegenerative disease cases in the National Alzheimer's Coordinating Centre. *Brain.* 2013;136(Pt9):2697–2706.
3. Kalaria RN, Akinyemi R, Ihara M. Does vascular pathology contribute to Alzheimer changes*? J Neurol Sci.* 2012;322(1-2):141–147.
4. Kalaria RN, Ihara M. Dementia: Vascular and neurodegenerative pathways-will they meet? *Nat Rev Neurol*. 2013;9(9):487-488.
5. Liu Y, Shakur Y, Yoshitake M, Kambayashi Ji J. Cilostazol (pletal): a dual inhibitor of cyclic nucleotide phosphodiesterase type 3 and adenosine uptake. *Cardiovasc Drug Rev.* 2001;19(4):369-386.
6. Kim KY, Shin HK, Choi JM, Hong KW. Inhibition of lipopolysaccharide-induced apoptosis by cilostazol in human umbilical vein endothelial cells. *J Pharmacol Exp Ther.* 2002;300(2):709-715.

7. Fujita Y, Lin JX, Takahashi R, Tomimoto H. Cilostazol alleviates cerebral small-vessel pathology and white-matter lesions in stroke-prone spontaneously hypertensive rats. *Brain Res.* 2008;1203:170-176.
8. Tanaka K, Gotoh F, Fukuuchi Y, et al. Effects of a selective inhibitor of cyclic AMP phosphodiesterase on the pial microcirculation in feline cerebral ischemia. *Stroke.* 1989;20(5):668-673.
9. Hiramatsu M, Takiguchi O, Nishiyama A, Mori H. Cilostazol prevents amyloid β peptide(25-35)-induced memory impairment and oxidative stress in mice*. Br J Pharmacol.* 2010;161(8):1899-1912.
10. Park SH, Kim JH, Bae SS, et al. Protective effect of the phosphodiesterase III inhibitor cilostazol on amyloid β-induced cognitive deficits associated with decreased amyloid β accumulation. *Biochem Biophys Res Commun*. 2011;408(4):602-608.
11. Arai H, Takahashi T. A combination therapy of donepezil and cilostazol for patients with moderate Alzheimer disease: pilot follow-up study. *Am J Geriatr Psychiatry.* 2009;17(4):353-354.
12. Sakurai H, Hanyu H, Sato T, et al. Effects of cilostazol on cognition and regional cerebral blood flow in patients with Alzheimer’s disease and cerebrovascular disease: a pilot study. *Geriatr Gerontol Int*. 2013;13(1):90-97.
13. Taguchi A, Takata Y, Ihara M, et al. Cilostazol improves cognitive function in patients with mild cognitive impairment: A retrospective analysis. *Psychogeriatrics*. 2013;13(3):164-169.
14. Ihara M, Nishino M, Taguchi A, et al. Cilostazol add-on therapy in patients with mild dementia receiving donepezil: a retrospective study. *PLoS One.* 2014;9(2):e89516.

2.2 Study Objectives

To evaluate the efficacy of cilostazol with respect to the cognitive function measured by MMSE in the patients with MCI.

2.2.1 Primary

- To evaluate the efficacy of cilostazol with respect to the cognitive function measured by MMSE in the patients with MCI.

2.2.2 Secondary

- To evaluate the efficacy of cilostazol in preventing conversion from MCI to “all-cause dementia” in the patients with MCI
- To evaluate the efficacy of cilostazol with respect to the cognitive function measured by CDR-SB, ADAS-JCog/14, or WMS-R logical memory in patients with MCI
- To evaluate the efficacy of cilostazol with respect to the activity of daily living measured by ADCS-MCI-ADL in patients with MCI
- To evaluate the efficacy of cilostazol with respect to the hippocampal volume measured by brain MRI in patients with MCI

2.2.3 Exploratory

- To explore the efficacy of cilostazol with respect to the instrumental activities of daily living of ADCS-MCI-ADL in patients with MCI
- To explore the efficacy of cilostazol with respect to the performance of Trail Making

Test (TMT) Part A and Part B in patients with MCI

- To evaluate the efficacy of cilostazol with respect to the cognitive function measured by the Free and Cued Selective Reminding Test (FCSRT) in patients with MCI
- To evaluate the efficacy of cilostazol with respect to the serum levels of albumin-Aβ complexes in patients with MCI

3 Study Design

3.1 Type/Design of Study

Trial phase	: Phase II trial
Intervention Model	: Parallel study
Control Type	: Concurrent placebo control
Trial Randomization	: Randomized
Trial Blinding Schema	: Double blind
Single or multicenter	: Multicenter

This is a two-year, randomized, double-blind, placebo-controlled, multicenter, Phase II study to evaluate the efficacy of cilostazol. The participants must be patients with MCI who comply with the inclusion and exclusion criteria listed below. Complying participants will be randomized to receive cilostazol or placebo. The participants will take one tablet twice a day for 96 weeks.

3.2 Study Population

MCI (core clinical criteria: Albert MS, et al. Alzheimers Dement 2011; 7(3): 270–279) is

diagnosed when individuals meet the following criteria:

- Cognitive concern reflecting a change in cognition reported by a patient, informant, or clinician (i.e., historical or observed evidence of decline over time)
- Objective evidence of impairment in one or more cognitive domains, typically including memory (i.e., formal or bedside testing to establish a level of cognitive function in multiple domains)
- Preservation of independence in functional abilities
- Not demented

However, the following conditions should be considered for the diagnosis:

- Rule out vascular, traumatic, and medical causes of cognitive decline, where possible
- Provide evidence of longitudinal decline in cognition, when feasible
- Report history consistent with AD genetic factors, where relevant

3.3 Eligibility Criteria

The current study sets the following inclusion and exclusion criteria to enroll participants with MCI having a clear demarcation.

3.3.1 Informed Consent

i) Preparation of informed consent form

The site investigators should create a written informed consent form and obtain the approval of the Institutional Ethical Committee or Institutional Review Board (IRB). The items which should be listed in the written informed consent form

should be compliant with Article 51 of the Japanese Good Clinical Practice (GCP) for medical product.

ii) Timing and procedure of informed consent

The site investigators or study physicians should orally explain the clinical study precisely to each participant with informed consent form prior to the participation in the clinical trial and obtain the participant's voluntary written informed consent.

The site investigators or study physicians should provide the opportunity for the participant to ask questions prior to obtaining the consent and give sufficient time to consider his or her willingness to participate in the clinical trial. In that regard, the site investigators and a research collaborator, as a supporting elucidator, should answer and address all the questions to satisfy the participant.

The site investigators or study physicians, who conducted the explanation, and the participant will each write the date, his/her name, and seal or his/her signature in the informed consent document. The informed consent document will be issued to the participant and its copy will be attached to a medical record at the institution. Moreover, when a collaborator of the clinical trial supportively explains the protocol, the collaborator should enter his/her name and seal or his/her signature and date in the document.

The site investigators or study physicians should give a copy of the written informed consent to the participant and store the original written informed consent with the medical record in the institute.

iii) Revision of written informed consent form

When the written informed consent form needs to be revised, the site investigators make the revised version of the written informed consent form and submit it to the director of the institution for the approval of the Institutional Ethical Committee/IRB.

After enrollment of the participants in the clinical trial, if the written informed consent form is revised, the site investigators should promptly explain the relative information to the participants each time, confirm the willingness of the patients to participate in the clinical trial, re-explain the revised written informed consent form, and obtain the voluntary consent of the participants to continue participation in the clinical trial in writing.

3.3.2 Inclusion Criteria

The participants are required to meet the following inclusion criteria:

- Age in the range of 55–84 (inclusive)
- Patients with MCI–Core clinical criteria based on NIA/AA classification
  i. Memory complaint by the participant or study partner

     Type I: Memory complaint by a participant that is verified by a study partner

     Type II: Otherwise, memory complaint by a study partner with evidence of memory impairment

     Note: A participant having memory complaint that is not verified by a study partner will be excluded

  ii. MMSE scores in the range of 22–28 (inclusive)

iii. CDR =0.5

- Study partner who sufficiently knows the daily life of the patient
- Written informed consent provided for study participation

3.3.3 Exclusion Criteria

Participants will be excluded if they meet any of the following exclusion criteria prior to randomization.

- Existence of Parkinson's disease, Huntington's disease, normal pressure hydrocephalus, progressive supranuclear palsy, epilepsy, multiple sclerosis, cerebral infection, neurosyphilis, or any subsequent complications caused by head trauma
- Cerebral multiple infarcts, brain tumor, or subdural hematoma on an MRI performed within 48 weeks of provisional registration
- Contraindications for MRI such as metal or magnetic implants in the body
- History of major depression or bipolar disorder within 48 weeks of provisional registration, history of alcohol or other substance abuse within 96 weeks of provisional registration, or having other diseases or unstable conditions
- Cognitive impairment resulting from abnormal thyroid function or deficiency of vitamin B12 or folate
- Use of anti-dementia drugs, psychoactive drugs, oral anticoagulants, or more than two types of antiplatelet drugs within 4 weeks of provisional registration
- Existence of poorly controlled diabetes mellitus (HbA1c > 9.0%) or treated with insulin within 4 weeks of provisional registration
- Episode of hypoglycemic attack with loss of consciousness within 4 weeks of

provisional registration

- Participation in any other drug study for AD
- Existence of bleeding disorders, congestive heart failure, or coronary artery stenosis
- Sustained high blood pressure within 2 weeks of provisional registration
- History of drug hypersensitivity to cilostazol
- Pregnant or breast-feeding at the time of provisional registration
- Difficulty in conducting neuropsychological tests because of hearing or visual impairments
- Being considered by a principal investigator to be inappropriate for participation in this trial for any other reason

3.4 Primary and Secondary Endpoints

3.4.1 Primary Analysis

- Changes in the cognitive function measured by MMSE from the baseline in patients with MCI.

3.4.2 Secondary Analysis

- Time to conversion from MCI to “all-cause dementia” in patients with MCI.
- Changes in the cognitive function measured by CDR-SB, ADAS-JCog/14, or WMS-R logical memory from the baseline in patients with MCI.
- Changes in the activity of daily living measured by ADCS-MCI-ADL from the baseline in patients with MCI.

- Changes in the hippocampal volume measured by brain MRI from the baseline in patients with MCI.

3.4.3 Exploratory Analysis

- Changes in ADCS-MCI-ADL questions 19–24 from the baseline in patients with MCI.
- Changes in scores from TMT Part A and Part B from the baseline in patients with MCI.
- Changes in scores from the FCSRT from the baseline in patients with MCI.
- Changes in the serum levels of albumin-Aβ complexes from the baseline in patients with MCI.

3.4.4 Safety Analysis

- AEs

3.5 Randomization

Participants are randomly allocated to the cilostazol or control arm. The allocation method is minimization. The stratification factors are age (older or younger than 75 years), sex, education (more or less than 12 years), and institutions. The detailed randomization procedure will be done following the Standard Operational Procedure of Randomization (SOP).

3.6 Study Procedures

3.6.1 Treatment of Subjects

After the registration, the site investigators should start the protocol treatment within 14 days including the day of registration.

Protocol treatment is defined as follows;

Investigational Treatment:

Cilostazol 50mg twice a day per oral for 96 weeks

Comparative Treatment:

Placebo twice a day per oral 96 for weeks

(Justification for the dosage of cilostazol)

Arai (Juntendo University) *et al.* reported that cilostazol (100 mg/day) was added to the donepezil treatment in 10 patients with AD, and 8 of the 10 patients showed an improved MMSE score at 5–6 months in comparison with that at baseline (mean follow-up, 7.4 months)[1].

Taguchi *et al*. surveyed medical records to select all the cases that had administered cilostazol and undergone MMSE evaluation at least twice at an interval of more than 6 months by a retrospective analysis of 3,183 consecutive patients in a community hospital between 1996 and 2012. Patients who had administered anti-dementia drugs were excluded. They compared the temporal change in MMSE score between the patients who were treated with cilostazol (N=70) and those who ceased administration (N=22). The mean follow-up period was 691 days. They reported that the decrease in

MMSE was significantly suppressed by the administration of cilostazol. Subgroup analysis revealed that cilostazol improved MMSE in the patients with mild cognitive impairment (0.4 ± 0.7/year), though the decrease was observed in patients who ceased cilostazol (-3.8 ± 1.3/year)[2]. There were no differences in the annual MMSE decrease between the patients receiving cilostazol in a dose of 200 mg/day and those receiving cilostazol in a dose of 100 mg/day.

Ihara *et al.* selected from the same records all the cases that had administered donepezil with or without cilostazol (taken for more than 6 months) and undergone MMSE evaluation at least twice at an interval of more than 12 months. They compared the temporal change in MMSE score between the patients who were treated with donepezil alone (N=87) and those who were treated with donepezil plus cilostazol (N=69). The decrease in MMSE did not show a significant suppression by co-administration of cilostazol in those patients. However, subgroup analysis of the patients with mild cognitive impairment (22≤MMSE≤26) showed that cilostazol suppressed the MMSE change (N=34; -0.45 ± 0.28/year), while a more pronounced decrease was observed in the patients who received donepezil alone (N=36; -2.23 ± 0.69/year). These results suggest that cilostazol suppressed the cognitive decline in the patients with mild dementia receiving donepezil[3]. Moreover, there were no differences in the annual MMSE decrease between the patients receiving cilostazol in a dose of 200 mg/day and those receiving cilostazol in a dose of 100 mg/day.

In addition, for prevention of recurrence of cerebral infarction (except for cardioembolic ischemic stroke), the dosage of cilostazol is 100 mg twice a day per oral in Japan and 50 mg or 100 mg twice a day per oral in China. This fact supports the safety of the long-term administration of cilostazol in a dose of 50 mg twice a day per oral.

References

1. Arai H and Takahashi T. A combination therapy of donepezil and cilostazol for patients with moderate Alzheimer disease: pilot follow-up study. *Am J Geriatr Psychiatry*. 2009;17(4):353-354.
2. Taguchi A, Takata Y, Ihara M, et al. Cilostazol improves cognitive function in patients with mild cognitive impairment: a retrospective analysis. *Psychogeriatrics*. 2013;13(3):164-9.
3. Ihara M, Nishino M, Taguchi A, et al. Cilostazol add-on therapy in patients with mild dementia receiving donepezil: a retrospective study. *PLoS One*. 2014;26;9(2):e89516.

3.6.2 Modification of dosage and schedule

If dose reduction is needed, the participant should be regarded as early termination. The site investigator or study physician may suspend the study drug administration within a week because of medical treatment, endoscopy, or other medical requirement.

Table Schedule of Assessments

Assessment / Period	Informed consent	Baseline/ Screening	Visits					
			4 weeks	24 weeks	48 weeks	72 weeks	96 weeks	Early termination
Informed consent	X							
Demographic information		X						
Neurological examination		X	X	X	X	X	X	X
Laboratory examination		X	X	X				
Neuroimaging study (MRI)※		X					X	X
Albumin-Aβ complexes		X		X				
MMSE		X		X	X	X	X	X
CDR, ADAS-JCog, WMS-R,TMT, FCSRT※		X			X		X	X
ADCS-MCI-ADL		X			X		X	X
Confirmation of compliance			←─	──	──	──	──	─→
Adverse events			←─	──	──	──	──	─→
Outcome assessment			←─	──	──	──	──	─→
Permissible range			Within 4 weeks	±2 weeks	±2 weeks	±2 weeks	±2 weeks	+4 weeks

※：Not mandatory

3.7 Schedule of Assessments

The participants will be assessed in accordance with the schedule of assessments listed below. All data in this study will be collected through web-based electronic data capture systems.

3.7.1 Baseline

The participants will be observed for a baseline visit upon meeting the inclusion and exclusion criteria detailed in Section 3.3. During this initial baseline visit, the following assessments will be conducted:

- Signing of informed consent form
- Assessing meeting the inclusion/exclusion criteria
- Collection of demographic Information
- Neurological examination
- Laboratory examination (complete blood counts, biochemical analysis, urinalysis)
- Neuropsychological tests (MMSE, CDR, ADAS-JCog, WMS-R logical memory, TMT, FCSRT [not mandatory])
- ADCS-MCI-ADL
- MRI (not mandatory)
- Albumin-Aβ complexes
- Adverse Events

3.7.2 Four-Week Follow-Up Visits

Following baseline, the participants will be observed within 4 weeks. The following

assessments will be conducted:

- Neurological examination
- Laboratory examination (complete blood counts and biochemical analysis)
- Adverse Events

3.7.3 Follow-Up Visits after 24, 48, 72, and 96 Weeks

The participants will be assessed during follow-up visits every 24 weeks (± 2 weeks).

During the follow-up visits, the following assessments will occur:

- Neurological examination
- Laboratory examination (biochemical analysis at the 24-week follow-up visit only)
- MMSE
- CDR, ADAS-JCog, WMS-R logical memory, TMT, FCSRT (not mandatory, at the 48 and 96-week follow-up visits only)
- ADCS-MCI-ADL (at the 48 and 96-week follow-up visits only)
- MRI (not mandatory, at the 96-week follow-up visit only)
- Albumin-Aβ complexes (at the 24-week follow-up visit only)
- Adverse Events

3.7.4 Study Assessment Methods

- Demographic information–body weight and height; BMI (automatic calculation); familial history of AD; educational level; past medical history such as stroke, myocardial infarction, brain concussion, and general anesthesia; medical complications, such as hypertension, diabetes, and depression; past medication

record; history of alcohol intake and smoking

- Neurological examination–presence of aphasia and apraxia
- MRI assessments–3D-T1 (MPRAGE), fluid attenuated inversion recovery image, T2*-weighted image

3.8 End of Study

The end of study date is defined as the last date of contact or the date of final contact attempt from the follow-up Case Report Form (CRF) page for the last participant completing or withdrawing from the study.

After completing the observations, tests, and investigations specified in this study protocol for all the participants, the site investigator shall report to the director of the institution in writing that the study has been completed and provide a summary of the study results.

The director of the institution shall promptly report to the IRB in writing that the study has been completed and provide a summary of the study results based on the report submitted by the site investigator.

3.9 Stopping Rules, Withdrawal Criteria, and Procedures

3.9.1 Criteria for Study Withdrawal

The site investigator shall withdraw the study in the following cases:

1) When the site investigator and principal investigator judge that it is difficult to continue the study due to problems associated with the quality, efficacy, or safety of the study drug.

2) When the director of the institution issues a directive to withdraw the study based on

the information as shown in number 1) above.

3) When the Data and Safety Monitoring Committee recommends withdrawing the study based on the results of the interim analysis or information as shown in 1) above.

3.9.2 Procedure of Study Withdrawal

The site investigator shall call for a consultation on study withdrawal immediately after the occurrence of an event that meets the criteria for study withdrawal. When a decision is made to withdraw the study, the principal investigator shall immediately report to all investigators, as well as to the Data and Safety Monitoring Committee, and the study drug provider.

The site investigator shall immediately explain to the participant that the study will be withdrawn, the reason for withdrawal, and ensure the safety of the participant.

The site investigator shall provide the information to the director of the institution, IRB, and relevant divisions of the institution, and shall follow the procedures specified by the institution.

3.9.3 Individual Subject

When the following matters are observed, the site investigator discontinues the clinical trial and records the date of discontinuation, reasons for discontinuation, and date when the participant's survival was finally confirmed in a case report form (at the time of discontinuation).

3.9.4 Treatment of Discontinued Cases

Except in the following cases, the investigation is continued with respect to adverse events and outcome measurement.

1) When a subject died.
2) When a follow-up is difficult for any other reasons.

3.10 Screen Failures

Screen failures will only be collected in the event the participant signs the consent form and it is later determined they do not meet the inclusion criteria defined in Section 3.3.2 or meet the exclusion criteria defined in Section 3.3.3.

3.11 Definition of Completed Participants

Participants who are evaluated at the last scheduled visit are defined as study completers.

3.12 Definition of Lost to Follow-up

Participants who cannot be contacted on or before the 96-week visit and who do not have a known reason for discontinuation (e.g., withdrew consent or death) will be classified as “lost to follow-up” as the reason for discontinuation.

3.13 Participant Compliance

The principal investigator, site investigators, site physicians, clinical research coordinators, and study drug administrators should maintain the study compliance of the participants according to the list described below.

1) Instruction of medication
2) Trial visit
3) Safety monitoring before the trial period

Study Compliance of the Participants

The site investigators or study physicians should maintain the drug inventory record of the supplied, received, dispensed, destroyed, and returned medication and should assess the study compliance graded using a 3-grade scale as defined below. The assessed study compliance should be documented on a medical record and CRF. When the study drug is suspended, the study compliance should be assessed according to the planned amount of dosage.

Grading scale of the study compliance of the participants

1） The actual amount of the study drug administration is more than 80% of the planned one
2） The actual amount of the study drug administration is less than 80% but more than 50% of the planned one
3） The actual amount of the study drug administration is less than 50% of the planned one

Concomitant medication and therapy

Any medication other than the prohibited medications, diet therapy, and rehabilitation therapy are not restricted within the study period.

Regardless of the prohibition status, the drug name (generic name), daily dose, and start and end dates of each medication should be documented in the medical record and CRF.

The objectives and route of the administration should also be documented in the CRF.

3.14 Protocol Deviations

The site investigator shall not deviate from and/or change the study protocol without obtaining written approval based on a prior review by the IRB except in the following cases:

1） When such deviation and/or change is necessary to avert immediate risk to the participant or for any other unavoidable clinical reasons.

2） When such change is made due to minor administrative aspects of the study.

In the above-mentioned case number 1) the site investigator must provide details on and reasons for such deviation and/or change, as well as a proposal to revise the study protocol, if appropriate, to the director of the institution and the IRB as soon as possible, and obtain approval.

The site investigators shall promptly report to the director of the institution and IRB in writing any change in the study which may have a critical impact on the study conduct and/or increase the risk to the participants.

The site investigator shall keep and maintain records on all deviations from the study protocol.

4 Restrictions

4.1 Prohibited Medications

Any diet and rehabilitation therapy is not restricted within the study period.

Regardless if its prohibition status, the drug name (generic name), daily dose, and start and end dates of each medication should be documented in the medical record and CRF.

The objectives and route of administration should also be documented in the CRF.

The concomitant use of medications with the below listed mechanism of action should be prohibited from the start to the end date of the study drug administration.

Moreover, any future use of concomitant medications with the below listed mechanism of action should be prohibited from the start date to the end date of the study drug administration.

The use of anti-inflammatory, antipyretic, or analgesic drugs which inhibit platelet aggregation may be allowed within a week.

1) More than two types of drugs which inhibition platelet aggregation
2) Drugs which have anticoagulant action
3) Drugs which have thrombolytic action
4) Drugs for AD
5) Any other study drugs

1） Drugs which inhibit platelet aggregation:

aspirin, dipyridamole, clopidogrel sulfate, ticlopidine hydrochloride, cilostazol, alprostadil, limaprost alfadex, ozagrel sodium, ethyl icosapentate, beraprost sodium, sarpogrelate hydrochloride, and epoprostenol sodium

2） Drugs which have an anticoagulant action:

heparin (without topical external preparation), danaparoid sodium, warfarin potassium, argatroban, ulinastatin, gabexate mesilate, nafamostat mesilate, freeze-dried concentrated human antithrombin III, freeze-dried concentrated human activated protein C, and fondaparinux sodium

3） Drugs which have a thrombolytic action:

alteplase, urokinase, tisokinase, monteplase, pamiteplase, nasaruplase, and batroxobin

4） Drugs for AD:

donepezil, rivastigmine, galanthamine, and memantine

5） Any other study drugs

4.2 Contraindications and Precautions of Cilostazol

1） Contraindications

Cilostazol and several of its metabolites are inhibitors of phosphodiesterase III. Several drugs with this pharmacologic effect have decreased the survival compared to placebo

in patients with class III–IV congestive heart failure.

Cilostazol is contraindicated in patients with:

- congestive heart failure of any severity.
- hemostatic disorders or active pathologic bleeding, such as bleeding peptic ulcer and intracranial bleeding. Cilostazol inhibits platelet aggregation in a reversible manner.
- known or suspected hypersensitivity to any of their components.

2） Precautions

Hematologic Adverse Reactions

Rare cases of thrombocytopenia or leukopenia progressing to agranulocytosis have been reported when cilostazol was not immediately discontinued. However, agranulocytosis was reversible upon discontinuation of cilostazol.

4.3 Other Restrictions

There are no other restricted medications for this protocol.

5 Collection and Reporting of Serious and Non-serious Adverse Events (AEs)

5.1 Definitions of Serious and Non-serious AE

An AE is any untoward medical occurrence in a participant who received a protocol treatment regardless of its causal relationship to the protocol treatment. Therefore, an AE

can be any unfavorable and unintended sign (including an abnormal laboratory finding), symptom, or disease associated with protocol treatment regardless of its causal relationship to the protocol treatment.

Serious adverse event (SAE) is any AE that results in any of the following six outcomes:

1. Death
2. Life threatening condition
3. Requires hospitalization or prolongation of existing hospitalization
4. Disability
5. Congenital disease or birth defect in a later generation
6. Other serious events according to items 1–5

Non-serious AE are all AEs that do not meet the criteria for a SAE.

5.2 Collection and Reporting of Safety Information

5.2.1 Assessment of AE and Criteria

When an AE is detected, the name of the event, date of onset, severity, seriousness, causal relationship, outcome, and date of outcomes should be described in the CRF as an “adverse event”.

5.2.2 Severity of AEs

The severity is determined based on the following criteria.

Mild: events do not interfere with the daily activities of the participant

Moderate: events may cause some interference with the daily activities of the participant

Severe: events interrupt the usual daily activities of the participant

5.2.3 Seriousness of AEs

The seriousness of AEs is determined as follows;.

Yes/no

5.2.4 Causal Relationship of AEs to the Protocol Treatment

The causal relationship of AEs is determined as follows;.

Yes/possible/no

5.2.5 Outcomes of AEs

The outcome of reaction/event is determined as follows;.

Outcome of reaction/event at the time of last observation

− recovered/resolved

− recovering/resolving

− not recovered/not resolved

− recovered/resolved with sequelae

− fatal

− unknown

5.2.6 Reporting Procedure

According to the SOP “collection and reporting of safety information”, when a SAE occurs, the site investigators should report the SAEs to the director of institution and coordinating committee within 24 hours.

The trial coordinating committee should decide whether the SAEs need 7-days reports, 15-days reports (The Pharmaceutical Affairs Law, Enforcement Regulations, Article 273), or no reports. When submission of those report is required, the committee should complete the prespecified submission form and directly submit it to director of the institution and Pharmaceutical and Medical Devices Agency.

Moreover, the trial coordinating committee should inform the other site investigators and pharmaceutical company with the SAE safety information.

The other site investigators should submit the report, which was submitted to Pharmaceutical and Medical Devices Agency, to the director of institution as soon as possible.

When provision of detailed or additional information is requested, the trial coordinating committee and site investigators should submit it to the director of Institution and trial coordinating committee.

5.3 Data Collection Timeframe

5.3.1 Procedure when AE Occurs

When AE occurs, the site investigators or study physicians should adequately treat the participant for his or her safety.

When several events occur, the site investigators or study physicians should evaluate each event.

1. Identification of suspected drug/equipment
2. Severity and seriousness
3. Causal relationship to the protocol treatment
4. Expectedness

5.3.2 AE Requiring Urgent Reporting

1. Death or life-threatening unexpected AE
2. Other unexpected SAE

5.3.3 Follow-up Procedures for the AEs

1. Follow-up procedures for the AEs which were recovered in 10 days after an investigational new drug administration was stopped, where the abnormalities of the laboratory tests returned to the baseline or normal value or the symptom was restored to the preinitiation state, will not be performed.

2. Follow-up investigations for the AEs which did not recover in 10 days after an investigational new drug administration was stopped will be performed within 28 days. A site investigator or study physician will explain the purpose of follow-up investigations to the involved patients and ask for their cooperation.

3. The decision to continue or halt the follow-up investigations for the AEs which did not recover by the follow-up investigations is determined based on the causal relationship to the study drug.

・When the relationship of the AE to the study drug is "no", the subsequent follow-up survey is closed.

・When the relationship of the AE to the study drug is “possible” or "yes", follow-up investigations are continued until the AE recovers or becomes stable.

・When the AE becomes "serious" during the follow-up survey or when a SAE newly develops, the procedure explained in the section "5.2.6 Reporting Procedure" is followed.

・When the relationship of the AE to the study drug is "no", subsequent follow-up investigations are terminated.

・When the relationship of the AE to the study drug is "possible" or "yes", follow-up investigations are continued until the AE recovers or becomes stable.

5.3.4 Expectedness of AE

See the investigator’s brochure regarding the expected AEs in this study.

6 Statistical Analysis

6.1 Analysis Set

6.1.1 Analysis Set

To evaluate the primary and secondary endpoints of this study, clinical efficacy may be separately analyzed using the full analysis set (FAS) and per-protocol set (PPS), if necessary.

AEs and other observations shall be analyzed using the safety analysis set (SAS).

These analysis sets are defined as follows.

6.1.2 FAS and PPS

According to the intention-to-treat principle, FAS is defined as data obtained from the group of registered participants who receive at least part of the assigned treatment. However, if a participant is classified as noneligible after the registration, they shall be excluded from the FAS.

PPS is defined as data obtained from the group of registered participants who strictly follow the procedures described in the protocol.

6.1.3 SAS

SAS is defined as data obtained from the group of registered participants who receive at least part of the assigned treatment.

6.2 Analyses and Methods

The following description represents an overview, whereas the detailed procedures are mentioned in the Statistical Analysis Plan.

6.2.1 Primary Analysis

The change in the MMSE scores between baseline and at 96 weeks shall be tested to evaluate the improvement in cognitive function as a result of the treatments. Furthermore, changes between the baseline and each visit (at 24, 48, 72, and 96 weeks) will be analyzed using a mixed model with repeated measures, with treatments and time considered as fixed effects and subject × time considered as a random effect.

6.2.2 Secondary Analysis

1） Time to conversion from MCI to “all-cause dementia”

The Kaplan–Meier method shall be applied to each group to estimate the survival functions. In addition, comparisons between the groups will be performed using the log-rank test, and the hazard ratios will be estimated using the Cox proportional-hazards model. Furthermore, if bias in the background factors is detected, an analysis using a multivariate Cox proportional-hazards model with background factors as covariates will also be conducted.

2） CDR-SB, ADAS-Jcog, WMS-R

Changes from the baseline to each visit (at 48 and 96 weeks) will be analyzed using a mixed model with repeated measures, with treatments and time considered as fixed

effects and subject × time as a random effect.

3） ADCS-MCI-ADL

Changes from the baseline to each visit (at 48 and 96 weeks) will be analyzed using a mixed model with repeated measures, with treatments and time considered as fixed effects and subject × time as a random effect.

4） Hippocampal volume

The difference between the hippocampal volume measured by MRI at baseline and at 96 weeks after treatment initiation (or discontinuation) shall be compared between the groups using an analysis of covariance with the baseline hippocampal volume as the covariate.

6.2.3 Exploratory Analysis

1） Instrumental activities of daily living: ADCS-MCI-ADL

Changes from baseline to each visit (at 48 and 96 weeks) will be analyzed using a mixed model with repeated measures, with treatments and time considered as fixed effects and subject × time as a random effect.

2） TMT, FCSRT

Changes from baseline to each visit (at 48 and 96 weeks) will be analyzed using a mixed model with repeated measures, with treatments and time considered as fixed effects and subject × time as a random effect.

3） Albumin–Aβ complex concentration

The difference between the serum albumin–Aβ complex concentration at baseline and at 24 weeks after treatment initiation shall be compared between the groups using an analysis of covariance with the baseline concentration as the covariate.

6.2.4 Safety Analysis

Safety shall be evaluated by summarizing the adverse events. All AEs will be coded to the Lowest Level Term using the Medical Dictionary for Regulatory Activities. The occurrence of AEs will be reported based on the Medical Dictionary for Regulatory Activities system organ class and preferred term.

6.3 Sample Size

The sample size, which is 100 evaluable participants per arm, is solely based on feasibility. However, with 90% power, a two-tailed type I error rate of 5%, and an estimate of the standard deviation of 3.7 for MMSE scores, which was observed in the ADNI study in USA, this sample size could detect a statistically significant difference if the effect size is more than 1.72 between two treatments. This sample size determination was followed by Julious[14].

6.4 Source Documents

6.4.1 Identification of Documents for Source Document Verification

The following are the “source documents supporting the data” and “data directly

recorded in case report forms without previous documents or electronic data (data for which the information in the case report form is used as source documents)” specified in this study.

1） Source documents supporting the data

- Records on the consent of participants and provision of information to participants (e.g., written consent with the signature of the participant and patient referral document)

- Medical charts, medical chart labels, or worksheets (documents prepared for this study and attached to the medical chart), nursing records, case registration documents, records on the evaluations/tests/observations specified in this study (e.g., test data, films, test slips), and records on which the case report form is based

- Records on the treatments specified in this study (e.g., study drug accountability records and prescription records)

2） Data for which the information in the case report form is used as source documents

- Evaluation of the inclusion/exclusion criteria

- Severity of past diseases/complications

- Reason for the use of concomitant drug/treatment

- Seriousness, severity, outcome, causal relationship with adverse event, and comments

- Reason for the study discontinuation and comments on outcomes

- Comments from the site investigator

6.5 Data Collection

6.5.1 Preparation of CRFs

The site investigators or study physicians shall prepare CRFs in accordance with the "Guidebook for the Preparation, Modification, or Correction of Case Report Forms".

CRFs shall be prepared, modified, or corrected by the site investigator or study physicians. However, for cases that do not require clinical input, collaborators may record or correct the data in CRFs or electronic data capture system under the supervision of the site investigators or study physicians. The CRFs prepared by the study physicians or study collaborators shall be reviewed by the site investigators for any problems in the data.

6.5.2 Instructions for the Preparation of CRFs

The site investigator or study physicians shall complete the CRFs in a timely manner.

The CRFs shall be modified or corrected in accordance with the "Guidebook for the Preparation, Modification, or Correction of Case Report Forms".

The site investigators shall review the modifications or corrections made to the CRFs by the study physicians.

If any discrepancies between the data in the CRFs and source documents are found, the site Investigators shall record the reasons for such discrepancies and retain the record.

Inquiries on the data in the CRFs shall be answered promptly and, if necessary, modification or correction shall be implemented to the data.

6.6 File Management at the Study Site

6.6.1 Trial Institute

The director of the institution shall retain the documents to be stored at the institution as specified by the GCP.

The storage period of the documents shall be as mentioned below “(1) or (2)” whichever is the later. However, when the site investigator requests a longer storage period, consultation shall be done to determine the storage period and method.

(1) until the study drug provider receives marketing approval for a medical product/device related to the investigational product/device (or when 3 years pass since the development was discontinued or when the study drug provider is notified that the study results will not be attached to the application form)

(2) until 3 years pass since the study was discontinued or completed

The site investigator shall notify the director of the institution when there is a document that no longer requires storage.

6.6.2 Institutional Ethical Committee/IRB

The IRB shall retain the documents specified by the GCP.

The storage period of the documents shall be as mentioned below “(1) or (2)” whichever is later. However, when the site investigator requests a longer storage period, consultation shall be done to determine the storage period and method.

(1) until the study drug provider/medical device provider receives marketing approval for a medical product/device related to the investigational product/device (or when 3 years pass since the development was discontinued or when the study

drug/medical device provider is notified that the study results will not be attached to the application form)

(2) until 3 years pass since the study was discontinued or completed

The site investigator shall notify the IRB when there is a document that no longer requires storage.

6.6.3 Site Investigators

The site investigators shall retain the documents as specified in the GCP. The storage place and period shall be determined in accordance with the “Record Maintenance Procedures Manual” related to the study.

7 Quality Control and Quality Assurance

7.1. Quality Control

7.1.1 Source Document Verification

The monitor shall perform source document verification including verification of other study-related records and CRFs to ensure that the study is safely and appropriately conducted in compliance with the GCP, study protocol, SOP, and relevant laws and regulations, and that the data reliability is ensured.

The director of the institution and site investigator must accept on-site monitoring by the monitor and submit source documents and all other study-related records for source document verification.

In the event of any discrepancies between the source documents and CRFs, the

monitor shall obtain the record that provides the reasons for such discrepancies from the site investigator.

7.1.2 Monitoring

The monitor shall perform on-site monitoring in accordance with the “Monitoring Procedures Manual” related to the study to ensure that the study is appropriately conducted in compliance with the GCP, study protocol, SOP, and relevant laws and regulations, and that the data reliability is secured.

7.1.3 Data Management

The people responsible for and in charge of data management shall perform quality control at each data handling step in accordance with the SOP to ensure the reliability and appropriate handling of all the study-related data.

The people responsible for and in charge of data management shall collect CRFs and perform procedures up to data lock including data check, input, modification, and correction in accordance with the “Data Management Plan”.

7.2 Quality Assurance

7.2.1 Audit

The auditor shall evaluate whether the conduct of this study, data preparation, documentation, and reporting are in compliance with the GCP, study protocol, SOP, and relevant laws and regulations in an independent and separate manner from that conducted during routine monitoring and quality control of the study.

The auditor shall perform audit procedures from an outsider standpoint as part of quality assurance activities in compliance with the “Audit Plan” and “Audit Procedures Manual.”

7.2.2 Source Data/Documents Verification

The director of the institution and site investigator shall cooperate in monitoring, audit, and inspection by the regulatory authorities and IRB by submitting the source documents and all other study-related records for source document verification.

8 Ethics and Responsibility/Conflicts of Interest

8.1 Compliance with the GCP and the Applicable Regulatory Requirement

This study shall be conducted in accordance with the ethical principles set forth in the Declaration of Helsinki (1964) and its revisions, standards provided under Article 14 Paragraph 3 and Article 80-2 of the Pharmaceutical Affairs Law, “Standards for the Implementation of Clinical Trials on Pharmaceutical Products (MHW Ordinance No. 28 dated March 27, 1997), which complied with ICH “Guideline for Good Clinical Practice (GCP)”, its revised ministerial ordinances/operational notifications, SOP, and this study protocol.

8.2 Compliance with the Study Protocol

The site investigators shall submit the study protocol, sample CRF, and informed consent forms to the director of institution to obtain written approval for conducting the study.

The site investigators and study physicians shall conduct the study in compliance with the study protocol.

9 Confidentiality

The parties involved in the study shall give due consideration to the protection of personal information and privacy of participants in compliance with the relevant laws and regulations.

For case registration and the CRF, the participants shall be identified by a participant ID code. In the verification of source documents, informed consent form related to the conduct of the study, and publication of the test results, due consideration shall be given to the protection of the privacy and personal information of the participants such as the name of the participant and disease. CRFs shall not be used for any purposes other than those of this study. In addition, the information obtained through this study shall never be disclosed to third parties.

10 Amendment Policy

When the site investigator judges that the study protocol needs to be revised, and the revision is requested by the IRB or proposed by the “Data and Safety Monitoring Committee”, the site investigator shall prepare a draft revision of the study protocol and provide details on and reasons for the revision to the head of the medical institution and shall obtain approval from the IRB.

See the “Procedures for the Preparation and Revision of the Study Protocol and Sample of Case Report Form” for the revision procedures.

11 Other Support

11.1 Funding Resources and Conflict of Interest

This study shall be conducted by the principal investigator and/or the site investigator subsidized by Otsuka Pharmaceutical Company in cooperation with Otsuka.

The study drug, cilostazol, shall be provided by Otsuka free of charge.

The site investigator and study physician shall voluntarily report possible conflicts of interest affecting this study to the medical institution and take necessary measures as specified by the medical institution.

11.2 Expenses of the Study

In this study, due to the special or specified medical care coverage to be applied, the treatment shall be provided as a health insurance treatment under the special or specified medical care coverage, and the health insurance policy of the participants shall cover the expenses other than those for the administration or injection/use of the study drug and other medical products with equivalent effect or efficacy to the study drug (drugs with the same indications).

11.3 Compensation for Study-related Health Damages

As this study is covered by the compensation insurance provided by an insurance company, the participants shall be compensated for study-related injuries for which a causal relationship with participation in the study cannot be ruled out and judged to be

eligible for the compensation by the above-mentioned insurance.

12 Publication Plan

This study results shall belong to the researchers. The right to use this study results for the purpose of application for marketing approval of medical products/devices shall be discussed as necessary and specified separately.

In terms of publication, study results shall be published as an English article with the head author determined through consultation with the principal investigator and all the other investigators taking part in this study. However, the article shall be jointly published under the names of the principal investigator, all investigators, statistical analyst, and other people determined through consultation with the principal Investigator as the corresponding author.

軽度認知障害患者に対するシロスタゾール療法の
臨床効果ならびに安全性に関する医師主導治験
COMCID（TRINEU1321）

治験実施計画書

治験調整医師（代表）：
国立研究開発法人国立循環器病研究センター病院　脳神経内科　部長　　猪原　匡史
所在地：〒565-8565 大阪府吹田市藤白台 5 丁目 7 番 1 号

2018 年 7 月 2 日　作成（第 1.6 版）

機密保持に関する供述：
本治験実施計画書に含まれる情報は、本試験に直接係る者及び治験審査委員会の委員以外に開示してはならない。また、本情報は事前の書面による治験調整医師の承諾なしに本試験の実施及び評価以外の目的に利用してはならない。

【遵守すべき諸規則】
本治験に関与するすべての者は、世界医師会ヘルシンキ宣言（1964年）及びその改訂版に基づく倫理的原則､及び医薬品、医療機器等の品質、有効性及び安全性の確保等に関する法律第14条第3項及び第80条の2に規定する基準､｢医薬品の臨床試験の実施の基準に関する省令｣（平成9年3月27日　厚生省令第28号）」（＝GCP［Good Clinical Practice の略］）及びその改正省令並びに運用通知、標準業務手順書並びに本治験実施計画書を遵守して実施する。

略語一覧

略語	語形	
Aβ	Amyloid Beta	βアミロイド
AD	Alzheimer’s Disease	アルツハイマー型認知症
ADAS-Cog 14	Alzheimer's Disease Assessment Scale - Cognitive 14	アルツハイマー病評定尺度-認知行動 14
ADAS-Cog-J 14	Alzheimer's Disease Assessment Scale - Cognitive 14 - Japanese	アルツハイマー病評定尺度-認知行動 14 日本語版
ADCS-MCI-ADL	Alzheimer's Disease Cooperative Study - Mild Cognitive Impairment - Activities of Daily Living Inventory	アルツハイマー病共同研究 - MCI - 日常生活動作質問票
ADL	Activities of Daily Living	日常生活動作
IADL	Instrumental Activities of Daily Living	手段的日常生活動作
AE	Adverse Event	有害事象
BMI	Body Mass Index	肥満指数
CDR	Clinical Dementia Rating	臨床認知症評価法
CDR-J	Clinical Dementia Rating-Japanese	臨床認知症評価法 - 日本版
CDR-SB	Clinical Dementia Rating - Sum of Boxes	臨床認知症評価法 - 合計点法 -
DSM-5	Diagnostic and Statistical Manual of mental disorders – 5th edition	精神障害の診断と統計マニュアル-第5版
EDC	Electrical Data Capturing	電子データ収集システム
FCSRT	Free and Cued Selective Reminding Test	自由 - 手掛かり早期検査

GCP	Good Clinical Practice	医薬品の臨床試験の実施の基準に関する省令
MCI	Mild Cognitive Impairment	軽度認知障害
MMSE	Mini - Mental State Examination	精神状態短時間検査
MMSE-J	Mini - Mental State Examination – Japanese	精神状態短時間検査-日本版
MRI	Magnetic Resonance Imaging	核磁気共鳴画像検査
Possible AD Dementia	Possible Alzheimer’s Disease Dementia	アルツハイマー型認知症の疑診
Probable AD Dementia	Probable Alzheimer’s Disease Dementia	アルツハイマー型認知症の確診
SAE	Serious Adverse Event	重篤な有害事象
TMT	Trail Making Test	トレイルメイキングテスト
WMS-R	Wechsler Memory Scale-Revised	ウエクスラー記憶検査法

目 次

概要..1
1 治験の目的 7
1.1 主要目的 7
1.2 副次目的 7
2 背景と根拠 7
3 薬剤情報 9
3.1 治験薬...9
3.2 非臨床試験及び臨床試験（プレタール錠インタビューフォームより） 10
3.3 患者に対する既知及び可能性のある危険と利益の要約 12
4 診断基準と病型分類...14
4.1 MCI（コア臨床基準） 14
4.2 MCI の病型分類..14
5 適格基準...16
5.1 選択基準 16
5.2 除外基準 17
6 説明と同意 18
6.1 説明文書及び同意文書の作成 18
6.2 同意の取得時期と方法 18
6.3 説明文書・同意文書の改訂 18
7 症例登録及び割付 19
7.1 スクリーニング、仮登録...19
7.2 中央判定 19
7.3 割付方法と割付調整因子...19
7.4 割付表の保管と開鍵手続き 19
8 治療計画...20
8.1 治療計画 20
8.2 用量・スケジュール変更基準 21
8.3 併用薬及び併用療法...22
8.4 服薬、その他の取り決め事項の遵守状況の確認 25
8.5 中止基準（治験薬治療を中止する基準） 26
8.6 後治療...27
9 有害事象の評価・報告 27
9.1 重篤及び非重篤有害事象の定義...27
9.2 有害事象の評価及び判定基準 28
9.3 有害事象の報告と発現時の対応...29
9.4 予測される有害事象等 30
10 その他の安全性情報の報告 31
10.1 その他の安全性情報の定義..31

10.2　その他の安全性情報の報告手順 31
11　観察・検査・調査項目とスケジュール 33
11.1　治験期間 33
11.2　観察・検査・調査項目 33
11.3　観察・検査・調査スケジュール 40
12　目標登録症例数と治験実施予定期間 41
12.1　目標登録症例数 41
12.2　治験実施予定期間 41
13　評価項目の定義 41
13.1　主要評価項目 41
13.2　副次評価項目 41
13.3　探索的評価項目 41
13.4　エンドポイントの定義 42
14　統計学的考察 45
14.1　目標登録症例数及びその設定の根拠 45
14.2　解析対象集団 45
14.3　解析項目・方法 45
14.4　中間解析 47
14.5　探索的解析 47
15　治験実施計画書、GCP 及び適用される規制要件の遵守 48
15.1　GCP 等の遵守 48
15.2　治験実施計画書の遵守 48
15.3　治験実施計画書の逸脱又は変更 49
15.4　治験実施計画書の改訂 49
16　治験の終了又は中止 49
16.1　治験の終了 49
16.2　治験の中止 50
17　各種委員会 50
17.1　効果安全性評価委員会 50
17.2　心理検査中央判定委員会 50
17.3　画像診断中央判定委員会 50
18　症例報告書 51
18.1　症例報告書の作成 51
18.2　症例報告書作成上の注意 51
19　治験の品質管理及び品質保証 51
19.1　品質管理 51
19.2　品質保証 53
20　記録の保存 53
20.1　実施医療機関 53
20.2　治験審査委員会 54

20.3 治験責任医師......54
21 治験実施体制及び各種委員会......54
22 治験実施上の倫理的配慮......54
22.1 治験審査委員会......54
22.2 患者の個人情報保護に関する事項......55
23 治験の費用負担及び補償......55
23.1 資金源及び利益相反......55
23.2 治験に関する費用......55
23.3 健康被害に対する補償......56
24 治験の登録、成果の帰属と公表......56
24.1 臨床試験登録......56
24.2 成果の帰属と公表......56
25 参考文献......57

概要

治験課題名	軽度認知障害患者に対するシロスタゾール療法の臨床効果ならびに安全性に関する医師主導治験
治験実施<br>計画書番号	TRINEU1321（2017 年 1 月 16 日　作成　第 1.5 版）
シェーマ	**説明・同意取得**<br>↓<br>**スクリーニング・仮登録**<br>担当医師による適格性の評価（MMSE, CDR を除く）<br>↓<br>**中央評価**<br>心理検査中央判定委員会による MMSE, CDR の評価<br>↓<br>**本登録・ランダム化**<br>**割付因子：年齢・性別・教育歴・施設**<br>**ベースライン評価**<br>↓<br>**被験薬群**<br>シロスタゾール錠 50mg<br>1 回 1 錠<br>1 日 2 回経口投与<br>96 週継続<br>**対照薬群**<br>プラセボ錠<br>1 回 1 錠<br>1 日 2 回経口投与<br>96 週継続<br>↓<br>**効果判定**<br>主要評価項目：<br>MMSE スコアの変化量（ベースライン値と治療開始後第 96 週のスコア差)<br>副次評価項目：<br>MCI から「あらゆる原因による認知症」への移行までの時間<br>CDR-SB の変化量<br>ADAS-Cog 14 の変化量<br>WMS-R Logical Memory の変化量<br>ADCS-MCI-ADL の変化量<br>MRI にて測定される海馬容積の変化量

目的	主要目的<br>MCI 患者に対するシロスタゾール投与の臨床効果と安全性について、MMSE を用いて評価する。<br>副次目的<br>1） MCI 患者において、シロスタゾール投与による MCI から認知症への移行までの期間の延長効果を評価する。<br>2） MCI 患者において、シロスタゾール投与による CDR-SB の変化量を評価する。<br>3） MCI 患者において、シロスタゾール投与による ADAS-Cog 14 の変化量を評価する。<br>4） MCI 患者において、シロスタゾール投与による WMS-R Logical Memory の変化量を評価する。<br>5） MCI 患者において、シロスタゾール投与の臨床効果について、ADCS-MCI-ADL を用いて、日常生活動作（ADL）への影響について評価する。<br>6） MCI 患者において、シロスタゾール投与による海馬萎縮の進行の抑制効果を MRI にて評価する。<br>上記の目的を踏まえ、本治験のリサーチ・クエスチョンを以下のように定義する。<br>1） シロスタゾールはプラセボと比較して、MCI から認知症への移行を抑制する。<br>2） シロスタゾールはプラセボと比較して、認知障害の進行を抑制する。<br>3） シロスタゾールはプラセボと比較して、MCI 患者の日常生活動作（ADL）の低下を抑制する。<br>4） シロスタゾールはプラセボと比較して、海馬萎縮の進行を抑制する。
適格基準	選択基準<br>1） 同意取得時年齢が 55 歳以上 84 歳以下の患者<br>2） 原則として在宅で生活し、スタディパートナー[注1)]を伴っていること<br>3） NIA-AA 診断基準により MCI と診断され、下記の客観的証拠を満たす患者<br>1. 本人またはスタディパートナーから記憶障害の訴えがある[注2)]<br>① タイプ I : 本人からの記憶障害の訴え ＋ スタディパートナーの立場の者がそれを容認する。<br>② タイプ II : 本人からの記憶障害の訴えはなくても、スタディパートナーから記憶障害の事実が示される。<br>2. MMSE スコアは 22 点以上 28 点以下（心理検査中央判定委員会による評価）<br>3. CDR は 0.5（心理検査中央判定委員会による評価）<br>4） 本研究への参加について書面によるインフォームドコンセントが得ら

	れている<br>注1)スタディパートナーとは患者をよく知り、患者に関して本試験への情報提供を行う者であり、<br>①　日常生活を自立して行えること<br>②　記憶障害の訴えが無く、軽度認知障害・認知症を有しないこと<br>③　本登録日より 3 年前から患者との接触があり、現在は週 3 日以上、計 2 時間以上の患者との接触があること。(必ず患者の自宅内での接触を含むこと)<br>④　治験参加期間中すべての診察に同行できること<br>が条件である。<br>また、スタディパートナーの治験期間中の病気等の理由による変更は可能とする。ただし、スタディパートナーが上述の条件を満たすことが不可能と認めた時点から 12 週間以内に、医師又は CRC 等まで申し出なければならない。申し出があった場合は、患者及び変更後のスタディパートナーの再同意を得る必要がある。代わりのスタディパートナーがいない場合はその時点で中止とする。<br>注2)本人からの記憶障害のみで、スタディパートナーがそれを容認しない場合は除外する。<br>【設定の根拠】<br>1) － 3) : 臨床効果を適切に評価するため<br>4) : GCP 遵守のため<br>除外基準<br>1)　パーキンソン病、ハンチントン病、正常圧水頭症、進行性核上性麻痺、てんかん、多発性硬化症、頭蓋内感染症、後遺症を残した頭部外傷がある場合。<br>2)　仮登録前 48 週以内に実施した脳 MRI 検査により、多発脳梗塞、脳腫瘍、硬膜下血腫が認められた場合。<br>3)　ペースメーカー、動脈瘤クリップ、人工弁、人工内耳その他磁性体・電気伝導性の金属が入っており、脳 MRI 撮像時に問題が生じる場合。<br>4)　仮登録前 48 週以内に大うつ病や双極性障害、仮登録前 96 週以内にアルコールや他の薬物依存の既往がある場合、重篤な疾患や状態の安定しない疾患に罹患している場合。<br>5)　HbA1c の値が 9.0%を超えている場合。<br>6)　ビタミン B12・葉酸欠乏による認知機能障害の場合。<br>7)　神経梅毒の場合。<br>8)　甲状腺機能異常による認知機能障害の場合。<br>9)　向精神薬（抗うつ剤、抗精神病薬など）を仮登録前 4 週以内に服用している場合。<br>10）抗凝固薬（ワルファリンカリウム、ダビガトラン等）を仮登録前 4 週

	以内に服用している場合。<br>11）二剤以上の抗血小板薬（アスピリン製剤、硫酸クロピドグレル等）を仮登録前 4 週以内に服用している場合。（シロスタゾールの場合は一剤の時点で不可とする。）<br>12）重度の糖尿病を有し、インスリンを仮登録前 4 週以内に投与している場合。<br>13）意識障害を伴う低血糖発作が仮登録前 4 週以内に 1 回以上起きている場合。<br>14）抗認知症薬（ドネペジル、抑肝散等）を仮登録前 4 週以内に服用している場合。<br>15）新規アルツハイマー型認知症治療薬の治験に参加しているまたは参加を予定しており、本治験と両方に参加する可能性がある場合。<br>16）出血している、もしくは出血の危険性を有する患者（血友病、毛細血管脆弱症、頭蓋内出血、消化管出血、尿路出血、喀血、硝子体出血、過多月経、大動脈瘤、脳動脈瘤等）。<br>17）うっ血性心不全の場合。<br>18）冠動脈狭窄の場合。<br>19）持続して血圧が上昇している場合（収縮期血圧：160 mmHg、拡張期血圧：100 mmHg を超えた状態が仮登録前 2 週間継続している患者）<br>20）本剤の成分に対し過敏症の既往歴がある場合。<br>21）被験者又は被験者のパートナーが仮登録時点で妊娠中及び授乳中の場合。<br>22）聴力障害や視力障害により、本治験に必須の神経心理検査を遂行することが困難な場合。<br>23）その他、治験責任医師が不適格と認定した場合。（治験分担医師を含む。）<br>【設定の根拠】<br>1）－ 9)：臨床効果の評価に影響を及ぼすことが考えられるため<br>10）－ 11)：安全性上の配慮<br>12）－ 15)：臨床効果の評価に影響を及ぼすことが考えられるため<br>16）－ 21)：安全性上の配慮<br>22)：臨床効果の評価に影響を及ぼすことが考えられるため
目標症例数	200 例（被験薬群 100 例、対照薬群 100 例）
治験実施<br>予定期間	症例登録期間　：2015 年 5 月から 2018 年 3 月（終了予定）（35 か月間）<br>（ただし、登録期限前に目標症例数の 200 例に達した場合等は、その時点で終了とする）<br>治験期間　：2015 年 5 月から 2020 年 3 月（終了予定）<br>（最終症例本登録日から 2 年後）
治験の	開発の相　：第 II 相

デザイン	治験のデザイン　：並行群間比較<br>対照の種類　　　：同時並行プラセボ対照<br>ランダム化　　　：有<br>盲検化のレベル　：二重盲検
治験薬、用量及び投与方法	被験薬：シロスタゾール錠 50 mg<br>対照薬：プラセボ錠<br><br>いずれかの薬剤である治験薬を 1 回 1 錠　1 日 2 回経口投与を 96 週継続する。なお、シロスタゾール錠（被験薬）とプラセボ錠（対照薬）は外観上区別されない錠剤である。
【有効性評価】	
主要評価項目	MMSE（邦訳として MMSE-J を使用）スコアの変化量(スクリーニング時と治療開始後第 96 週測定時の差分)
副次評価項目	MCI から「あらゆる原因による認知症」への移行までの時間<br>CDR-SB（邦訳として CDR-J を使用）の変化量<br>ADAS-Cog 14（邦訳として ADAS-Cog-J 14 を使用）の変化量<br>WMS-R Logical Memory（日本版ウエクスラー記憶検査法　論理的記憶 I と論理的記憶 II を使用）の変化量<br>ADCS-MCI-ADL を用いた ADL（18 項目：0－53 点）の変化量<br>MRI にて測定される海馬容積の変化量
安全性評価	有害事象の発現の有無
【統計手法】	
解析対象集団	本治験の主要評価項目及び副次評価項目などの臨床効果に関しては、最大の解析対象集団（FAS）を解析対象集団とする。有害事象やその他の観察項目については、安全性解析対象集団を対象とする。
主要評価項目に関する解析	<u>MMSE スコアの変化量</u><br>各被験者のスクリーニング時の MMSE スコアと治療開始後第 24、48、72、96 週スコアの差分について、その平均値を被験薬群と対照薬群間で比較検定する。ただし、第 96 週における差分スコアの群間平均値の有意差をもって本治験の有効性の確認とする。この確認のため、治療群、性別及び時期を固定効果、被験者 ID×時期を変量効果とした混合モデル反復測定(mixed model repeated mesasures: MMRM)を用いる。
副次評価項目に関する解析	① <u>MCI から「あらゆる原因による認知症」への移行までの時間</u><br>それぞれの期間に関して群別に Kaplan-Meier 法を適用し、生存関数を推定する。また、それらの推定生存曲線を図示する。さらに、ログランク検定を適用し、それぞれの生存期間を群間比較する。さらに被験者背景因子を含めた多変量 Cox 比例ハザードモデルを用いた解析を行う。<br>② <u>神経心理検査のスコア（CDR-SB、ADAS-Cog 14、WMS-R Logical Memory）の変化量</u>

	各神経心理検査のスコアについて、MMSE と同様に解析する。CDR については CDR-SB を算出し解析する。<br>③ ADCS-MCI-ADL の変化量<br>ADL を 18 項目（0－53 点）で評価し、MMSE と同様に解析する。<br>④ MRI にて測定される海馬容積の変化量<br>FreeSurfer を用いて、自動セグメンテーションの手法で海馬領域の自動ラベリングを行い、左右の海馬容積を加算した総海馬容積を算出する。その上で、ベースライン時および治験開始後第 96 週後の総海馬容積に基づき年間萎縮率を算出し、海馬萎縮の進行の程度とする。この値を被験薬群と対照薬群間で比較検定する。
中間解析	実施しない。
連絡先	治験調整事務局：<br>国立研究開発法人　国立循環器病研究センター病院　脳神経内科<br>所在地：〒565-8565 大阪府吹田市藤白台 5 丁目 7 番 1 号<br>TEL : 06-6833-5012(代)　内線：2686<br>FAX : 06-6872-8090<br>E-mail：r_kakuta@ncvc.go.jp<br>担当者：　角田　良介

1　治験の目的

1.1　主要目的

軽度認知障害（MCI）患者に対するシロスタゾール投与の臨床効果と安全性について、ミニメンタルステート検査（MMSE）スコアを用いて評価する。

1.2　副次目的

1）MCI 患者において、シロスタゾール投与による MCI から認知症への移行までの期間の延長効果を評価する。
2）MCI 患者において、シロスタゾール投与による CDR-SB の変化量を評価する。
3）MCI 患者において、シロスタゾール投与による ADAS-Cog 14 の変化量を評価する。
4）MCI 患者において、シロスタゾール投与による WMS-R Logical Memory の変化量を評価する。
5）MCI 患者において、シロスタゾール投与の臨床効果について、ADCS-MCI-ADL を用いて、日常生活動作（ADL）への影響について評価する。
6）MCI 患者において、シロスタゾール投与による海馬萎縮の進行の抑制効果を MRI にて評価する。

上記の目的を踏まえ、本治験のリサーチ・クエスチョンを以下のように定義する。

1）シロスタゾールはプラセボと比較して、MCI から認知症への移行を抑制する。
2）シロスタゾールはプラセボと比較して、認知障害の進行を抑制する。
3）シロスタゾールはプラセボと比較して、MCI 患者の日常生活動作（ADL）の低下を抑制する。
4）シロスタゾールはプラセボと比較して、海馬萎縮の進行を抑制する。

2　背景と根拠

軽度認知障害（MCI）は、アルツハイマー型認知症（AD Dementia: Alzheimer's disease Dementia）等の認知症とは言えないが、知的に正常ともいえない状態を指す[1]。認知症、MCI はともに超高齢社会となった日本において非常に重要な疾患となっている。加齢とともに認知症の発症率は増加するが、高齢者の認知症有病率は 15%[2]、発症率は十～数十人/1000 人年と言われ[3,4]、非常に頻度が高く対応に迫られている。しかし、軽度認知障害（MCI）からアルツハイマー型認知症（AD Dementia: Alzheimer's disease Dementia）への移行を防ぐ確実な治療方法は現時点では確立されていない。これまでのアルツハイマー型認知症の治療は、β/γセクレターゼ阻害薬によるβアミロイド（Aβ）の産生低下や Aβワクチン療法による Aβ可溶化を目指して行われてきたが、孤発性のアルツハイマー型認知症ではむしろ Aβのクリアランスの低下が主に関与していると考えられており[5]、シロスタゾールによる Aβ排泄促進により、これまでとは異なる観点からのアルツハイマー型認知症の発症の遅延効果が期待される。

げっ歯類の前臨床試験では、脳循環を低下させることにより、アルツハイマー型認知

症の認知機能が加速度的に悪化すること[6]、Aβの蓄積が増加すること[7]が示されており、逆にホスホジエステラーゼIII阻害薬のシロスタゾールを投与すると、血管拡張予備能や脳血流予備能が改善してAβ沈着を予防し、認知機能が改善することが示されている[8](特願2012－135906、出願日2012年6月15日)。またNun研究では、1－2 個の脳梗塞の存在が、生前のアルツハイマー型認知症の顕性化率を20倍に高めるとの報告があり[9]、血管作動性の薬剤により、Aβの排泄促進を高め、かつ脳梗塞を予防することで、MCIからアルツハイマー型認知症への移行を遅延させることができると考えられる。

一方、順天堂大学の新井らは、アルツハイマー型認知症患者10 例に対しドネペジル治療にシロスタゾール100mg/日の追加投与を行い、10 例中8 例においてベースラインから比べて5－6か月後のミニメンタルステート検査（MMSE）スコアの低下が抑制されたと報告した（平均追跡期間7.4か月）[10]。

また、田口らは、1996 年から 2012 年の間にある地域の病院における連続症例 3183例のレトロスペクティブ解析により、シロスタゾールを処方され 6 か月以上の間隔で 2回以上 MMSE による評価を受けたすべての患者を抽出するためカルテ記録を調査した。なお、抗認知症薬を処方された症例は除外された。シロスタゾールが投与された患者のMMSEスコアの時間的変化と、シロスタゾール投与をやめた患者のスコア変化とが比較された。平均追跡期間は 691 日だった。この調査により、シロスタゾールの投与によりMMSEスコアの減少が有意に抑制されることが発見された（p値＜0.05)。サブグループ解析により、シロスタゾール中止患者においては MMSE スコアの変化が－3.8 ± 1.3 /yearであったのに対し、シロスタゾール投与患者においてはMMSEスコアの変化が＋0.4 ± 0.7 /yearであった[11]。

さらに猪原らは、ドネペジルを投与されシロスタゾールを投与された（6か月以上投与された）もしくは投与されなかった患者で、12か月以上の間隔で2回以上MMSEによる評価を受けたすべての患者を同じカルテ記録から抽出した。この調査により、ドネペジルのみで治療された患者87例のMMSEスコアの時間的変化と、ドネペジルとシロスタゾールで治療された患者69例のスコア変化とが比較された。この調査により、シロスタゾールの同時投与ではMMSEスコアの減少は有意には抑制されなかった。しかし、軽度認知症（MMSEスコアが22以上26以下）患者のサブグループ解析により、ドネペジル単独で治療された患者ではMMSEスコアの大きな減少が観測されたが(N=36; －2.23 ± 0.69/year)、シロスタゾールにより MMSE スコアの変化が抑制されることが示された(N=34; －0.45 ± 0.28/year)[12]。

加えて、日本の脳梗塞（心原性脳塞栓症を除く）発症後の再発抑制に対する用量・用法は1回100 mg　1日2回である。この事実はシロスタゾール1回100 mg　1日2回以下の長期投与が安全であることを支持している。

そこで、軽度認知障害（MCI）患者において、シロスタゾール投与によるミニメンタルステート検査（MMSE）スコアを用いた認知機能への効果を評価する医師主導治験を計画することとなった。

3　薬剤情報

3.1　治験薬

3.1.1　治験薬

被験薬:シロスタゾール錠 50 mg（1 回 1 錠　1 日 2 回経口投与　96 週継続）

対照薬：プラセボ錠　（1 回 1 錠　1 日 2 回経口投与　96 週継続）

シロスタゾール錠（被験薬）とプラセボ錠（対照薬）は外観上区別されない錠剤である。

3.1.2　シロスタゾール錠（被験薬）

概要

A). 被験薬

被験薬：プレタール錠 50 mg

一般名：シロスタゾール

化学名：

6-[4-(1-Cyclohexyl-1H-tetrazol-5-yl)butyloxy]-3,4-dihydroquinolin-2(1H)-one(IUPAC)

シロスタゾールは、分子量 369.46 の物質である。本品はメタノール、エタノール（99.5）又はアセトニトリルに溶けにくく、水にほとんど溶けない。本剤の治療学的・製剤学的特性は下記の通りである。

① 血小板凝集を抑制し、抗血栓作用を示す。
② 血管拡張作用を有し、脳梗塞慢性期患者の血流量を増加させる。
③ 下肢血流量を増加させ、末梢の血行動態を改善する。
④ 二重盲験比較試験において脳梗塞（心原性脳塞栓症を除く）発症後の再発を抑制することが確認されている。
⑤ 慢性動脈閉塞症に基づく潰瘍、疼痛、冷感等の虚血性諸症状を改善する。

B). 毒性

治験薬概要書を参照。

C). 薬物動態

治験薬概要書を参照。

D). 被験薬の成分・含量・剤型

1（錠）中シロスタゾール 50 mg 含有する。

E). 治験薬の表示・包装

別途定めた当該治験に係る「治験薬の管理に関する手順書」に記載する。

F). 治験薬の貯法

室温保存（貯蔵条件；室温、遮光、湿度等）

G). 治験薬の提供・保管・管理・回収

治験責任医師は、治験薬提供者から治験薬の提供を受ける。提供された治験薬、残余治験薬、使用済み包装箱等、治験薬の出納について、治験薬管理者が管理する。これらの手順については、当該治験に係る「治験薬の管理に関する手順書」に従う。

なお、本治験薬は本治験にのみ使用し、他の目的に使用してはならない。

H). 治験薬の処方

治験責任医師又は治験分担医師は治験スケジュールに従い、治験薬を処方する。その際、未使用の治験薬の返却等、治験薬の取り扱いについて被験者に十分に説明する。なお、被験者に配布した薬剤の PTP 包装は回収する。

I). 治験薬提供者

大塚製薬株式会社

3.1.3 プラセボ錠（対照薬）

概要

A). 対照薬

プラセボ錠（対照薬）は前記のシロスタゾール錠（被験薬）と外観上区別されず、特定の薬理作用を有しない錠剤である。

3.2 非臨床試験及び臨床試験（プレタール錠インタビューフォームより）

(1). 臨床効果

プレタール錠の成績を以下に示す。

① 慢性動脈閉塞症に基づく潰瘍、疼痛及び冷感等の虚血性諸症状の改善

慢性動脈閉塞症患者 226 例について実施された二重盲検比較試験を含む臨床試験において、四肢の末梢血流障害による潰瘍、疼痛及び冷感等の虚血性症状に対する全般改善度は、改善以上 66.1%(119/180 例)、やや改善以上 85.0%(153/180 例)であった。

三島好雄ほか：臨床評価, 14(1), 13－41, 1986

② 脳梗塞（心原性脳塞栓症を除く）発症後の再発抑制

脳梗塞患者 1,034 例について実施されたプラセボを対照とする二重盲検比較試験において、脳梗塞の年間再発率はプラセボ 5.75%（総観察期間［人×年］: 973.7、脳梗塞再発例数：56）に対し、本剤 3.43%（総観察期間［人×年］: 873.8、脳梗塞再発例数：30）であり、本剤は脳梗塞再発のリスクを 40.3%軽減させた。なお、二次評価項目である投薬期間における「理由を問わない死亡」では、本剤群及びプラセボ群の年間死亡率推定値は、それぞれ 0.92%及び 0.82%であり、年間死亡率の推定値に有意差は認められなかった。また、本試験において投薬期間中に狭心症を発症した症例は、プラセボ群（0/518 例）に対し本剤群（6/516 例）で多く認められた。

前川達郎ほか：社内資料(CSPS), 2003

Shinohara, Y. et al.：Lancet Neurol., 9, 959-968, 2010

(2). 臨床薬理試験:忍容性試験

健康成人男子を対象に、25－300 mg の用量でシロスタゾールの単回投与を行った。その結果、75 mg 以上の用量で頭痛又は頭重感が認められたが、臨床検査所見、血圧、心拍数に異常は認められなかった。健康成人男子を対象に、50 mg、100 mg、150 mg の用量でシロスタゾールの単回投与を行った後、50 mg、100 mg の 1 日 1 回、4 日間連続投与、50 mg、100 mg の 1 日 2 回 4 日間連続投与、100 mg 1 日 3 回 4 日間連続投与を行った。その結果、50 mg 単回投与群では自覚症状の出現はみられなかったが、その他の群では頭痛・頭重感、体温の異常上昇を伴わない発熱感、消化器症状の訴え等がみられた。これらの症状はいずれも重篤なものではなく、また他覚症状、臨床検査においても特別な異常変化は認められなかった。

Niki, T. et al.：Arzneim.-Forsch./Drug Res., 35(Ⅱ), 1173－1185, 1985

安部 英ほか：臨床評価, 13(1), 257－280, 1985

注意： 本剤の承認されている用法・用量は、「通常、成人には、シロスタゾールとして 1 回 100 mg を 1 日 2 回経口投与する。なお、年齢、症状により適宜増減する。」となっている。

(3). 探索的試験:用量反応探索試験

虚血性潰瘍を有する四肢慢性動脈閉塞症に対してシロスタゾールを 1 日量として 100 mg、200 mg、300 mg を 6 週間経口投与した結果、有効性については 1 日量として 200 mg、300 mg がほぼ同等の改善率を示し、安全性については 300 mg でやや劣る成績であった。有用性については 200 mg で最も高い有用率を示した。

内田 發三ほか：循環器科, 17(4), 421－432, 1985

注意： 本剤の承認されている用法・用量は、「通常、成人には、シロスタゾールとして 1 回 100 mg を 1 日 2 回経口投与する。なお、年齢、症状により適宜増減する。」となっている。

3.3　被験者に対する既知及び可能性のある危険と利益の要約

1）予測される利益

本治験から、被験者は明確な利益を得られるとは限らない。被験者の症状は、回復、不変、もしくは悪化する可能性がある。

2）予測される不利益

治験薬の投与に伴う副作用は、常に潜在的な懸念事項である。また、治験に参加した場合、来院し事前検査、定期検査、服薬中止後の追跡調査などに協力する必要があり、時間的に拘束されることがある。

また、これまで受けていた治療が本治験の併用禁止薬に該当する場合、その治療を中止しなければいけない。

シロスタゾールの副作用については下記の通りである。

(1). シロスタゾールの副作用

調査症例 4890 例中 436 例（8.92%）に臨床検査値の異常を含む副作用が認められた。また以下の副作用が発現することがある。

(2). シロスタゾールの重大な副作用

・ うっ血性心不全、心筋梗塞、狭心症、心室頻拍
・ 出血（脳出血等の頭蓋内出血、肺出血、消化管出血、鼻出血、眼底出血等）
・ 胃・十二指腸潰瘍
・ 汎血球減少、無顆粒球症、血小板減少
・ 間質性肺炎（発熱、咳嗽、呼吸困難、胸部 X 線異常、好酸球増多を伴う）
・ 肝機能障害（AST（GOT）、ALT（GPT）、Al-P、LDH 等の上昇）、黄疸
・ 急性腎不全

(3). シロスタゾールのその他の副作用

シロスタゾールのその他の副作用を表 2 に示す。

表 2 シロスタゾール（プレタール錠 50 mg / 100 mg）のその他の副作用

種類/頻度	5%以上	0.1～5%未満	0.1%未満	頻度不明
過敏症		発疹、皮疹、そう痒感	蕁麻疹等	光線過敏症、紅斑
循環器		動悸、頻脈、ほてり、血圧上昇、血圧低下、心房細動・上室性頻拍・上室性期外収縮・心室性期外収縮等の不整脈等		
精神神経系	頭痛、頭重感	めまい、不眠、しびれ感	眠気、振戦、肩こり、失神・一過性の意識消失等	
消化器		腹痛、悪心・嘔吐、食欲不振、下痢、胸やけ、腹部膨満感、味覚異常	口渇等	
血液		貧血、白血球減少	好酸球増多等	
出血傾向		皮下出血、血尿等		
肝臓		AST(GOT)、ALT(GPT)、Al-P、LDHの上昇等		
腎臓		BUN上昇、クレアチニン上昇、尿酸値上昇、頻尿	排尿障害等	
その他		発汗、浮腫、胸痛、血糖上昇、耳鳴、倦怠感、結膜炎、発熱、脱毛	疼痛、筋痛、脱力感	

4　診断基準と病型分類

4.1　MCI（コア臨床基準）

以下の基準に適合する場合に MCI と診断する[13]。なお、DSM-5 基準では Mild Neurocognitive Disorder にほぼ該当する。

- 被験者本人または情報提供者もしくは臨床医から、認知機能の変化を反映した認知面の問題（すなわち、経時的な機能低下の病歴または観察所見）が報告されている。
- 1 つまたは複数の認知機能領域（典型的には記憶を含む）について、認知障害の客観的な証拠（すなわち、複数領域における認知機能の水準を確認するための正式な検査またはベッドサイド検査での異常所見）がある。
- 機能面で自立が保たれている。
- 認知症ではない。

ただし、診断時には以下の条件について検討すること。

- 可能な限り、外傷、その他の医学的原因による認知機能の低下を除外する。
- 可能な限り、認知機能の経時的低下を示す証拠を提示する。
- 可能な限り、アルツハイマー型認知症の遺伝学的因子と整合する病歴があるかどうか確認する。

4.2　MCI の病型分類

4.2.1　健忘型 MCI（Amnestic MCI）

WMS-R, Logical Memory のうち 1 パラグラフの遅延再生検査（カットオフ値は教育年数別に設定[注1)]）において記憶障害と判定された被験者は、「健忘型 MCI」に分類する[14,15]。

注 1）　教育年数が 0－7 年の場合のカットオフ値は 2 点以下、8－15 年は 4 点以下、16 年以上は 8 点以下とする。

また、健忘型 MCI は単一領域と複数領域に分類する。

1）　単一領域の健忘型 MCI（Amnestic MCI single domain）

記憶障害のみが認められた被験者は、「単一領域の健忘型 MCI」に分類する。

2）　複数領域の健忘型 MCI（Amnestic MCI multiple domain）

神経診察にて別の領域にも障害が認められた被験者は、「複数領域の健忘型 MCI」に分類する。

4.2.2 非健忘型 MCI（Non-amnestic MCI）

「健忘型 MCI」の基準に適合しない被験者は、「非健忘型 MCI」に分類する[14,15]。

また、非健忘型 MCI も同様に単一領域と複数領域に分類する。

1） 単一領域の非健忘型 MCI（Non-amnestic MCI single domain）

神経診察にて単一の領域（ただし、記憶障害を除く）に障害が認められた被験者は、「単一領域の非健忘型 MCI」に分類する。

2） 複数領域の非健忘型 MCI（Non-amnestic MCI multiple domain）

神経診察にて複数の領域（ただし、記憶障害を除く）に障害が認められた被験者は、「複数領域の非健忘型 MCI」に分類する。

5 適格基準

5.1 選択基準

1） 同意取得時年齢が 55 歳以上 84 歳以下の患者

2） 原則として在宅で生活し、スタディパートナー注 1)を伴っていること

注 1) スタディパートナーとは、患者をよく知り、患者に関して本試験への情報提供を行う者であり、

① 日常生活を自立して行えること

② 記憶障害の訴えが無く、軽度認知障害・認知症を有しないこと

③ 本登録日より 3 年前から接触があり、現在は週 3 日以上、計 2 時間以上の患者との接触があること。（必ず患者の自宅内での接触を含むこと）

④ 治験参加期間中すべての診察に同行できること

が条件である。

また、スタディパートナーの治験期間中の病気等の理由による変更は可能とする。ただし、スタディパートナーが上述の条件を満たすことが不可能と認めた時点から 12 週間以内に、医師又は CRC 等まで申し出なければならない。申し出があった場合は、患者及び変更後のスタディパートナーの再同意を得る必要がある。代わりのスタディパートナーがいない場合はその時点で中止とする。

3） NIA-AA 診断基準により MCI と診断され、下記の客観的証拠を満たす患者

1. 本人またはスタディパートナーから記憶障害の訴えがある注 2)
   a). タイプ I : 本人からの記憶障害の訴え ＋ スタディパートナーの立場の者がそれを容認する。
   b). タイプ II : 本人からの記憶障害の訴えはなくても、スタディパートナーから記憶障害の事実が示される。
2. MMSE スコアは 22 点以上 28 点以下（心理検査中央判定委員会による評価）
3. CDR は 0.5（心理検査中央判定委員会による評価）

注 2) 本人からの記憶障害のみで、スタディパートナーがそれを容認しない場合は除外する。

4） 本研究への参加について書面によるインフォームドコンセントが得られている。

【設定の根拠】

1） － 3）: 臨床効果を適切に評価するため

4）: GCP 遵守のため

5.2 除外基準

1） パーキンソン病、ハンチントン病、正常圧水頭症、進行性核上性麻痺、てんかん、多発性硬化症、頭蓋内感染症、後遺症を残した頭部外傷がある場合。
2） 仮登録前 48 週以内に実施した脳 MRI 検査により、多発脳梗塞、脳腫瘍、硬膜下血腫が認められた場合。
3） ペースメーカー、動脈瘤クリップ、人工弁、人工内耳その他磁性体・電気伝導性の金属が入っており、脳 MRI 撮像時に問題が生じる場合。
4） 仮登録前 48 週以内に大うつ病や双極性障害、仮登録前 96 週以内にアルコールや他の薬物依存の既往がある場合、重篤な疾患や状態の安定しない疾患に罹患している場合。
5） HbA1c の値が 9.0%を超えている場合。
6） ビタミン B12・葉酸欠乏による認知機能障害の場合。
7） 神経梅毒の場合。
8） 甲状腺機能異常による認知機能障害の場合。
9） 向精神薬（抗うつ剤、抗精神病薬等）を仮登録前 4 週以内に服用している場合。
10） 抗凝固薬（ワルファリンカリウム、ダビガトラン等）を仮登録前 4 週以内に服用している場合。
11） 二剤以上の抗血小板薬（アスピリン製剤、硫酸クロピドグレル等）を仮登録前 4 週以内に服用している場合。（シロスタゾールの場合は、一剤の時点で不可とする。）
12） 重度の糖尿病を有し、インスリンを仮登録前 4 週以内に投与している場合。
13） 意識障害を伴う低血糖発作が仮登録前 4 週以内に 1 回以上起きている場合。
14） 抗認知症薬（ドネペジル、抑肝散等）を仮登録前 4 週以内に服用している場合。
15） 新規アルツハイマー型認知症治療薬の治験に参加しているまたは参加を予定しており、本治験と両方に参加する可能性がある場合。
16） 出血している、もしくは出血の危険性を有する場合（血友病、毛細血管脆弱症、頭蓋内出血、消化管出血、尿路出血、喀血、硝子体出血、過多月経、大動脈瘤、脳動脈瘤等）。
17） うっ血性心不全の場合。
18） 冠動脈狭窄の場合。
19） 持続して血圧が上昇している場合（収縮期血圧 : 160 mmHg、拡張期血圧 : 100 mmHg を超えた状態が仮登録前 2 週間継続している患者）。
20） 本剤の成分に対し過敏症の既往歴がある場合。
21） 被験者又は被験者のパートナーが仮登録時点で妊娠中及び授乳中の場合。
22） 聴力障害や視力障害により、本治験に必須の神経心理検査を遂行することが困難な場合。
23） その他、治験責任医師が不適格と認定した場合。（治験分担医師を含む。）

【設定の根拠】

1）－ 9)：臨床効果の評価に影響を及ぼすことが考えられるため
10）－ 11)：安全性上の配慮
12）－ 15)：臨床効果の評価に影響を及ぼすことが考えられるため
16）－ 21)：安全性上の配慮
22)：臨床効果の評価に影響を及ぼすことが考えられるため

6 説明と同意

6.1 説明文書及び同意文書の作成

治験責任医師は、説明文書及び同意文書を作成し、あらかじめ治験審査委員会で承認を得る。説明文書に記載すべき項目については、「医薬品の臨床試験の実施の基準に関する省令」第51条及びその運用通知に基づき、作成する。

6.2 同意の取得時期と方法

治験責任医師又は治験分担医師は、被験者が治験に参加する前に、説明文書を用いて十分説明し、被験者とスタディパートナーが内容をよく理解したことを確認した上で、治験への参加について自由意思による同意を被験者本人とスタディパートナーの両者から文書で取得する。

同意文書には、説明を行った治験責任医師又は治験分担医師、被験者、並びにスタディパートナーが説明文書の内容を十分に理解した上で、治験に参加することに同意する旨を記載した同意文書に、記名押印又は署名し、各自日付を記入する。なお、治験協力者が補足的に説明を行った場合には、当該治験協力者も記名押印又は署名し、日付を記入する。

治験責任医師又は治験分担医師は、記名押印又は署名した同意文書の写しを説明文書と共に被験者とスタディパートナーに交付し、同意文書原本はカルテとともに当該実施医療機関で保存する。

6.3 説明文書・同意文書の改訂

治験への参加の継続について被験者の意思に影響を及ぼすと考えられる新たな情報が得られた場合、治験責任医師又は治験分担医師は、速やかに被験者とスタディパートナーに伝え、治験への参加の継続について被験者とスタディパートナーの意思を確認し、記録に残す。

治験責任医師は、速やかに当該情報に基づき説明文書を改訂し､治験審査委員会・実施医療機関の長の承認を得る。

治験責任医師又は治験分担医師は、改訂した説明文書を用いて被験者とスタディパートナーに十分に説明し、治験への参加の継続について被験者とスタディパートナーの両者の意思を再度確認するとともに、文書による同意を取得する。

7 症例登録及び割付

7.1 スクリーニング・仮登録

実施医療機関の治験責任医師又は治験分担医師は、選択基準および除外基準に基づき、治験を実施するための被験者の適格性について確認し、適格性を有する可能性がある場合は、当該治験責任医師又は治験分担医師が実施した評価内容を登録システムに入力する（仮登録）。また、仮登録した症例の MMSE および CDR の検査紙を心理検査中央判定委員会に提出し、本登録可否の判定を依頼する。

7.2 中央判定・本登録

心理検査中央判定委員会は、治験責任医師又は治験分担医師から提出された MMSE および CDR の評価判定を行い、本登録の可否を判定する。心理検査中央判定委員会は、登録システムに最終判定した結果を入力し、本登録とする。ただし、心理検査中央判定委員会にて本治験の適格基準外と判定された場合は、本登録に至らない。治験責任医師又は治験分担医師は登録システムを通し判定結果を確認する。

7.3 割付方法と割付調整因子

各被験者には、被験薬あるいは対照薬のいずれかがランダムに割付けられる。

割付方法は最小化法とし、割付調整因子は年齢（75 歳未満 vs. 75 歳以上）、性別、教育歴（12 年以下 vs. 12 年を超える）及び施設とする。割付の詳細は「割付手順書」にて定める。

7.4 割付表の保管と開鍵手続き

7.4.1 割付表の保管

薬剤割付表と被験者割付情報は、開鍵時まで割付担当者が保管する。

割付責任者は薬剤割付表・緊急用キーコード表を開鍵時まで保管する。

7.4.2 開鍵手続き

1） 通常の開鍵

すべてのデータが固定された後、データセンターは、割付責任者に割付表の開示を依頼する。割付責任者はデータベースの固定を確認し、割付担当者に被験者割付情報の開示を依頼し、該当症例の被験者割付情報をデータセンターに送付する。

2） 緊急時の開鍵

治験責任医師は、重篤な有害事象発現等により被験者の安全性確保のため割付内容を知る必要があると判断した場合、治験調整委員会に緊急用キーコードの開示を依頼する。治験調整委員会は緊急用キーコードの開示の可否を判断し、割付責任者に緊急キーコードの開示を依頼する。割付責任者は、依頼を受けてから 72 時間以内に治験調整委員会に薬剤名を連絡し、治験調整委員会は本情報

を治験責任医師に連絡する。また、割付責任者はデータセンターに緊急キーコードを開示したことを連絡する。

3) 被験者の組入れ又は割付に誤りがあった場合の手順

本治験の選択基準を満たさない被験者、又は除外基準に抵触する被験者の組入れ及び治験薬の投与はできない。

選択基準を満たさない又は除外基準に抵触する被験者が誤って、治験に組み入れられた場合は、被験者の安全性を確保することを常に最優先し、以下の手順を踏む。

① 治験責任医師等又はモニターは、治験調整委員会（治験調整事務局）に速やかに連絡する。

② 直接的に治験薬投与の継続が被験者に対する安全性のリスクになると考えられる場合は、必ず投与を中止する（治験調整委員会と治験責任医師等の協議後、被験者の治験薬投与中止を決定することもある）。治験責任医師は治験薬投与中止の理由を明確に記録する。被験者は、intention-to-treat の原則に則り、エンドポイントの追跡調査を含め規定の本治験に関わる手順に従って、追跡調査のため治験の終了まで治験の参加を継続する。

③ 治験薬投与を継続しても安全性及び疾患の管理に関わる懸念が生じないと判断された場合、治験責任医師は継続の理由を明確に記録する。被験者は、規定された本治験手順に従い、本治験を継続する。

8 治療計画

8.1 治療計画

本登録後、本登録日を含めて 28 日以内にプロトコル治療を開始する。

プロトコル治療は、下記の通りとする。

被験薬：シロスタゾール 50 mg 錠

対照薬：プラセボ錠

いずれかの薬剤である治験薬を 1 回 1 錠　1 日 2 回経口投与を 96 週継続する。治験薬投与開始日及び治験薬投与最終日についても 1 回 1 錠　1 日 2 回内服する。

なお、シロスタゾール錠（被験薬）とプラセボ錠（対照薬）は外観上区別されない錠剤である。

治験薬は本治験実施計画書に規定されている用途及び用法・用量以外で使用してはならない。

（シロスタゾール用量設定の根拠）

順天堂大学の新井らは、ドネペジル治療中のアルツハイマー型認知症患者10 例に対しシロスタゾールの追加投与を行い、10 例中 8 例においてベースラインから比べて 5－6 か月後のミニメンタルステート検査（MMSE）スコアの低下が抑制されたと報告しており（平均追跡期間 7.4 か月）、その際のシロスタゾール用量は 100 mg/日であった[10]。

また、田口らが行ったレトロスペクティブ解析においても、MMSE 22－26 点の患者群（ドネペジル塩酸塩非内服下）における、MMSE の年間低下率は、シロスタゾール 100 mg/日内服群（21 例）＋0.82/年、シロスタゾール 200 mg/日内服群（10 例）－0.42/年であった[11]。さらに、猪原らが行ったドネペジル治療中の患者を対象としたレトロスペクティブ解析においても、MMSE22－26 点の患者群（ドネペジル塩酸塩内服下）における、MMSE の年間低下率は、シロスタゾール 100 mg/日内服群（25 例）－0.41/年、200 mg/日内服群（10 例）－0.71/年であった[12]。これらの解析からシロスタゾールの用量について、100 mg/日と現在国内で脳梗塞の二次予防に用いられている 200 mg/日とでは、認知障害の進行抑制効果は遜色ないものと示唆される。

一方、間歇性跛行を呈する患者におけるシロスタゾール 100 mg/日とシロスタゾール 200 mg/日の比較を行ったメタ解析の報告がある[16]。頭痛の頻度が、プラセボ 13%、シロスタゾール 100 mg/日 26%、シロスタゾール 200 mg/日 33%と、有意差をもってシロスタゾール 100 mg/日の方が副作用としての頭痛が少ないことが報告されており、忍容性の観点からはシロスタゾール 100 mg/日の投与が妥当であると考えられる。

さらに、我々は脳梗塞モデルマウスにシロスタゾール 0.3%混餌飼料を用いて実験を行った。マウスの体重を 30 g、1 日飼料摂取量を 3 g と仮定するとシロスタゾール 300 mg/kg 経口投与に相当するが、その際の Cmax が約 0.5 μg/mL であった。一方、健康成人に本剤 100 mg 錠を単回経口投与した際の血中濃度が約 0.6 μg/mL でほぼ同じレベルだった。したがって、マウスで得られた効果をヒトで期待するためには、シロスタゾール 100 mg/日投与が必要であると思われる。シロスタゾール 50 mg/日のヒトへの投与では、上述の Cmax の観点からも、マウスと同様の効果を期待できないと考えられるため、本治験では、シロスタゾールの用量を 100 mg/日に設定した。

8.2 用量・スケジュール変更基準

用量の減量が必要となった場合、当該症例はプロトコル治療中止とする。

8.3 併用薬及び併用療法

8.3.1 併用禁止薬及び併用禁止療法

治験期間中の食事療法、リハビリテーション療法に制限は設けない。治験期間中に使用した薬剤については、併用禁止又は併用可能にかかわらず、薬剤名（商品名）、1 日量、投与開始日及び投与終了日をもれなく症例報告書に記載する。また、使用目的、投与経路を症例報告書に記載する。

プロトコル治療開始日からプロトコル治療中止・終了日まで、(1)の作用機序を有する薬剤を 2 剤以上併用することを禁止する（但し、シロスタゾールは 1 剤でも不可とする）。また、以下の(2)－(5)の作用機序を有する薬剤の併用を禁止する。すなわち、本治験薬（プラセボを含む）を含めずに血小板凝集抑制作用を有する薬剤を 2 剤以上併用することおよび抗凝固作用を有する薬剤、血栓溶解作用を有する薬剤、アルツハイマー病治療薬及び他の治験薬は併用を禁止する。なお、今後製造承認される以下の薬剤の併用についても同様に禁止する。

(1). 血小板凝集抑制作用を有する薬剤 アスピリン製剤（バファリン、バイアスピリンなど）、ジピリダモール（ペルサンチン、アンギナールなど）、硫酸クロピドグレル（プラビックス）、塩酸チクロピジン（パナルジンなど）、シロスタゾール（プレタールなど）、アルプロスタジル（プロスタンディン、リプル、パルクスなど、ただし局所性外用剤を除く）、リマプロストアルファデクス（プロレナール、オパルモンなど）、オザグレルナトリウム（キサンボン、カタクロットなど）、イコサペント酸エチル（エパデールなど）、ベラプロストナトリウム（ドルナー、プロサイリンなど）、塩酸サルポグレラート（アンプラーグ）、エポプロステノールナトリウム（フローランなど）、プラスグレル（エフィエント）

(2). 抗凝固作用を有する薬剤 ヘパリン製剤（ヘパリンナトリウムなど、ただし局所性外用剤を除く）、ダナパロイドナトリウム（オルガラン）、ワルファリンカリウム（ワーファリンなど）、アルガトロバン（ノバスタン、スロンノンなど）、ウリナスタチン（ミラクリッド、ウリナスタンなど）、メシル酸ガベキサート（エフオーワイなど）、メシル酸ナファモスタット（ストリームなど）、乾燥濃縮人アンチトロンビンⅢ（アンスロビンＰなど）、乾燥濃縮人活性化プロテイン C（アナクト C）、フォンダパリヌクスナトリウム（アリクストラ）、ダビガトラン（プラザキサ）、リバーロキサバン（イグザレルト）、アピキサバン（エリキュース）、エドキサバン（リクシアナ）

(3). 血栓溶解作用を有する薬剤 アルテプラーゼ（アクチバシン、グルトパなど）、ウロキナーゼ、チソキナーゼ（ハパーゼコーワ、プラスベータ）、モンテプラーゼ（クリアクター）、パミテプラーゼ（ソリナーゼ）、ナサルプラーゼ（トロンボリーゼ）、バトロキソビン（デフィブラーゼ）

(4). アルツハイマー病治療薬 ドネペジル（アリセプト、ドネペジル）、リバスチグミン （イクセロンパッチ、リバスタッチパッチ）、ガランタミン（レミニール）、メマンチン（メマリー）、抑肝散
(5). 他の治験薬

8.3.2 併用禁止薬の設定根拠

(1). 被験薬であるシロスタゾールと同様の血小板凝集抑制作用を有するため、併用した場合、過剰の薬理作用又は副作用が発現する可能性があり、被験者の安全性確保及び有効性の評価が困難となるため設定した。ただし、TOSS 試験の結果[17]および CATHARSIS 試験[18]の結果から、出血のリスクを増加させないと考えられるため、本治験薬を除く(1)の作用機序を有する薬剤 1 剤の併用は許容する。
(2). 血栓形成抑制作用を有し、併用した場合（1）と同様に被験者の安全性確保及び有効性の評価が困難となるため設定した。
(3). 血栓溶解作用を有するため、併用した場合、出血の副作用を助長する可能性が有り、被験者の安全性確保が困難となるため設定した。
(4). 一時的であるにせよ、アルツハイマー病治療薬の認知機能低下の抑制効果により、認知機能の評価に支障をきたす可能性があるため設定した。
(5). 安全性が確立されていないことより、被験者の安全性に配慮し設定した。

8.3.3 併用可能薬

併用禁止薬（前項）以外の薬剤は併用可能とする。ただし、途中併用薬の用量を変更した場合及び新たな薬剤を追加した場合は、薬剤名（商品名）、1日量、投与開始日及び投与終了日をもれなく症例報告書に記載する。また、使用目的、投与経路を症例報告書に記載する。（「11.2 観察・検査・調査項目 11.2.3 治療期 9）併用薬の服薬状況」参照）

8.3.4 被験者の安全性を確保するために行われる対応

(1). 緊急時の治療法

緊急の危険を回避するための治療及び発現した有害事象に対する治療はすべて許容する。また、「9.3 有害事象の報告と発現時の対応」の手順に従い報告及び追跡調査を行う。

(2). 妊娠が明らかになった場合の対応

治験責任医師又は治験分担医師は、女性被験者の妊娠または男性被験者のパートナーの妊娠が明らかになった場合、当該被験者のプロトコル治療を中止する。また、結果として有害事象が発現していない場

合も含めて、「10.2.1 妊娠の緊急報告」「10.2.2 妊娠の追跡調査」の手順に従い治験調整委員会に文書で報告するとともに、当該女性被験者または妊娠パートナーの分娩又は妊娠終了まで追跡調査を行う。

投与を中止するときは、治験実施計画書に規定された中止時検査及び経過観察を、妊娠に影響がない範囲で行うこと。

(3). 過量服薬、乱用、誤用または投与過誤の場合の処置

シロスタゾールを過量服薬、乱用、誤用または投与過誤した場合のデータはないが、治験責任医師又は治験分担医師は一時的な休薬などを行う。また、結果として有害事象が発現していない場合も含めて、「10.2.3 過量投与、乱用、誤用、投与過誤、本治験薬が原因の感染症の緊急報告」「10.2.4 過量投与、乱用、誤用、投与過誤、本治験薬が原因の感染症の追跡調査報告」の手順に従い報告及び追跡調査を行う。

(4). 本治験薬が原因の感染症が生じた場合の処置

ただちに、当該感染症治療などの処置を行う。また、結果として有害事象が発現していない場合も含めて、「10.2.3 過量投与、乱用、誤用、投与過誤、本治験薬が原因の感染症の緊急報告」「10.2.4 過量投与、乱用、誤用、投与過誤、本治験薬が原因の感染症の追跡調査報告」の手順に従い報告及び追跡調査を行う。

(5). シロスタゾール投与時に予想される副作用に対する処置

調査症例 4890 例中 436 例（8.92%）に臨床検査値の異常を含む副作用が認められている。

1） 市販後の頻度不明の副作用を含めて下記の重大な副作用があらわれることがあるので、観察を十分に行い、症状や異常が認められた場合には適切な処置を行う。

・うっ血性心不全、心筋梗塞、狭心症、心室頻拍
・出血（脳出血等の頭蓋内出血、肺出血、消化管出血、鼻出血、眼底出血等）
・胃・十二指腸潰瘍
・汎血球減少、無顆粒球症、血小板減少
・間質性肺炎（発熱、咳嗽、呼吸困難、胸部 X 線異常、好酸球増多を伴う）
・肝機能障害（AST（GOT）、ALT（GPT）、Al-P、LDH 等の上昇）、黄疸
・急性腎不全

2） 重大な副作用以外に下記の副作用（5%以上、5%未満又は頻度不明）があらわれることがあるので、同様な症状の発現がみられた場合は、安静にして回復をはかるなど適切な処置を行う。

（5%以上）
・頭痛・頭重感

（0.1～5%未満の頻度）
・発疹、皮疹、そう痒感
・動悸、頻脈、ほてり、血圧上昇、血圧低下、心房細動・上室性頻拍・上室性期外収縮・心室性期外収縮等の不整脈等
・めまい、不眠、しびれ感
・腹痛、悪心・嘔吐、食欲不振、下痢、胸やけ、腹部膨満感、味覚異常
・貧血、白血球減少等
・皮下出血、血尿等
・AST（GOT）、ALT（GPT）、Al-P、LDH の上昇等
・BUN 上昇、クレアチニン上昇、尿酸値上昇、頻尿
・発汗、浮腫、胸痛、血糖上昇、耳鳴、倦怠感、発熱、脱毛

（0.1%未満の頻度）
・蕁麻疹等
・眠気、振戦等、肩こり、失神・一過性の意識消失等
・口渇等
・好酸球増多等
・排尿障害等
・疼痛、筋痛、脱力感

（頻度不明）
・光線過敏症、紅斑

8.4 服薬、その他の取り決め事項の遵守状況の確認

治験責任医師、治験分担医師、治験協力者又は治験薬管理者は、「治験薬の管理に関する手順書」に従い、被験者の服薬の遵守状況の確認等の管理を行う。

治験薬の服薬の遵守状況の確認及び服薬指導

治験責任医師又は指名された者は各来院時に被験者の服薬等の遵守状況を確認する。服薬が遵守されていない被験者には指示されたとおりに治験薬を服用することの重要性を各来院時に伝える。

治験薬の服薬状況の確認

治験責任医師、治験分担医師又は指名された者は治療開始後第 24 週・第 48 週・第 72 週・第 96 週の各期間の治験薬の服薬状況を、被験者から回収した残薬数及び被験者への問診に基づき確認し、以下の 3 段階の基準にて評価を行い症例報告書に記載する。

(1). 80%以上治験薬を服薬
(2). 80%未満 50%以上治験薬を服薬
(3). 50%未満治験薬を服薬

各期間の服薬状況が 80%未満と評価された被験者には注意喚起・服薬再指導を実施する。

8.5 中止基準（プロトコル治療を中止する基準）

8.5.1 プロトコル治療の中止基準

治験責任医師は、以下の事例が発現した場合、速やかに当該被験者のプロトコル治療を中止として記録する。

1） 被験者がプロトコル治療中止を申し出た場合
2） 当該被験者が同意を撤回した場合
3） 有害事象により治験責任医師又は治験分担医師がプロトコル治療継続不能と判断した場合
4） 脳梗塞及び脳出血により入院加療が必要となった場合
5） 新たに 3 剤目の抗血小板薬（アスピリン、クロピドグレル等）を服用しなければならなくなった場合（本治験薬も含む）
6） 新たに抗凝固薬（ワルファリンカリウム、ダビガトラン等）を服用しなければならなくなった場合
7） 被験者又は被験者のパートナーが妊娠した場合
8） 認知症の確定診断がなされた場合
9） プロトコル治療中の死亡
10） 本登録後、適格基準を満たしていないことが判明した場合（治験薬投与を継続しても被験者の安全性及び疾患の管理に関わる懸念が生じないと判断された場合は除く）
11） 転院等の理由により当該被験者の追跡が継続できない場合
12） その他、治験責任医師又は治験分担医師がプロトコル治療継続不能と判断した場合

8.5.2 プロトコル治療中止の取り扱い

プロトコル治療を中止した被験者について、治験責任医師又は治験分担医師は被験者がプロトコル治療中止時の来院及び治験のための来院の継続を了承するかどうかを必ず確認する。被験者の回答を踏まえ、下記の①～③の手順で該当する内容を実施する。転院等で治験のための来院ができない場合でも可能な限り転院先からの転帰情報を入手し、入手方法と入手年月日をカルテに記載する。

① 被験者がプロトコル治療中止時の来院及び治験のための来院の継続を了承する場合
治験責任医師又は治験分担医師は被験者及びスタディパートナーにプロトコル治療中止時の来院を要請する。治験責任医師又は治験分担医師は治験スケジュール（11.3.観察・検査・調査スケジュール）に従い、治療のための来院を被験者及びスタディパートナーに要請する。データの収集は治験実施計画書に従って継続する。

② 被験者がプロトコル治療中止時の来院は了承するが、治験のための来院の継続は了承しない場合
治験責任医師又は治験分担医師は被験者及びスタディパートナーにプロトコル治療中止時の来院を要請する。

③ 被験者がプロトコル治療中止時の来院及び治験のための来院の継続を了承しない場合
被験者の同意撤回とする。上述の決定を治験責任医師又は治験分担医師はカルテに記録する。

なお、下記の 1)～2)に該当する被験者は中止時検査及び治験のための来院を行わない。

1） 当該被験者が死亡した場合
2） その他の理由により追跡が困難な場合

8.6 後治療

プロトコル治療中止後、後治療は制限しない。

9 有害事象の評価・報告

9.1 重篤及び重篤でない有害事象の定義

有害事象（AE: Adverse Event）とは、治験薬を投与された被験者に生じたあらゆる好ましくない医療上のできごと、すなわち、治験薬を投与された被験者に生じたすべての好ましくないあるいは意図しない疾病又はその徴候（臨床検査値の異常を

含む）のことで、治験薬との因果関係の有無は問わない。

重篤な有害事象（SAE: Serious Adverse Event）とは、有害事象のうち、以下のものをいう。

1. 死亡
2. 死亡につながるおそれのあるもの
3. 治療のために病院又は診療所への入院又は入院期間の延長が必要となるもの
4. 障害
5. 後世代における先天性の疾病又は異常
6. その他、1～5 に準じて重篤であるもの

重篤でない有害事象は、「重篤な」有害事象の基準を満たさないすべての有害事象のことである。（プロトコル治療開始以前からの予定入院及び治験実施計画書に規定されている検査のための入院は除く。）

9.2 有害事象の評価及び判定基準

治験薬投与開始日から治験薬投与終了日の翌日までに発現した有害事象は、因果関係の有無に関わりなくすべて観察対象とし、「11.3.　観察・検査・調査スケジュール」で定めたスケジュールに基づき評価し、事象名、発現日、重症度、重篤度、因果関係、転帰、転帰日を症例報告書によって報告する。

なお、治験薬投与終了後、有害事象の追跡調査期間中に新たに重篤な有害事象が発現した場合は、「9.3.2 有害事象の追跡調査」を参照する。

9.2.1 有害事象の重症度の評価

有害事象の重症度は、以下のいずれかの区分とする。

1. 軽度：無症状又は症状が軽く、日常生活に支障を来さない程度
2. 中等度：高度な障害でないが、日常生活に支障を来す程度
3. 高度：障害が高度で、日常生活を送ることが不可能

9.2.2 有害事象の重篤度

有害事象の重篤度は、以下のいずれかとする。

1. 非重篤
2. 重篤

9.2.3 因果関係の区分

治験薬との因果関係は、以下のいずれかの区分で判定する。

1. 関連あり

2. 関連あるかもしれない
3. 関連なし

9.2.4 有害事象の転帰

有害事象の転帰は、以下のいずれかの区分とする。

1. 回復：有害事象が消失し、発現前の状態に回復した
2. 軽快：有害事象がほぼ消失し、有害事象発現前に近い状態に戻った
3. 未回復：有害事象が消失せず、有害事象発現時と同様の状態にある（不変）
4. 回復したが後遺症あり：有害事象は消失したが、後遺症がある
5. 死亡：被験者が死亡した（因果関係は問わない）
6. 不明：情報がなく、転帰が不明

9.3 有害事象の報告と発現時の対応

9.3.1 有害事象発現時の対応

治験責任医師及び治験分担医師は、被験者に有害事象が発現した場合、まず被験者の治療・安全を確保するとともに、適切な処置を行う。治験分担医師は、治験薬との因果関係を問わず、速やかに治験責任医師に報告する。

治験責任医師及び治験分担医師は、発現した有害事象について以下の評価を行う。有害事象が複数発現した場合には事象別に判断する。

1. 被疑薬／被疑機器の特定
2. 発現した有害事象の重篤性及び重症度
3. 治験薬との因果関係の有無
4. 予測可能性

9.3.2 有害事象の追跡調査

(1). プロトコル治療中止・終了後 10 日において回復している有害事象の追跡調査は行わない。

(2). プロトコル治療中止・終了後 10 日において回復していない有害事象は、プロトコル治療中止・終了後 38 日以内に追跡調査を行う。治験責任医師又は治験分担医師は、該当する被験者に追跡調査の主旨を説明し、協力を依頼する。

(3). 追跡調査で回復していない有害事象について追跡調査を継続するか、打ち切るかについては治験薬との因果関係により判断する。

1） 治験薬との因果関係が「関連なし」の場合は、その後の追跡調査を打ち切る。

2） 治験薬との因果関係が「関連あるかもしれない」又は「関連あり」の

場合は、事象が回復又は安定化するまで追跡調査を継続する。

3) 追跡調査期間中に「重篤」に該当する有害事象になった場合、又は新たに重篤な有害事象が発現した場合は、重篤な有害事象として、「9.3.4 報告手順」に従う。治験薬との因果関係が「関連なし」の場合は、その後の追跡調査を打ち切る。治験薬との因果関係が「関連あるかもしれない」又は「関連あり」の場合は、事象が回復又は安定化するまで追跡調査を継続する。

9.3.3 緊急報告対象事象

緊急報告義務のある有害事象

・「死亡または生命を脅かす予測できない有害事象」

・「その他の重篤で予測できない有害事象」

9.3.4 報告手順

- 重篤な有害事象が発生したとき、治験責任医師等は、「安全性情報の取扱に関する手順書」に従う。治験責任医師又は治験分担医師が重篤な有害事象を認知した時点から、24 時間以内に治験調整委員会が定める様式を用いて速報にて報告する。また、治験責任医師は各実施医療機関が別途定める様式を用いて、実施医療機関の長に報告する。
- 治験調整委員会は、当該有害事象が 7 日報告又は 15 日報告（医薬品、医療機器等の品質、有効性及び安全性の確保等に関する法律施行規則第 273 条第 1 項第 1 号及び 2 号）に該当するか判断し、厚生労働大臣への報告が必要と判断した場合、治験責任医師からの委嘱に基づき、「安全性情報の取扱に関する手順」に従い判断した結果及び有害事象等の内容を別途定める様式を用いて独立行政法人医薬品医療機器総合機構に報告する。
- 治験調整委員会は、他の実施医療機関の治験責任医師及び治験薬提供者にも本情報を通知する。
- 他の実施医療機関の治験責任医師は、医薬品医療機器総合機構に報告した重篤な有害事象について、可能な限り速やかにその内容を文書で実施医療機関の長に報告する。
- 治験調整委員会及び治験責任医師等は、詳細情報及び追加情報についても同様に可能な限り速やかに「安全性情報の取扱に関する手順」に従い実施医療機関の長及び治験調整委員会に報告する。当該追加情報の取扱いは本項「9.3.4 報告手順」の手順に準ずることとし、必要に応じ医薬品医療機器総合機構等への報告等を行う。

9.4 予測される有害事象等

本治験において予測される有害事象については治験薬概要書を参照のこと。

10 その他の安全性情報の報告

10.1 その他の安全性情報の定義

その他の安全性情報とは、以下の事象に関する情報のことである。これらを認めた場合、情報を収集し，所定の手順に従い報告を行う。なお、同時に有害事象が発現している場合は、当該事象を「9 有害事象の評価・報告」の手順に従い有害事象としても報告すること。

・妊娠中または授乳中の投与（有害事象が発現していない場合も含む)、
　または胎芽か胎児が本治験薬に曝露されていた可能性があったことの報告
　（母体内曝露あるいは本治験薬投与後の父親の精液を介しての遺伝)
・過量投与、乱用、誤用、投与過誤（結果として有害事象が発現していない場合も含む)
・本治験薬が原因の感染症

10.2 その他の安全性情報の報告手順

10.2.1 妊娠の緊急報告

治験責任医師又は治験分担医師は、治験期間中に女性被験者の妊娠または男性被験者のパートナーの妊娠が明らかになった場合は、治験調整委員会に可能な限り速やかに口頭、電話又は電子メールにて報告する（緊急連絡先：別紙 1「治験責任医師、各種委員会及び治験実施体制」を参照)。その後、治験調整委員会から更に追加の情報の提供を求められた場合には、別途提示される様式を用いて治験調整委員会に文書で報告する。

治験調整委員会は、治験薬提供者に本情報を通知するとともに、必要に応じ他の実施医療機関の治験責任医師にも本情報を通知する。

10.2.2 妊娠の追跡調査

治験責任医師又は治験分担医師は、妊娠した女性被験者又は妊娠パートナーの分娩又は妊娠終了まで追跡調査し、詳細情報及び追加情報についても同様に治験調整委員会に文書で報告する。なお、女性被験者又は妊娠パートナーが分娩した場合、分娩後少なくとも 6 か月間は新生児を観察することが望ましい。

当該追加情報の取扱いは「10.2.1 妊娠の緊急報告」の手順に準ずることとし、必要に応じ治験調整委員会より医薬品医療機器総合機構等への報告等を行う。

10.2.3 過量投与、乱用、誤用、投与過誤、本治験薬が原因の感染症の緊急報告

治験責任医師又は治験分担医師は、過量投与、乱用、誤用、投与過誤、本治験薬が原因の感染症に関する安全性情報が発生した場合は、治験調整委員会に可能な限り速やかに口頭、電話又は電子メールにて報告する（緊急連絡先：別紙 1 を 参照)。その後、治験調整委員会から更に追加の情報の提供を求められ

た場合には、別途提示される様式を用いて治験調整委員会に文書で報告する。

治験調整委員会は、治験薬提供者に本情報を通知するとともに、必要に応じ他の実施医療機関の治験責任医師にも本情報を通知する。

10.2.4　過量投与、乱用、誤用、投与過誤、本治験薬が原因の感染症の追跡調査報告

治験責任医師又は治験分担医師は、詳細情報及び追加情報についても同様に治験調整委員会に報告する。

当該追加情報の取扱いは「10.2.3 過量投与、乱用、誤用、投与過誤、本治験薬が原因の感染症の緊急報告」の手順に準ずることとし、必要に応じ治験調整委員会より医薬品医療機器総合機構等への報告等を行う。

11 観察・検査・調査項目とスケジュール

11.1 治験期間

被験者ごとのスケジュールの各期間を以下の通り定義する。

治験期間：　　　　　　同意取得日から最終評価日まで

スクリーニング期間：同意取得日から本登録前まで

導入期間：　　　　　　本登録日からプロトコル治療開始前まで

プロトコル治療期間：治験薬投与開始日から治験薬投与最終日まで

（プロトコル治療開始日は治験薬の投薬開始日である。）

11.2 観察・検査・調査項目

11.2.1 スクリーニング期

スクリーニング期間：同意取得日から本登録前まで

治験責任医師又は治験分担医師は、同意取得後から仮登録までの間に、下記の項目について調査を行う。神経心理検査の結果については心理検査中央判定委員会が判定する。心理検査中央判定委員会の本登録を以てスクリーニング期間は終了となる。

1）　基本情報

被験者識別コード、仮登録日、性別、生年月日、同意取得日

身長、体重、BMI（自動計算）、

年齢（同意取得時）（自動計算）

2）　病歴聴取

アルツハイマー型認知症の家族歴（両親・兄弟・子供・孫までを範囲とし、近親婚の有無も確認する）

教育程度（0－7 年，8－12 年，13－15 年，16 年以上）

既往歴（脳卒中、心筋梗塞、頭部外傷、全身麻酔、その他）

合併症（高血圧、糖尿病、脂質異常症、その他）

服薬歴（同意取得日前 28 日以降の、薬剤名（商品名）・１日量・使用開始日・使用終了日・投与経路・使用目的を確認する）

アルコール摂取の有無及び頻度

喫煙の有無、量及び期間

3）　身体所見

血圧、脈拍

4）　神経所見

意識障害の有無

5）　神経心理検査

MMSE（邦訳として MMSE-J を使用）
CDR（邦訳として CDR-J を使用）

本治験では治験調整委員会（または認知神経科学会）が企画する神経心理検査講習会及び認定試験を受講した者のみが、神経心理検査を行うこととする。また、学習効果のため頻回の神経心理検査は実施困難であるため、講習会を受講した検者がこれらの検査を同意取得前に行った場合は、仮登録日より前の 8 週以内の検査であれば、スクリーニング時の検査として許容する。

また、MMSE および CDR の評価判定は心理検査中央判定委員会で行う。

11.2.2 導入期 （ベースライン値判定期間）

導入期間：本登録日からプロトコル治療開始前まで

治験責任医師又は治験分担医師は、心理検査中央判定委員会による本登録が実施されてからプロトコル治療開始するまでの間に、下記の項目について調査を行い報告する。なお、MMSE および CDR のベースライン値はスクリーニング時の結果を用いる。

他の神経心理検査に関しては、短期間で反復して実施することによる被検査者の学習効果を考慮し、本登録日より前の 8 週以内に実施した検査の結果をベースライン値のデータとして採用することができる。

1） 病歴聴取
既往歴（脳卒中、心筋梗塞、頭部外傷、全身麻酔、その他）
合併症（高血圧、糖尿病、脂質異常症、その他）

2） 身体所見 （スクリーニング時の検査をベースライン値とする）
血圧、脈拍

3） 神経所見
意識障害の有無
失語、失行、失認の有無

4） 臨床検査 A
赤血球数、血色素量、ヘマトクリット、白血球数、血小板数、 総タンパク、総ビリルビン、AST、ALT、ALP、LDH、γ-GTP、BUN、クレアチニン、尿酸、CK、総コレステロール、トリグリセリド、血糖、HbA1c、Na、K、Cl、アルブミン

5） 血清アルブミン-Aβ複合体濃度

6） 神経心理検査

MMSE（スクリーニング時の検査をベースライン値とする）

CDR（スクリーニング時の検査をベースライン値とする）

ADAS-Cog 14（邦訳として ADAS-Cog-J 14 を使用）

WMS-R Logical Memory I & II（日本版ウエクスラー記憶検査法　論理的記憶 I と論理的記憶 II を使用）

TMT Part A および B

FCSRT（自由 - 手掛かり想起検査）

FCSRT は一部の治験参加施設の被験者においてオプショナルに実施する。

本治験では治験調整委員会（または認知神経科学会）が企画する神経心理検査講習会及び認定試験を受講した者のみが、神経心理検査を行うこととする。評価判定は心理検査中央判定委員会が行う。

7） ADCS-MCI-ADL

ADL　18 項目（課題 1－18；0－53 点）

手段的日常生活動作（IADL）6 項目（課題 19－24；0－16 点）

本治験では治験調整委員会（または認知神経科学会）が企画する神経心理検査講習会及び認定試験を受講した者のみが、ADCS-MCI-ADL を行うこととする。評価判定は心理検査中央判定委員会が行う。

8） 頭部 MRI

総海馬容積

MRI は一部の治験参加施設の被験者においてオプショナルに実施する。3D-T1 強調像、FLAIR 画像、T2*強調画像を撮影する。MRI の所見はプロトコル治療開始後、ただちに変化することはないため、プロトコル治療開始前後 8 週以内に撮影した画像であれば、ベースラインの画像として許容する。また、総海馬容積の計測は画像診断中央判定委員会にて実施する。

9） 併用薬の服薬状況

薬剤名（商品名）、1 日量、投与開始日、投与終了日、投与経路、使用目的

11.2.3　治療期

A). プロトコル治療期間：治験薬投与開始日～治験薬投与最終日

B). 治療期の調査時期：治療開始後第 4 週以内・第 24 週・第 48 週・第 72 週・第 96 週

（許容範囲：治療開始後第 4 週以内を除き、予定日の前後 14 日以内）

C). 治療期の調査項目

1） プロトコル治療期間

プロトコル治療開始日、プロトコル治療終了日

2） 身体所見

血圧、脈拍

3） 神経所見

認知症の有無、意識障害の有無

4） 臨床検査 B（治療開始後第 4 週以内）

赤血球数、血色素量、ヘマトクリット、白血球数、血小板数、 総タンパク、総ビリルビン、AST、ALT、ALP、LDH、γ-GTP、BUN、クレアチニン、尿酸、CK、総コレステロール、トリグリセリド、血糖、Na、K、Cl

5） 臨床検査 C（治療開始第 24 週）

アルブミン

6） 血清アルブミン-Aβ複合体濃度 （治療開始後第 24 週）

7） 神経心理検査

MMSE （治療開始後第 24 週・第 48 週・第 72 週・第 96 週）

CDR、ADAS-Cog 14、WMS-R Logical Memory I & II、TMT Part A および B、FCSRT （治療開始後第 48 週第 96 週）

FCSRT は一部の治験参加施設の被験者においてオプショナルに実施する。

本治験では治験調整委員会（または認知神経科学会）が企画する神経心理検査講習会及び認定試験を受講した者のみが、神経心理検査を行うこととする。評価判定は心理検査中央判定委員会が行う。

8） ADCS-MCI-ADL（治療開始後第 48 週・第 96 週）

ADL 18 項目（課題 1－18；0－53 点）

手段的日常生活動作（IADL）6 項目（課題 19－24；0－16 点）

本治験では治験調整委員会（または認知神経科学会）が企画する神経心理検査講習会及び認定試験を受講した者のみが、ADCS-MCI-ADL を行うこととする。評価判定は心理検査中央判定委員会が行う。

9）頭部 MRI（一部の治験参加施設においてオプショナルに実施、治療開始後第 96 週）
総海馬容積

総海馬容積の計測は画像診断中央判定委員会にて実施する。

10）治験薬の服薬状況
（ただし、プロトコル治療中止時より後の調査時期は除く）

11）併用薬の服薬状況
薬剤名（商品名）、1 日量、投与開始日、投与終了日、投与経路、使用目的

12）有害事象
事象名、発現日、重篤度、因果関係、転帰、転帰日
（治験薬投与終了後は除く。ただし、有害事象の追跡調査期間中に新たに重篤な有害事象が発現した場合は、「9.3.2 有害事象の追跡調査」を参照すること。）

13）転帰調査（MCI、「アルツハイマー型認知症の確診」、「アルツハイマー型認知症の疑診」、他の原因による認知症*、正常、死亡、判定不能）
評価日（被験者が死亡した場合は、死亡日）
死亡原因　（被験者が死亡した場合）

*他の原因による認知症とは、「アルツハイマー型認知症の確診」、「アルツハイマー型認知症の疑診」以外のすべての認知症を指す。

11.2.4　中止時

中止時検査に関しては、中止判断日後 28 日以内に実施する。また、被験者の負担を考慮し、中止時の神経心理検査及び ADCS-MCI-ADL に関しては、中止判断日の 8 週前までに実施した検査があった場合、その検査結果

を中止時のデータとして採用することができる。

A). 中止時の調査項目

1） 中止判断日、中止時検査実施日

2） 身体所見
血圧、脈拍

3） 神経所見
認知症の有無、意識障害の有無

4） 臨床検査 B
赤血球数、血色素量、ヘマトクリット、白血球数、血小板数、 総タンパク、総ビリルビン、AST、ALT、ALP、LDH、γ-GTP、BUN、クレアチニン、尿酸、CK、総コレステロール、トリグリセリド、血糖、Na、K、Cl

5） 神経心理検査
MMSE、CDR、ADAS-Cog 14、WMS-R Logical Memory I & II、TMT Part A および B、FCSRT

FCSRT は一部の治験参加施設の被験者においてオプショナルに実施する。

本治験では治験調整委員会（または認知神経科学会）が企画する神経心理検査講習会及び認定試験を受講した者のみが、神経心理検査を行うこととする。評価判定は心理検査中央判定委員会が行う。

6） ADCS-MCI-ADL
ADL 18 項目（課題 1－18；0－53 点）
手段的日常生活動作（IADL）6 項目（課題 19－24；0－16 点）

本治験では治験調整委員会（または認知神経科学会）が企画する神経心理検査講習会及び認定試験を受講した者のみが、ADCS-MCI-ADL を行うこととする。評価判定は心理検査中央判定委員会が行う。

7） 頭部 MRI (一部の治験参加施設においてオプショナルに実施)
総海馬容積

総海馬容積の計測は画像診断中央判定委員会にて実施する。

8） 治験薬の服薬状況

9） 併用薬の服薬状況
薬剤名（商品名）、1 日量、投与開始日、投与終了日、投与経路、使用目的

10） 有害事象
事象名、発現日、重篤度、因果関係、転帰、転帰日

11） 転帰調査（MCI、「アルツハイマー型認知症の確診」、「アルツハイマー型認知症の疑診」、他の原因による認知症*、正常、死亡、判定不能）
評価日（被験者が死亡した際は、死亡日）
死亡原因 （被験者が死亡した場合）

*他の原因による認知症とは、「アルツハイマー型認知症の確診」、「アルツハイマー型認知症の疑診」以外のすべての認知症を指す。

11.3　観察・検査・調査スケジュール

検査・観察・評価項目 ＼ 時期	同意取得	スクリーニング		導入期(ベースライン)		治療期					中止時
						治療開始後第 4 週以内	治療開始後第 24 週	治療開始後第 48 週	治療開始後第 72 週	治療開始後第 96 週	
同意取得	●		仮登録<br>心理検査中央評価<br>本登録		治療開始						
無作為化				●							
基本情報<br>病歴聴取		●		●							
神経所見		●		●		●	●	●	●	●	●
臨床検査 A				●							
臨床検査 B						●					●
臨床検査 C							●				
血清アルブミン-Aβ複合体濃度測定				●			●				
画像検査（脳 MRI）注 1				●						●	●
神経心理検査 MMSE		●					●	●	●	●	●
神経心理検査 CDR		●						●		●	●
神経心理検査 ADAS, WMS-R, TMT, FCSRT 注 1				●				●		●	●
ADCS-MCI-ADL				●				●		●	●
併用薬の服薬状況の確認				←	―	―	―	―	―	―	→
治験薬の服薬状況の確認						←	―	―	―	―	→
有害事象					←	―	―	―	―	―	→
転帰調査						←	―	―	―	―	→
許容範囲			仮登録後 4 週以内に本登録<br>本登録後 4 週以内に治療開始				前後<br>14 日以内	前後<br>14 日以内	前後<br>14 日以内	前後<br>14 日以内	中止後<br>28 日以内

注 1：一部の症例において実施.

12 目標登録症例数と治験実施予定期間

12.1 目標登録症例数

200 例（各群合わせて）

被験薬群、対照薬群ごとに 100 例

12.2 治験実施予定期間

症例登録期間　：2015 年 5 月から 2018 年 3 月（終了予定）（35 か月間）

（ただし、登録期限前に目標症例数の 200 例に達した場合等は、その時点で終了とする）

治験期間　：2015 年 5 月から 2020 年 3 月（終了予定）

（最終症例登録日から 2 年後）

13 評価項目の定義

13.1 主要評価項目

MMSE（邦訳として MMSE-J を使用）スコアの変化量（シリアル 7 課題のスコアを用いる）

（スクリーニング時と治療開始後第 24、48、72、96 週の差分）

13.2 副次評価項目

臨床効果の評価指標：

1） MCI から「あらゆる原因による認知症」への移行までの期間
2） CDR-SB（邦訳として CDR-J を使用）の変化量
3） ADAS-Cog 14（邦訳として ADAS-Cog-J 14 を使用）の変化量
4） WMS-R Logical Memory（日本版ウエクスラー記憶検査法　論理的記憶 I と論理的記憶 II を使用）の変化量
5） ADCS-MCI-ADL を用いた ADL（18 項目：0－53 点）の変化量
6） MRI で測定される海馬容積の変化量　(オプショナル)

13.3 探索的評価項目

1） IADL：ADCS-MCI-ADL（課題 19－24）の変化量
2） 神経心理検査：TMT Part A および B スコアの変化量
3） 神経心理検査：FCSRT スコアの変化量(オプショナル)
4） 血清アルブミン-Aβ複合体濃度の変化量

13.4 エンドポイントの定義

13.4.1 あらゆる原因による認知症（All-cause Dementia）

以下に該当する認知または行動（精神神経）症状が認められる場合は、「あらゆる原因による認知症」と診断する[17]。なお、DSM-5 基準では Major Neurocognitive Disorder に該当する。

1. 仕事中または日常活動時の遂行能力を低下させている。
2. 機能および遂行の水準が以前から低下したことを反映している。
3. せん妄または主要な精神疾患では説明できない。
4. (1)被験者本人および被験者をよく知る情報提供者からの病歴聴取と（2）客観的認知機能評価（「ベッドサイド用」の精神状態検査か神経心理検査のいずれか）の組み合わせにより、認知障害が検出および診断される。神経心理検査は、ルーチンの病歴聴取とベッドサイド用の精神状態検査では確定的な診断が得られない場合に施行すること。
5. 認知面または行動面の障害が以下の領域の 2 つ以上に関係している

a 新しい情報を受け取って記憶する能力の低下：具体的な症状としては、同じ質問や会話を繰り返す、自分の物を置き忘れる、行事や約束事を忘れる、よく慣れた道で迷う、などが挙げられる。

b 複雑な課題を推論・処理する能力の低下、判断力の低下：具体的な症状としては、安全を脅かすリスクを適切に理解できない、財務管理ができない、意思決定能力の低下、複雑な活動や一連の活動の計画を立てられない、などが挙げられる。

c 視空間能力の低下：具体的な症状としては、視力が良好であるにもかかわらず、人の顔や一般的な物品を認識できない、直視しても物体を見つけ出すことができない、単純な用具を操作できない、身体の方向に合わせて衣服を着用できない、などが挙げられる。

d 言語機能（話す、読む、書く）の障害：具体的な症状としては、会話中に一般的な単語を思いつかない、口ごもる、会話・綴り・書字を誤る、などが挙げられる。

e 人格、行動または態度の変化：具体的な症状としては、激越などの普段みられない気分変動、意欲や自発性の低下、無感情、

活力の喪失、引きこもり、かつて行っていた活動への関心の低下、共感の喪失、強迫観念または強迫行動、社会的に容認されない行動などが挙げられる。

認知症と MCI との鑑別は CDR の値を参考に、仕事中または日常活動中に顕著な遂行能力の低下がみられるか否かによって判定する。この点については、個々の被験者の状況と、被験者本人および被験者をよく知る情報提供者による被験者の日常生活に関する説明に基づいて、経験豊富な臨床医が臨床的に判断する。

13.4.2　アルツハイマー型認知症の確診（Probable AD Dementia）

認知症の基準に適合し、さらに以下の特徴が認められる患者は、「アルツハイマー型認知症の確診」と診断する[17]。

A.　潜行性の発症 : 症状が数か月から数年をかけて徐々に発現した（数時間から数日の急激な発現ではない）。

B.　状態悪化の経緯が明確に報告または観察されている。

C.　最初に認められた最も顕著な認知機能の低下が、病歴と診察から明白であり、かつ以下のカテゴリーのどちらかに該当する。

a　健忘型の場合（Amnestic presentation）

アルツハイマー型認知症の最も一般的な臨床像である。学習の障害と最近学習した情報の再生の障害が認められなければならず、また上記で定義した他の認知機能領域についても、認知障害を示す所見が認められていなければならない。

b　非健忘型の場合（Nonamnestic presentations）

- 言語機能の障害：語想起に最も顕著な障害が認められる。ただし、他の認知機能領域にも障害がなければならない。
- 視空間機能の障害 : 空間認知に最も顕著な障害が認められる（物体失認、顔認識障害、同時失認、失読など）。ただし、他の認知機能領域にも障害がなければならない。
- 遂行機能の障害：推論、判断および問題解決に最も顕著な障害が認められる。ただし、他の認知機能領域にも障害がなければならない。

D. 以下の条件に該当する患者については、「アルツハイマー型認知症の確診」と診断してはならない。

(a) 有意な脳血管疾患の併発（認知障害の発症または増悪と時間的関連性が認められる脳卒中の既往歴がある場合と定義)、もしくは複数または広範な梗塞あるいは重度の白質高信号病変の存在

(b) レビー小体型認知症のコア特徴（認知症自体は除く）

(c) 行動型前頭側頭型認知症の顕著な特徴

(d) semantic variant 原発性進行性失語症または nonfluent/agrammatic variant 原発性進行性失語症の顕著な特徴

(e) 以上に該当しない活動性の神経疾患の併発、神経疾患以外の併存症、または認知機能に大きな影響を及ぼしうる薬剤の使用

注意：1984 年の NINCDS-ADRDA 基準[20]で「probable AD」と診断される患者は、すべて、本文書で示した「アルツハイマー型認知症の確診」の基準に適合すると考えられる。

13.4.3 アルツハイマー型認知症の疑診（Possible AD Dementia）

以下に示す状況のいずれかに該当する場合は、「アルツハイマー型認知症の疑診」と診断すること[19]。

A. 非定型的な経過をたどる場合

これは、認知機能低下の性質という点ではアルツハイマー型認知症の臨床診断基準のコア項目に適合するが、認知障害の発症が突然であるか、進行性の認知機能低下について詳細な病歴データや客観的な証拠が不十分な場合である。

B. 複数の病因が混在していると考えられる場合

これは、アルツハイマー型認知症の臨床診断基準のコア項目に適合するが、

(a) 脳血管疾患の併発（認知障害の発症または増悪と時間的関連性が認められる脳卒中の既往歴がある場合と定義）、もしくは複数または広範な梗塞あるいは重度の白質高信号病変の存在

(b) 認知症自体を除いたレビー小体型認知症のコア特徴

(c) 以上に該当しない神経疾患の併発、神経疾患以外の併存症、

または認知機能に大きな影響を及ぼしうる薬剤の使用
以上のいずれかが認められる場合である。

14 統計学的考察

14.1 目標登録症例数及びその設定の根拠

目標症例数：200 例（被験薬群 100 例、対照薬群 100 例）

【設定の根拠】

標本数は各群 100 例とする。この症例数は目標達成の可能性から定めた。しかしながら、この標本数は Type I エラー5%、検出力 90%及び標準偏差 3.7 の条件下で、本登録時から治療開始後第 96 週の MMSE スコア変化量の差分について両群間でその平均値を検定した場合、1.72 の群間差を検出できるものである。この標準偏差は米国 ADNI 研究グループが公開しているデータ [21] 及び田口らの研究 [11] を参照した。また、この標本数設定の方法は Julious[22] に従った。

14.2 解析対象集団

14.2.1 解析対象集団

本治験の主要評価項目及び副次評価項目について、臨床効果に関しては、最大の解析対象集団（FAS）を解析対象集団とする。ただし、主要評価項目に対しては、治験実施計画書に適合した対象集団（PPS）による解析をあわせて実施する。有害事象やその他の観察項目については、安全性解析対象集団を対象とする。これらの解析対象集団の定義は次のとおりである。

14.2.2 最大の解析対象集団（FAS）

プロトコル治療の少なくとも一部が実施された登録症例の集団とする。ただし、本登録後に不適格であることが判明した症例および有効性解析に関する情報が皆無である症例は除く。

14.2.3 治験実施計画書に適合した対象集団（PPS）

治験実施計画書に定められた手順を遵守した登録症例の集団とする。

14.2.4 安全性解析対象集団

プロトコル治療の少なくとも一部が実施された登録症例の集団とする。

14.3 解析項目・方法

以下に統計解析の概要を示す。検定の有意水準は両側 0.05 とする。詳細につい

ては統計解析計画書に記載する。統計解析計画書は統計解析責任者がデータ固定までに作成する。

14.3.1　解析対象の概要

1）　被験者の内訳

登録症例数、適格症例数、プロトコル治療開始症例数、中止症例数を群別に算出し、その結果をフロー図で示す。プロトコル治療を開始しなかった症例及び本登録後に不適格であることが判明した症例については、それらの理由別に集計する。

2）　治験実施計画書からの重大な逸脱

治験実施計画書に記載された手順や方法と異なる治療・検査を実施し、有効性の評価に影響があると医学的に判断される場合を重大な逸脱と定義する。重大な逸脱については理由ごとに集計し、有効性の解析における取り扱いは統計解析計画書で定める。また、プロトコル治療中止の有無及びその理由について群別に集計する。

3）　被験者背景因子及びベースラインデータ

被験者背景因子及びベースラインデータについて、適切な要約統計量（連続変数の場合には、症例数、平均値、標準偏差、計数値の場合には、症例数、最小値、中央値、最大値、及びカテゴリー項目の場合にはカテゴリー別症例数）を群別に算出する。

4）　治療の遵守状況の測定

治療の遵守状況を群毎に集計する。

14.3.2　主要評価項目に関する解析

MMSE スコアの変化量（シリアル 7 課題のスコアを用いる）

各被験者のスクリーニング時の MMSE スコアと治療開始後第 24、48、72、96 週スコアの差分について、その平均値を被験薬群と対照薬群間で比較検定する。ただし、第 96 週における差分スコアの群間平均値の有意差をもって本治験の有効性の確認とする。

この確認のため、治療群、性別及び時期を固定効果、被験者識別コード×時期を変量効果とした混合モデル反復測定（mixed model repeated mesasures: MMRM）[23] を用いる。

14.3.3　副次評価項目に関する解析

① MCI から「あらゆる原因による認知症」への移行までの時間

それぞれの期間に関して群別に Kaplan-Meier 法を適用し、生存関数を推定する。また、それらの生存率曲線を図示する。さらに、ログランク検定を適用し、それぞれの生存期間を群間比較する。さらに、被験者背景因子を含めた多変量 Cox 比例ハザードモデルを用いた解析を行う。

② 神経心理検査のスコア（CDR、ADAS-Cog 14、WMS-R Logical Memory）の変化量

各神経心理検査のスコアについて、MMSE と同様に解析する。CDR についてはCDR-SB を算出し解析する。

③ ADCS-MCI-ADL の変化量

ADL を 18 項目（0－53 点）で評価し、MMSE と同様に解析する。

④ MRI にて測定される海馬容積の変化量

FreeSurfer を用いて、自動セグメンテーションの手法で海馬領域の自動ラベリングを行い、左右の海馬容積を加算した総海馬容積を算出する。その上で、ベースライン時および第 96 週後の総海馬容積に基づき年間萎縮率を算出し、海馬萎縮の進行の程度とする。この値を被験薬群と対照薬群間で比較検定する。

14.3.4　安全性の解析

報告された有害事象は、MedDRA 辞書を用いて LLT コードを割り当てる。無作為化以降に発現した全ての有害事象は、器官別大分類（SOC）、基本語（PT）ごとに発生症例数を基に頻度を集計する。

有害事象は、報告された有害事象名、器官別大分類、基本語、無作為化からの発現までの期間、消失日、重篤度、程度、因果関係、転帰を含んだ一覧表を作成する。

14.4　中間解析

ベースライン集計を含むすべての中間集計及び中間解析は実施しない。

14.5　探索的解析

探索的解析として以下のシロスタゾールの IADL、神経心理検査（TMT・FCSRT）、

及び血清アルブミン-Aβ複合体濃度の解析を行う。

① IADL：ADCS-MCI-ADL（課題 19－24）の変化量

手段的日常生活動作（IADL）を課題 19～24 の 6 項目（0－16 点）で評価し、MMSE と同様に解析する。

② 神経心理検査：TMT Part A および B スコアの変化量

TMT Part A、Part B 検査が実施され、それぞれに要した時間（秒）が記録される（小数第 1 位以下は四捨五入とする）。この値を用いて MMSE と同様に解析する。

③ 神経心理検査：FCSRT スコアの変化量

FCSRT の各試行の自由再生（0－16 点）、全再生（0－16 点）、3 試行の自由再生の合計（0－48 点）及び全再生の合計（0－48 点）をそれぞれ評価し、各項目を MMSE と同様に解析する。

また、手掛かり効率を（全再生－自由再生）／（48－自由再生）と定義し、上記と同様に解析する。

④ 血清アルブミン-Aβ複合体濃度の変化量

被験者の血清アルブミン-Aβ複合体の濃度を、抗アルブミン、抗 Aβ抗体を用いたサンドイッチエライザ法を用いて調べる。この値を用いて MMSE と同様に解析する。

15 治験実施計画書、GCP 及び適用される規制要件の遵守

15.1 GCP 等の遵守

本治験は、世界医師会ヘルシンキ宣言（1964 年）及びその改訂版に基づく倫理的原則、医薬品、医療機器等の品質、有効性及び安全性の確保等に関する法律第 14 条第 3 項及び第 80 条の 2 に規定する基準、「医薬品の臨床試験の実施の基準に関する省令」（平成 9 年 3 月 27 日 厚生省令第 28 号）」（＝GCP［Good Clinical Practice の略］）及びその改正省令並びに運用通知、標準業務手順書並びに本治験実施計画書を遵守して実施する。

15.2 治験実施計画書の遵守

治験責任医師は、治験実施計画書、説明文書・同意文書について、実施医療機関の長に提出し、文書による治験実施の承認を得る。

治験責任医師等は治験実施計画書を遵守して治験を実施する。

15.3 治験実施計画書の逸脱又は変更

治験責任医師は、次の場合を除き治験審査委員会の事前の審査に基づく文書による承認を得ることなく、治験実施計画書からの逸脱又は変更を行ってはならない。

① 被験者の緊急の危険を回避する等、医療上やむを得ない場合
② 治験の事務的事項のみに関する変更である場合

上記①の場合、治験責任医師は、逸脱又は変更の内容及び理由並びに治験実施計画書の改訂が適切な場合にはその案を、可能な限り早急に実施医療機関の長及び治験審査委員会に提出して承認を得なければならない。

治験責任医師は、治験の実施に重大な影響を与え、又は被験者の危険を増大させるような治験のあらゆる変更について、実施医療機関の長及び治験審査委員会に速やかに文書にて報告する。

治験責任医師は、治験実施計画書から逸脱した行為の全てを記録した記録を保存する。

15.4 治験実施計画書の改訂

治験責任医師が治験実施計画書の改訂を必要と判断した場合、または治験責任医師が治験審査委員会あるいは効果安全性評価委員会に審議を依頼した結果改訂を提言された場合には、治験責任医師は治験実施計画書の改訂を行い、治験審査委員会・実施医療機関の長に改訂内容及びその理由を報告し、承認を得なければならない。ただし、有効性に関する評価項目の種類及び評価基準に関しては治験開始後、変更は認められない。

改訂手順については「治験実施計画書の作成及び改訂に関する手順書」に従う。

16 治験の終了又は中止

16.1 治験の終了

すべての被験者において、本治験実施計画書で規定された観察・検査・調査が終了したのち、治験責任医師は、実施医療機関の長に治験が終了した旨及び治験結果の概要を文書で報告する。

実施医療機関の長は治験審査委員会に対して、本治験の終了を速やかに文書で通知するとともに、治験責任医師から提出された報告書に基づき、本治験結果の概要を報告する。

16.2 治験の中止

16.2.1 治験の中止基準

治験責任医師は、以下の場合に本治験を中止する。

1) 本治験薬の品質、有効性又は安全性に関する事項などにより、治験調整医師及び治験責任医師が本治験の継続を困難と判断した場合。
2) 1)の情報に基づき、実施医療機関の長が中止を指示した場合。
3) 1)の情報に基づき、効果安全性評価委員会により本治験の中止が提言された場合。

16.2.2 治験の中止決定の手順

治験責任医師は、16.2.1 で定めた中止基準に該当する事項が発現した場合について、直ちに治験の中止等につき協議する。

治験の中止が決定された場合、治験調整医師は、直ちにすべての治験責任医師に報告するとともに、効果安全性評価委員会並びに治験薬提供者に報告する。

治験責任医師は速やかに被験者に中止及びその理由を伝え、被験者の安全性を確保する。

治験責任医師は実施医療機関の長、治験審査委員会及び関連部門にその旨を文書で報告し、実施医療機関に定められた手続きに従う。

17 各種委員会

17.1 効果安全性評価委員会

治験調整委員会は、治験の継続の適否又は治験実施計画書の改訂について審議する効果安全性評価委員会を設置する。効果安全性評価委員会の設置および実施については、「効果安全性評価委員会に関する手順書」に従う。

17.2 心理検査中央判定委員会

治験調整委員会は、正確な心理検査の判定のために MMSE、CDR 等の評価判定を行う心理検査中央判定委員会を設置する。心理検査中央判定委員会の設置および実施については、「心理検査中央判定委員会に関する手順書」に従う。

17.3 画像診断中央判定委員会

治験調整委員会は、正確な画像診断の判定のために総海馬容積の計測を行う画像診断中央判定委員会を設置する。画像診断中央判定委員会の設置および実施に

ついては、「画像診断中央判定委員会に関する手順書」に従う。

18 症例報告書

18.1 症例報告書の作成

治験責任医師又は治験分担医師は「電子症例報告書作成の手引き」に従って症例報告書を作成する。

症例報告書の作成、変更及び修正は治験責任医師又は治験分担医師が行う。ただし、医学的判断を伴わない事項については、治験責任医師又は治験分担医師の監督のもと、治験協力者が記入あるいはEDC（Electrical Data Capturingの略、電子データ収集システム）の場合は入力及び訂正してもよい。治験分担医師又は治験協力者が作成した症例報告書については、治験責任医師が記載内容に問題がないことを確認する。

18.2 症例報告書作成上の注意

治験責任医師又は治験分担医師は、症例報告書をすみやかに作成する。

症例報告書の変更又は修正は「電子症例報告書作成の手引き」に従う。

治験責任医師は、治験分担医師又は治験協力者が行った症例報告書の変更又は修正についても点検し、問題がないことを確認する。

症例報告書の記入内容が原資料との間に何らかの矛盾がある場合には、治験責任医師はその理由を説明する記録を作成し、保存する。

内容についての照会に際しては速やかに回答し、修正等が必要な場合には対応する。

19 治験の品質管理及び品質保証

19.1 品質管理

19.1.1 直接閲覧の対象となる原資料等の特定

本治験において規定する「データの根拠となる原資料」並びに「症例報告書に直接記入されることによりその記入以前に文書又は電子的に記録されたデータが存在しないデータ（症例報告書の記入内容が原資料となるデータ）」を以下に示す。

1） データの根拠となる原資料

- 被験者の同意及び被験者への情報提供に関する記録（被験者署名済同意書、診療情報提供書等）

- 診療録、カルテシールあるいはワークシート（本治験用に作成され診療録に添付された書類）、看護記録、症例登録に関する文書、本治験で規定された評価・検査・観察記録（検査データ、フィルム、検査伝票）等、症例報告書作成のもととなった記録
- 本治験で規定された治療に関する記録（治験薬管理表、処方記録等）。

2） 症例報告書の記載内容が原資料となるデータ

- 選択基準・除外基準の判定
- 既往歴・合併症
- 併用薬の使用理由
- 有害事象の重篤性・重症度・転帰・因果関係、コメント
- 本治験の中止理由、転帰に関するコメント
- 治験責任医師又は治験分担医師のコメント

19.1.2 直接閲覧

治験責任医師は、治験実施計画書又は別の合意文書中に、治験責任医師／実施医療機関が、治験に関連するモニタリング、監査、治験審査委員会による審査及び当局の査察の際に、原データ／原資料を直接閲覧に供すべき旨が記載されていることを保証するものとする。

モニタリング責任者及び担当者は、本治験が GCP、治験実施計画書、標準業務手順書及び関連する法規等に従って安全かつ適切に実施されていること、並びにデータの信頼性が十分に確保されていることを確認するために、原資料等の治験関連記録と症例報告書の照合を含む直接閲覧を行う。

実施医療機関の長及び治験責任医師は、モニタリング責任者及び担当者による施設訪問モニタリングを受け入れ、原資料等の全ての治験関連記録を直接閲覧に供しなければならない。

モニタリング責任者及び担当者は、原資料と症例報告書に何らかの矛盾がある場合には、その理由を説明した記録を治験責任医師より入手する。

19.1.3 モニタリング

モニタリング責任者及び担当者は、本治験実施について GCP を遵守し、本治験実施計画書、標準業務手順書及び関連する法規等に従い適切に行われていること、及びデータの信頼性が十分に確保されていることを確認するために、当該治験に係る「モニタリング手順書」に従って、施設訪問モニタリングを実施する。

19.1.4　データマネジメント

データマネジメント責任者及び担当者は、治験に関連するすべてのデータの信頼性とその適正な処理を保証するために、標準業務手順書に基づいてデータの取扱いの各段階において品質管理を実施する。

データマネジメント責任者及び担当者は、症例報告書の回収、データの点検・入力・変更・修正などのデータ固定までの手順について、当該治験に係る「データマネジメント計画書」等に基づき実施する。

19.2　品質保証

19.2.1　監査

監査責任者及び担当者は、本治験の実施、データ作成、文書化（記録化）及び報告が、GCP、治験実施計画書、標準業務手順書及び関連する法規等を遵守して実施されていることを、通常のモニタリング及び治験の品質管理業務とは独立・分離して評価する。

監査責任者及び担当者は、当該治験に係る「監査計画書」及び「監査手順書」に従い、品質保証活動の一環として、第三者の立場から監査を実施する。

19.2.2　原資料の直接閲覧の保証

実施医療機関の長及び治験責任医師は、モニタリング、監査ならびに規制当局及び治験審査委員会による調査の際に、原資料等すべての治験関連記録を直接閲覧に供し、これに協力するものとする。

20 記録の保存

20.1　実施医療機関

実施医療機関の長は、GCP で定められた実施医療機関にて保存すべき資料を保存する。

資料の保存期間は以下の①又は②のうちどちらか遅い日までとする。ただし、治験責任医師がこれよりも長期間の保存を必要とする場合には、保存期間及び保存方法について協議する。

①　治験薬提供者が被験薬に係る医薬品についての製造販売の承認を受ける日（開発中止もしくは本治験の成績が承認申請書に添付されない旨の通知を受けた日から 3 年が経過した日）

②　本治験の中止又は終了後 3 年が経過した日

治験責任医師は当該資料について、保存の必要がなくなった場合には、その旨を実施医療機関の長に通知する。

20.2　治験審査委員会

実施医療機関の長及び治験審査委員会の設置者は、GCP で定められた治験審査委員会で保存すべき資料を保存する。

資料の保存期間は以下の①又は②のうちどちらか遅い日までとする。ただし、治験責任医師がこれよりも長期間の保存を必要とする場合には、保存期間及び保存方法について協議する。

① 治験薬提供者が被験薬に係る医薬品についての製造販売の承認を受ける日（開発中止もしくは本治験の成績が承認申請書に添付されない旨の通知を受けた日から 3 年が経過した日）
② 本治験の中止又は終了後 3 年が経過した日

治験責任医師は当該資料について、保存の必要がなくなった場合には、その旨を当該治験審査委員会の設置者に通知する。

20.3　治験責任医師

治験責任医師は、GCP で定められたもののうち、治験責任医師が保存すべき資料を保存する。資料の保存場所及び期間については当該治験に係る「記録の保存に関する手順書」に従うものとする。

21 治験実施体制及び各種委員会

別紙 1「治験責任医師、各種委員会及び治験実施体制」を参照のこと。

22 治験実施上の倫理的配慮

22.1　治験審査委員会

22.1.1　治験実施の審査

本治験は、治験を実施することの適否について、倫理的、科学的及び医学的・薬学的観点から治験審査委員会の審査を受ける。

22.1.2　継続審査

1） 治験責任医師は、治験審査委員会の継続審査を受けるために、本治験の

現況の概要を年 1 回又は治験審査委員会の求めに応じてそれ以上の頻度で、実施医療機関の長に文書にて報告する。

2) 実施医療機関の長は、必要に応じて、実施医療機関における本治験継続の適否について治験審査委員会の意見を聴くことができる。

22.2 被験者の個人情報保護に関する事項

本治験の関係者は、関係法規に従って被験者の個人情報及びプライバシー保護に十分配慮する。

被験者の登録及び症例報告書における被験者の特定は、被験者識別コードで行う。本治験の実施に係る原データ類及び被験者の同意文書等の直接閲覧並びに本治験成績の公表においては、被験者の氏名、疾患等のプライバシー及び個人情報保護に十分配慮する。なお、作成された症例報告書は本治験の目的以外には使用しない。また、本治験で知り得た情報は、一切、第三者に漏洩しない。

ただし、各被験者の検体や画像データ等の試料は、今後の研究のための貴重な試料として本治験終了後も国立循環器病研究センター病院が保管する。なお、この治験とは異なる研究等で当該試料を用いる場合は、改めて研究計画を倫理委員会等にて承認を得る必要がある。

23 治験の費用負担及び補償

23.1 資金源及び利益相反

本治験は治験調整医師及び／又は治験責任医師が大塚製薬株式会社からの助成をうけ、国立循環器病研究センター病院が実施医療機関と共同で行う。

なお、被験薬および対照薬は大塚製薬株式会社から提供される。

本治験で起こりうる利益相反について、治験責任医師及び治験分担医師は実施医療機関に対して自己申告を行い、実施医療機関内の規定に従い対応する。

23.2 治験に関する費用

本治験では、保険外併用療養費制度が適用されるため、治験薬及び治験薬と同様の効能又は効果を有する医薬品(同種同効薬)に係る投薬／使用に係る費用以外は、保険外併用療養費制度の下に保険診療として実施され、被験者の健康保険が適用される。なお、現時点においてＡβ排泄を促進させる効能が期待されているホスホジエステラーゼⅢ阻害薬であるシロスタゾールの同種同効薬は上市されていない。

23.3　健康被害に対する補償

本治験は、被験者に健康被害が生じた場合、実施医療機関はその治療に関する医療の提供など必要かつ適切な処置を行う。その際、当該健康被害が治験薬の適正使用により生じ、治験薬との因果関係があると治験責任医師が判断した場合、治験責任医師は補償費用を負担する。ただし、医療費及び医療手当は支払われない。なお、当該健康被害が被験者の故意又は重大な過失によるものと判断した場合は、補償の対象外となる。

本治験に起因する健康被害による賠償責任が生じた場合の履行措置として、治験調整委員会は治験調整医師、治験責任医師、治験分担医師及び実施医療機関が被保険者となる治験保険に加入する。また、治験責任医師及び治験分担医師は医師賠償責任保険に加入し、実施医療機関は病院賠償責任保険等に加入する。

24 治験の登録、成果の帰属と公表

24.1　臨床試験登録

本治験は Clinicaltrials.gov へ臨床試験登録する。

24.2　成果の帰属と公表

本治験の成果は、治験調整医師に帰属するものとする。本治験の結果を医薬品／医療機器製造承認申請等の目的で使用する権利については、必要に応じて協議し、別途定めることとする。

結果の公表にあたっては、治験調整医師と本治験に参加する全ての治験責任医師が協議の上、筆頭著者を決定し、英語論文として発表する。ただし、治験調整医師、このほか協議によって決められたものの連名による共同発表とし、連絡先は治験調整医師とする。

25 参考文献

1.「認知症疾患治療ガイドライン」作成合同委員会編. 認知症疾患治療ガイドライン 2010. 東京. 医学書院. 2010. p.188-189.

2. 朝田 厚生労働科学研究費補助金（認知症対策総合研究事業）. 総合研究報告書（平成 23 年度～平成 24 年度）. 都市部における認知症有病率と認知症の生活機能障害への対応. 研究代表者. 朝田 隆. 2013.

3. Ott A, Breteler MM, van Harskamp F, Stijnen T, Hofman A. Incidence and risk of dementia. The Rotterdam Study. Am J Epidemiol. 1998; 147: 574-580.

4. Matsui Y, Tanizaki Y, Arima H, Yonemoto K, Doi Y, Ninomiya T, Sasaki K, Iida M, Iwaki T, Kanba S, Kiyohara Y. Incidence and survival of dementia in a general population of Japanese elderly: the Hisayama study. J Neurol Neurosurg Psychiatry. 2009; 80: 366-370.

5. Weller RO, Subash M, Preston SD, Mazanti I, Carare RO. Perivascular drainage of amyloid-beta peptides from the brain and its failure in cerebral amyloid angiopathy and Alzheimer's disease. Brain Pathol 2008; 18: 253–266.

6. Yamada M, Ihara M, Okamoto Y, Maki T, Washida K, Kitamura A, Hase Y, Ito H, Takao K, Miyakawa T, Kalaria RN, Tomimoto H, Takahashi R. The influence of chronic cerebral hypoperfusion on cognitive function and amyloid β metabolism in APP overexpressing mice. PLoS ONE 2011; 6: e16567.

7. Okamoto Y, Yamamoto T, Kalaria RN, Senzaki H, Maki T, Hase Y, Kitamura A, Washida K, Yamada M, Ito H, Tomimoto H, Takahashi R, Ihara M. Cerebral hypoperfusion induces microinfarcts in cerebral amyloid angiopathy. Acta Neuropathol 2012; 123: 381-394.

8. Maki T, Okamoto Y, Carare R, Hase Y, Hattori Y, Hawkes C, Saito S, Yamamoto Y, Terasaki Y, Ishibashi-Ueda, H, Taguchi A, Takahashi R, Miyakawa T, Kalaria R, Lo E, Arai K, Ihara M. Phosphodiesterase III inhibitor promotes drainage of cerebrovascular β-amyloid. Ann Clin Transl Neurol; in press

9. Snowdon DA, Kemper SJ, Mortimer JA, Greiner LH, Wekstein DR, Markesbery WR. Linguistic ability in early life and cognitive function and Alzheimer's disease in late life: findings from the Nun Study. JAMA 1996; 275: 528-532.

10. Arai H, Takahashi T. A combination therapy of donepezil and cilostazol for patients with moderate Alzheimer disease: pilot follow-up study. Am J Geriatr Psychiatry 2009; 17: 353-354.

11. Taguchi A, Takata Y, Ihara M, Kasahara Y, Tsuji M, Nishino M, Stern D, Okada M. Cilostazol improves cognitive function in patients with mild cognitive impairment: a retrospective analysis. Psychogeriatrics. 2013; 13: 164-169.

12. Ihara M, Nishino M, Taguchi A, Yamamoto Y, Hattori Y, Saito S, Takahashi Y, Tsuji M, Kasahara Y, Takata Y, Odaka M. Cilostazol add-on therapy in patients with mild dementia receiving donepezil: a retrospective study. PLoS ONE. 9: e89516.
13. Albert MS, Dekosky ST, Dickson D, Dubois B, Feldman HH, Fox NC, Gamst A, Holtzman DM, Jagust WJ, Petersen RC, Snyder PJ, Carrillo MC, Thies B, Phelps CH. The diagnosis of mild cognitive impairment due to Alzheimer's disease: Recommendations from the National Institute on Aging-Alzheimer's Association workgroups on diagnostic guidelines for Alzheimer's disease. Alzheimers Dement. 2011; 7: 270-279.
14. Petersen RC, Stevens JC, Ganguli M, Tangalos EG, Cummings JL, Dekosky ST. Practice parameter: early detection of dementia: mild cognitive impairment (an evidence-based review). Report of the Quality Standards Subcommittee of the American Academy of Neurology. Neurology. 2001; 56: 1133-1142.
15. Petersen RC, Doody R, Kurz A, Mohs RC, Morris JC, Rabins PV, Ritchie K, Rossor M, Thal L, Winblad B. Current concepts in mild cognitive impairment. Arch Neurol. 2001; 58: 1985-1992.
16. Thompson PD, Zimet R, Forbes WP, Zhang P. Meta-Analysis of Results from Eight Randomized, Placebo-Controlled Trials on the Effect of Cilostazol on Patients With Intermittent Claudication. Am J Cardiol. 2002; 90: 1314–1319
17. Kwon SU, Hong KS, Kang DW, Park JM, Lee JH. et al. Efficacy and safety of combination antiplatelet therapies in patients with symptomatic intracranial atherosclerotic stenosis. Stroke. 2011; 42(10):2883-90.
18. Uchiyama S, Sakai N, Toi S, Ezura M, Okada Y, Takagi M, Nagai Y, Matsubara Y, Minematsu K, Suzuki N, Tanahashi N, Taki W, Nagata I, Matsumoto M; CATHARSIS Study Group. Final Results of Cilostazol-Aspirin Therapy against Recurrent Stroke with Intracranial Artery Stenosis (CATHARSIS). Cerebrovasc Dis Extra. 2015; 5(1):1-13.
19. Mckhann GM, Knopman DS, Chertkow H, Hyman BT, Jack CR Jr, Kawas CH, Klunk WE, Koroshetz WJ, Manly JJ, Mayeux R, Mohs RC, Morris JC, Rossor MN, Scheltens P, Carrillo MC, Thies B, Weintraub S, Phelps CH. The diagnosis of dementia due to Alzheimer's disease: Recommendations from the National Institute on Aging-Alzheimer's Association workgroups on diagnostic guidelines for Alzheimer's disease. Alzheimers Dement. 2011; 7: 263-269.
20. McKhann G, Drachman D, Folstein M, Katzman R, Price D, Stadlan EM. Clinical diagnosis of Alzheimer's disease: report of the NINCDS-ADRDA Work Group under the auspices of Department of Health and Human Services Task Force on Alzheimer's

Disease. Neurology. 1984; 34: 939-944.

21. ADNI Alzheimer's Disease Neuroimaging Initiative. [cited 2014 Jun; Available from: http://adni.loni.ucla.edu]
22. Julious SA. Sample Size for Clinical Trials. Boca Raton. CRC Press. 2010.
23. Pinheiro JC, Bates DM. Mixed-Effects Models in S and S-Plus, Springer, 2001.

Confidential

軽度認知障害患者に対するシロスタゾール療法の臨床効果ならびに安全性に関する医師主導治験

COMCID (TRINEU1321)

Statistical Analysis Plan
（統計解析計画書）
1.0 版

日付：2020 年 4 月 28 日

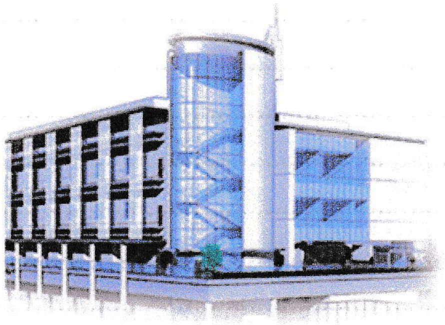

統計解析責任者:竹綱 正典
2020/4/28 竹綱正典

公益財団法人 神戸医療産業都市推進機構
医療イノベーション推進センター

Translational Research Center for Medical Innovation
founded in 2003 by MEXT & Kobe City
for acceleration of translational research in Japan

Copyright © 2020 Translational Research Center for Medical Innovation, Foundation for Biomedical Research and Innovation at Kobe All Rights Reserved.

目次

1　解析に関わる基本的事項 5
1.1　研究の目的及び Research Question 5
1.2　シェーマ 5
1.3　研究の概要 6
1.4　データの保管場所 8
1.5　解析ソフトウェア 8
2　解析目的 9
2.1　治験実施計画書からの解析方法の変更 9
3　解析対象集団 9
3.1　安全性解析対象集団（SS: Safety Set） 9
3.2　最大の解析対象集団（FAS: Full Analysis Set） 9
3.3　プロトコル遵守集団（PPS: Per Protocol Set） 9
3.4　重大なプロトコル逸脱 9
4　評価項目の定義 11
4.1　有効性評価項目 11
4.1.1　主要評価項目 11
4.1.2　副次評価項目 11
4.1.3　探索的評価項目 11
4.2　安全性評価項目 11
4.3　その他の評価項目 12
4.3.1　層別項目 12
5　データハンドリング 13
5.1　前（ベースライン）の定義 13
5.2　週・月・年換算 13
5.3　タイムウインドウ 13
5.4　臨床検査値の検出限界の取扱い 13
6　解析方法 14
6.1　症例構成 14
6.2　解析対象例 14
6.3　患者背景 14
6.4　治療状況 14
6.5　有効性評価項目 14
6.5.1　主要評価項目 15
6.5.2　副次評価項目（解析対象：FAS） 15

Copyright © 2020 Translational Research Center for Medical Innovation, Foundation for Biomedical Research and Innovation at Kobe All Rights Reserved.

6.5.3 探索的評価項目 16
6.6 安全性評価 16
6.6.1 有害事象 16
6.6.2 臨床検査値 17
6.7 目標登録症例数の設定根拠 17
7 付録 17
8 参照資料 17
8.1 治験実施計画書 第 1.6 版（2018 年 7 月 2 日作成） 17
9 引用文献 17
10 改訂履歴 17
Copyright © 2020 Translational Research Center for Medical Innovation, Foundation for Biomedical Research and Innovation at Kobe All Rights Reserved.

略語一覧

略語	語形	
Aβ	Amyloid Beta	βアミロイド
AD	Alzheimer’s Disease	アルツハイマー型認知症
ADAS-Cog 14	Alzheimer's Disease Assessment Scale-Cognitive 14	アルツハイマー病評定尺度-認知行動 14
ADAS-Cog-J 14	Alzheimer's Disease Assessment Scale-Cognitive 14 - Japanese	アルツハイマー病評定尺度-認知行動 14 日本語版
ADCS-MCI-ADL	Alzheimer's Disease Cooperative Study-Mild Cognitive Impairment – Activities of Daily Living Inventory	アルツハイマー病共同研究 - MCI - 日常生活動作質問票
ADL	Activities of Daily Living	日常生活動作
ADS	Analysis Data Set	解析データセット
AE	Adverse Event	有害事象
BMI	Body Mass Index	肥満指数
CDR	Clinical Dementia Rating	臨床認知症評価法
CDR-J	Clinical Dementia Rationg-Japanese	臨床認知症評価法 - 日本版
CDR-SB	Clinical Dementia Rating - Sum of Boxes	臨床認知症評価法 - 合計点法 -
DM	Data Manager	データマネージャー
FAS	Full Analysis Set	最大の解析対象集団
GCP	Good Clinical Practice	医薬品の臨床試験の実施の基準に関する省令
IADL	Instrumental Activities of Daily Living	手段的日常生活動作
LLT	Low Level Term	下層語
LSMean	Least Square Mean	調整平均
MCI	Mild Cognitive Impairment	軽度認知障害
MedDRA/J	Medical Dictionary of Regulatory Activities/J	医薬品規制用語集日本語版
MMRM	Mixed effect model for Repeated Mesures	混合効果モデルによる反復測定データ解析法
MMSE	Mini - Mental State Examination	精神状態短時間検査
MMSE-J	Mini - Mental State Examination – Japanese	精神状態短時間検査-日本版
MRI	Magnetic Resonance Imaging	核磁気共鳴画像検査
N	Number of patients	患者数
Possible AD Dementia	Possible Alzheimer’s Disease Dementia	アルツハイマー型認知症の疑診
Probable AD Dementia	Probable Alzheimer’s Disease Dementia	アルツハイマー型認知症の確診
PPS	Per Protocol Set	プロトコル遵守集団
PT	Preffered Term	基本語
Q1	1st quartile	第 1 四分位点(25%点)
Q3	3rd quartile	第 3 四分位点(75%点)
SD	Standard Deviation	標準偏差
SE	Standard Error	標準誤差
SOC	System Organ Class	器官別大分類

Copyright © 2020 Translational Research Center for Medical Innovation, Foundation for Biomedical Research and Innovation at Kobe All Rights Reserved.

TRI	Translational Research Center for Medical Innovation	医療イノベーション推進センター
TMT	Trail Making Test	トレイルメイキングテスト
WMS-R	Wechsler Memory Scale-Reviced	ウエクスラー記憶検査法

Copyright © 2020 Translational Research Center for Medical Innovation, Foundation for Biomedical Research and Innovation at Kobe All Rights Reserved.

1　解析に関わる基本的事項

1.1 研究の目的及び Research Question

目的：軽度認知障害（MCI）患者に対するシロスタゾール投与の臨床効果と安全性について、ミニメンタルステート検査（MMSE）スコアを用いて評価する。

Research Question：

1) シロスタゾールはプラセボと比較して、MCI から認知症への移行を抑制する。
2) シロスタゾールはプラセボと比較して、認知障害の進行を抑制する
3) シロスタゾールはプラセボと比較して、MCI 患者の日常生活動作（ADL）の低下を抑制する
4) シロスタゾールはプラセボと比較して、海馬萎縮の進行を抑制する

1.2 シェーマ

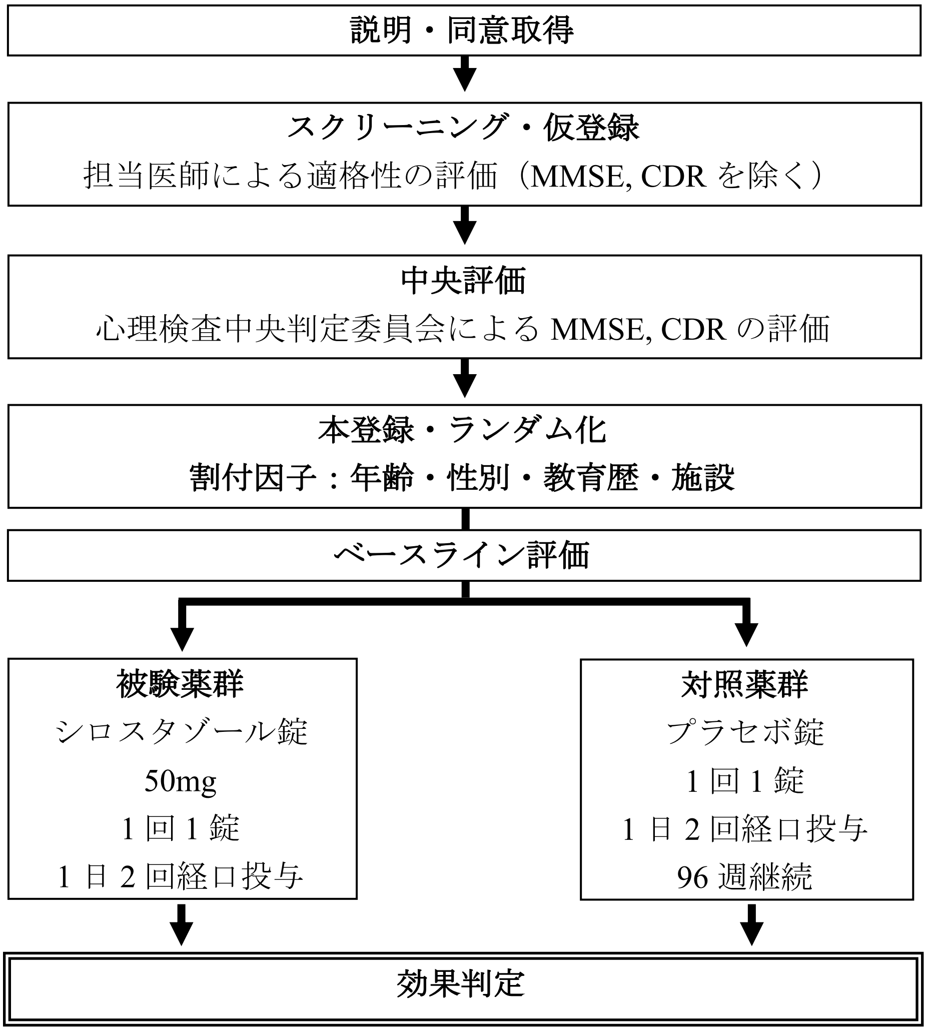

Copyright © 2020 Translational Research Center for Medical Innovation, Foundation for Biomedical Research and Innovation at Kobe All Rights Reserved.

1.3 研究の概要

目標症例数	200 例（被験薬群：100 例、対照薬群：100 例）
症例登録期間	2015 年 5 月～2018 年 3 月
追跡終了日	最終症例登録の 2 年後（2020 年 3 月）
観察・介入	並行群間比較試験
対照の種類	同時並行プラセボ対照
ランダム化	年齢（75 歳未満 vs75 歳以上）、性別、教育歴（12 年以下 vs.12 年超える）及び施設を割付調整因子とする最小化法
盲検化のレベル	二重盲検

Copyright © 2020 Translational Research Center for Medical Innovation, Foundation for Biomedical Research and Innovation at Kobe All Rights Reserved.

観察スケジュール

検査・観察・評価項目 ＼ 時期	同意取得	スクリーニング		導入期(ベースライン)		治療期：治療開始後第 4 週以内	治療期：治療開始後第 24 週	治療期：治療開始後第 48 週	治療期：治療開始後第 72 週	治療期：治療開始後第 96 週	中止時
同意取得	●										
無作為化				●							
基本情報<br>病歴聴取		●		●							
神経所見		●		●		●	●	●	●	●	●
臨床検査 A			仮登録	●							
臨床検査 B			心理検査<br>中央評価		治療<br>開始	●					●
臨床検査 C							●				
血清アルブミン-Aβ複合体濃度測定			本登録	●			●				
画像検査（脳 MRI）注 1				●						●	●
神経心理検査：MMSE		●					●	●	●	●	●
神経心理検査：CDR		●						●		●	●
神経心理検査：ADAS, WMS-R, TMT, FCSRT 注 1				●				●		●	●
ADCS-MCI-ADL				●				●		●	●
併用薬の服薬状況の確認				←	─	─	─	─	─	─	→
治験薬の服薬状況の確認						←	─	─	─	─	→
有害事象					←	─	─	─	─	─	→
転帰調査						←	─	─	─	─	→
許容範囲			仮登録後 4 週以内に本登録<br>本登録後 4 週以内に治療開始				前後<br>14 日以内	前後<br>14 日以内	前後<br>14 日以内	前後<br>14 日以内	中止後<br>28 日以内

注 1：一部の症例において実施.

Copyright © 2020 Translational Research Center for Medical Innovation, Foundation for Biomedical Research and Innovation at Kobe All Rights Reserved.

1.4 データの保管場所

DM から提供されたデータ及び解析に用いた解析データセット (ADS) を以下に保存する。

DM Dataset:	Z:¥01_プロジェクト¥TRI プロジェクト¥197_TRINEU1321(猪原)¥09_生物統計¥09_最終解析¥2_ANALYSIS¥EXECUTE1¥01_DATA¥DM_RAW
ADS:	Z:¥01_プロジェクト¥TRI プロジェクト¥197_TRINEU1321(猪原)¥09_生物統計¥09_最終解析¥2_ANALYSIS¥EXECUTE1¥01_DATA¥ADS

1.5 解析ソフトウェア

解析には SAS Version 9.4（SAS Institute Inc., Cary, NC, USA.）及び R version 3.4.1 （2017-06-30）を用いる。

Copyright © 2020 Translational Research Center for Medical Innovation, Foundation for Biomedical Research and Innovation at Kobe All Rights Reserved.

2 解析目的

本統計解析計画書は、治験実施計画書に定められた最終解析の詳細を定めるものである。

2.1 治験実施計画書からの解析方法の変更

治験実施計画書第 1.6 版（2018 年 7 月 2 日作成）からの解析方法の変更を以下に示す。

変更後の内容	変更理由
6.5.1 主要評価項目験の解析<br>各被験者の治療開始後第 24、48、72、96 週のスコアの差分（治療開始後―スクリーニング時）について、スクリーニング時の MMSE スコアを共変量とし、治療群、時点、性別、時点と治療群の交互作用を母数効果、被験者を変量効果とした反復測定混合効果モデル（MMRM: mixed-effects model with repeated measurements）をあてはめ、第 96 週における差分の群間差（被験薬群－対照群）の最小二乗平均(LSMean)の有意性をもって、本治験の有効性を確認する。	治験実施計画書では、第 96 週の差分スコアの群間平均値の有意差で有効性を確認すると記載があるが、MMRM による最小二乗平均(LSmean)の信頼区間で評価することを明記した。

3 解析対象集団

3.1 安全性解析対象集団（SS: Safety Set）

プロトコル治療が行われた全ての症例のうち、以下の症例を除いた集団を安全性解析の解析対象集団（安全性解析対象集団）とする。

- 文書同意なしの症例

3.2 最大の解析対象集団（FAS: Full Analysis Set）

安全性解析対象集団のうち、以下の症例を除いた最大の解析対象集団を有効性解析対象集団とする。

- 登録後に不適格症例であることが判明した症例
- 登録時に有効性に関わるデータがない症例
- プロトコル治療後のいずれの時点にも有効性に関わるデータがない症例

3.3 プロトコル遵守集団（PPS: Per Protocol Set）

FAS のうち、重大なプロトコル逸脱のない症例をプロトコル遵守集団として、有効性の感度解析の対象集団とする。

3.4 重大なプロトコル逸脱

重大なプロトコル逸脱と症例の取扱いは、原則として以下のとおりとする。なお、データマネジメント担当者は症例の集積にあわせてプロトコルの逸脱を評価し、試験データの固

Copyright © 2020 Translational Research Center for Medical Innovation, Foundation for Biomedical Research and Innovation at Kobe All Rights Reserved.

定までに開催される症例検討会で重大なプロトコル逸脱と症例の取扱いを定める。

重大なプロトコル逸脱

			取扱い	重大な逸脱
登録基準違反				
	選択基準違反	年齢＜55 歳、年齢≧85 歳	採用	×
		有効性に関わる選択基準に違反した症例	FAS 除外	○
	除外基準違反	有効性に関わる除外基準に違反した症例	FAS 除外	○
		安全性に関わる除外基準に違反した症例	PPS 除外	○
GCP 違反				
	文書同意違反	文書同意なしの症例	SS 除外	○
		同意日以前にプロトコル治療が開始された症例	SS 除外	○
		同意日以前に仮登録された症例	SS 除外	○
治療不遵守				
	治療不遵守	一定期間の服薬率が不明もしくは 60 %未満の症例	PPS 除外	○
併用薬違反				
	併用禁止薬の処方例	併用禁止薬が投与された症例	PPS 除外	○
観察不遵守				
	来院違反	プロトコルで規定された許容範囲（前後 14 日以内）に観察が実施できなかった症例	採用	×
中止基準違反				
	中止基準違反	プロトコルで規定された中止基準に従ってプロトコル治療を中止しなかた症例	採用	×

Copyright © 2020 Translational Research Center for Medical Innovation, Foundation for Biomedical Research and Innovation at Kobe All Rights Reserved.

4　評価項目の定義

4.1 有効性評価項目

4.1.1 主要評価項目

MMSE（邦訳としてMMSE-Jを使用）スコアの変化量（スクリーニング時と治療開始後第24, 48, 72, 96週測定時の差分）

4.1.2 副次評価項目

4.1.2.1 MCI から「あらゆる原因による認知症」への移行までの時間

割付日から最初に認知症と診断された日までの日数（認知症診断日 - 割付日＋１）と定義する。

4.1.2.2 CDR-SB の変化量

4.1.2.3 ADAS-Cog14（邦訳として ADAS-Cog-J 14 を使用）の変化量。

4.1.2.4 WMS-R Logical Memory（日本版ウエクスラー記憶検査法 論理的記憶Ⅰと論理的記憶Ⅱを使用）の変化量

4.1.2.5 ADCS-MCI-ADL を用いた ADL（18 項目：0-53 点）の変化量

4.1.2.6 MRI で測定される海馬容積の変化量

4.1.3 探索的評価項目

4.1.3.1 IADL：ADCS-MCI-ADL（課題 19－24）の変化量

4.1.3.2 神経心理検査：TMT Part A および B スコアの変化量

4.1.3.3 神経心理検査：FCRST スコアの変化量

4.1.3.4 血清アルブミン-Aβ複合体濃度の変化量

4.2 安全性評価項目

報告された有害事象は、MedDRA/J 辞書を用いて LLT コードを割り当てる。解析にはMedDRA/J の最新バージョンを用いることとする。
治験薬投与開始日から治験薬投与終了日の翌日までに認められた有害事象を集計する。介入との因果関係が「関連あり」と判定された有害事象を副作用とする。

有害事象発生までの期間に関する起点日は治験薬投与開始日とする。
有害事象発生までの期間 ＝ 有害事象発現日 － 治験薬投与開始日 ＋ 1

Copyright © 2020 Translational Research Center for Medical Innovation, Foundation for Biomedical Research and Innovation at Kobe All Rights Reserved.

一例中に同一 PT の有害事象が複数回発現した場合は、発現した回数を件数とし、例数は 1 例として集計する。重症度は判定された程度のうち最も重い程度をその症例の重症度として集計する。因果関係が一度でも有と判定された場合、症例の因果判定を有として集計する。例えば、以下の例では「頭痛」の発現件数 3 件、発現例数 1 例、重症の「頭痛」1 例、及び副作用としての「頭痛」1 例と集計する。

症例番号	LLT	PT	発現日	程度	因果関係
100-01	頭痛	頭痛	2014/8/1	重症	有
	頭重感	頭痛	2014/9/1	中等症	無
	頭痛	頭痛	2014/10/1	中等症	無

4.3 その他の評価項目

4.3.1 層別項目

MCIの病型分類：健忘型MCI（Amnestic MCI）と非健忘型MCIを層別項目とする。
またそれぞれの型別に、単一領域と複数領域についても層別項目とする。

Copyright © 2020 Translational Research Center for Medical Innovation, Foundation for Biomedical Research and Innovation at Kobe All Rights Reserved.

5　データハンドリング

5.1 前（ベースライン）の定義

ベースラインは、導入期（ベースライン値判定期間）のデータとする。ただし、導入期（ベースライン値判定期間）のデータが欠測値の場合はスクリーニング期のデータをベースラインとする。

5.2 週・月・年換算

日数より週・月・年換算は以下の式による。

週換算：日数 / 7

月換算：日数 ×（12/365.25）

年換算：日数 /365.25

5.3 タイムウインドウ

有効性評価項目の解析における観察期の時点（中止日も含める）は、治療開始日から次の Time Window により Visit 時点を算出する Calculated Visit とする。

ただし観察日数は、

　観察日数＝測定日 － 治療期開始日 ＋1

とする。

また、同一 Time Window 内に 2 つの観察値が認められる場合、目標日数に近いものをその時点の観察値とする。

		解析上の範囲		プロトコル上の許容範囲	
	目標	下限	上限	下限	上限
4 週	28	1	28	1	28
24 週	168	29	252	154	182
48 週	336	253	420	322	350
72 週	504	421	588	490	518
96 週	672	589	730	658	686

5.4 臨床検査値の検出限界の取扱い

臨床検査値の要約統計量においては、臨床検査値の検出限界とされた検査値は、施設の限界値を測定値として扱う。ただし、一覧表では欠測とし、定量限界値未満であることがわかるよう表示する。

Copyright © 2020 Translational Research Center for Medical Innovation, Foundation for Biomedical Research and Innovation at Kobe All Rights Reserved.

6 解析方法

- 症例数は整数で表示し、割合（%）並びに発生頻度（%）及びその信頼区間の表示桁数は小数点以下 1 桁とする。発生頻度（%）の信頼区間は Clopper-Pearson 法による正確な信頼区間を算出する。
- 要約統計量の算出において、平均値、標準偏差（SD）、中央値の小数点は 1 桁増しで表示する。同様に、調整平均（LSMean）及び標準誤差（SE）の小数点も 1 桁増しで表示する。最小値及び最大値の小数点は同じとする。
- 検定での有意水準は両側 5%とする。多重検定の問題への対応は考慮しない。
- 信頼区間の算出は全て両側 95%信頼区間とする。
- P 値については、0.001 未満の場合は「p<0.001」と表示する。0.001 以上の場合は小数点以下 4 桁を四捨五入し、表示桁は小数点以下 3 桁とする。

6.1 症例構成

仮登録症例数、本登録症例数、プロトコル治療開始例数、中止症例数を群別に算出する。プロトコル治療を開始しなかった症例及び本登録後に不適格であることが判明した症例については、それらの理由別に集計する。

6.2 解析対象例

SS、FAS、PPS ごとにその症例数を表記する。除外症例は除外理由別に集計し、除外症例一覧表を作成する。

6.3 患者背景

FAS の患者背景（年齢、BMI、病歴等）について、定量変数は要約統計量（症例数、平均値、標準偏差、最小値、中央値、及び最大値）、定性変数は症例数・割合（%）を投与群ごとに求め、群間の偏りを 2 標本 t 検定、Fisher の直接確率法、及び Wilcoxon 順位和検定により検討する。背景項目のカテゴリー区分は図表見本に示す。
既往歴・合併症は MedDRA/J 辞書を用いて、器官別大分類（SOC：System Organ Class）、基本語（PT：Preferred Term）ごとに頻度を集計する。
前治療・併用薬は医薬品名データファイル（2016/04）コードを割り当て、薬効分類（医薬品コード 3 桁、4 桁）、医薬品名（医薬品コード 7 桁）ごとに頻度を集計する。
薬物治療でない前治療・併用治療は併用治療ごとに頻度を集計する。

6.4 治療状況

各群の治験薬の服薬状況を継時的に集計する。

6.5 有効性評価項目

FAS での解析を有効性に関する主要な解析とし、主要評価項目に付いては FAS の解析に

Copyright © 2020 Translational Research Center for Medical Innovation, Foundation for Biomedical Research and Innovation at Kobe All Rights Reserved.

加えて、PPS による解析を補足的解析として実施する。

6.5.1 主要評価項目

各被験者の治療開始後第 24、48、72、96 週のスコアの差分（治療開始後 - スクリーニング時）について、スクリーニング時の MMSE スコアを共変量とし、治療群、時点、性別、時点と治療群の交互作用を母数効果、被験者を変量効果とした反復測定混合効果モデル（MMRM: mixed-effects model with repeated measurements）をあてはめ、第 96 週における差分の群間差（被験薬群－対照群）の最小二乗平均(LSMean)の有意性をもって、本治験の有効性を確認する。
なお、被験者内の分散共分散構造は unstructured（UN）を仮定する。収束しない場合は、下記の構造を順に仮定し、修正 AIC（Corrected AIC：AICc）が最小となる分散共分散構造を採用し、最適モデルとする。

- Heterogeneous Toeplitz（TOEPH）
- Heterogeneous AR(1)（ARH(1)）
- Heterogeneous CS（CSH）
- Toeplitz（TOEP）
- Compound Symmetry（CS）

さらに、各時点の調整平均を縦軸に、時点を横軸に経時推移グラフを作成する。なお、途中中止例は中止時点のデータを、最も日数が近い観測時点のデータとみなして、解析に用いる。また、測定時点毎の要約統計量（例数、平均値、標準偏差、最大値、Q3、中央値、Q1、最小値）を示す。

6.5.2 副次評価項目（解析対象：FAS）

6.5.2.1 MCI から「あらゆる原因による認知症」への移行までの時間

群別に Kaplan-Meier 法を適用して生存関数を推定し、それらの推定生存曲線を図示する。またログランク検定を用いて群間比較を行い、Cox 比例ハザードモデルを用いてハザード比の推定を行う。さらに背景因子に偏りがあった場合は、背景因子を共変量とした多変量 Cox 比例ハザードモデルによる解析も行う。

6.5.2.2 CDR-SB

各被験者のスクリーニング時の CDR と治療開始後第 48、96 週の CDR の差分について MMSE と同様の解析を行う。

6.5.2.3 ADAS-Cog 14

各被験者のベースラインの ADAS-Cog 14 と治療開始後第 48、96 週との差分について MMSE と同様の解析を行う。

Copyright © 2020 Translational Research Center for Medical Innovation, Foundation for Biomedical Research and Innovation at Kobe All Rights Reserved.

6.5.2.4 WMS-R Logical Memory
各被験者のベースラインの WMS-R Logical Memory と治療開始後第 48、96 週との差分について MMSE と同様の解析を行う。

6.5.2.5 ADCS-MCI-ADL
各被験者のベースラインの ADCS-MCI-ADL（18 項目：0-53 点）と治療開始後第 48、96 週との差分について MMSE と同様の解析を行う。

6.5.2.6 MRI で測定される海馬容積の変化量
各被験者のベースラインの海馬容積と治療開始後 96 週（もしくは中止時）との差分についてベースラインの海馬容積を共変量とした共分散分析を用いて群間比較を行う。

6.5.3 探索的評価項目

6.5.3.1 IADL
各被験者のベースラインの ADCS-MCI-ADL（課題 19-24）と治療開始後第 48、96 週との差分について MMSE と同様の解析を行う。

6.5.3.2 神経心理検査：TMT Part A および B スコアの変化量
各被験者のベースラインの ADCS-MCI-ADL（課題 19-24）と治療開始後第 48、96 週との差分について MMSE と同様の解析を行う。

6.5.3.3 神経心理検査：FCSRT スコアの変化量
各被験者のベースラインの FCSRT スコアと治療開始後第 48、96 週との差分について MMSE と同様の解析を行う。

6.5.3.4 血清アルブミン-Aβ複合体濃度の変化量
各被験者の治療開始後第 24 週の血清アルブミン-Aβ複合体濃度のベースラインからの差分についいベースラインの値を共変量とする共分散分析を用いて群間比較を行う。さらにすべてのデータを用いて、CDR-SB と血清アルブミン-Aβ複合体濃度の散布図を作成し、相関係数を算出する。

6.6 安全性評価

6.6.1 有害事象
有害事象について以下の解析を行う。

- 群ごとの発現例数と発生割合を集計し有害事象に係る事象の要約をする。有害事象、副作用、重篤な有害事象及び中止に結び付いた有害事象について発生割合の 95%信頼区間を算出し、Fisher の直接確率検定を用いて群間比較を行う。
- 有害事象は、群ごと、SOC、PT ごとに発現例数と発生割合を集計する。
- 副作用は、群ごと、SOC、PT ごとに発現例数と発生割合を集計する。

Copyright © 2020 Translational Research Center for Medical Innovation, Foundation for Biomedical Research and Innovation at Kobe All Rights Reserved.

- 有害事象は、群ごと、SOC、PT、重症度別ごとに発現例数と発生割合を集計する。
- 副作用は、群ごと、SOC、PT、重症度別に発現例数と発生割合を集計する。
- 重篤な有害事象は、群ごと、SOC、PT ごとに発現例数と発生割合を集計する。
- 有害事象は、重篤・非重篤ごと、群ごと、SOC、PT ごとに発現例数と発生割合を集計する。
- 治験薬投与中止に至った有害事象は、群ごと、SOC、PT、因果関係別に発現例数と発生割合を集計する。
- 発生時期によらず報告された全ての死亡一覧表、重篤な有害事象一覧表、治験薬投与中止に至った有害事象一覧表を作成する。

6.6.2 臨床検査値

臨床検査値は、前と後と前後差の要約統計量（症例数、平均値、標準偏差、最小値、中央値、及び最大値）を求める。また、症例毎、検査項目、時期毎に一覧表を作成する。

6.7 目標登録症例数の設定根拠

目標症例数：200 例（被験薬群 100 例、対照薬群 100 例）

【設定の根拠】

標本数は各群 100 例とする。この症例数は目標達成の可能性から定めた。しかしながら、この標本数は Type I エラー5%、検出力 90%及び標準偏差 3.7 の条件下で、本登録時から治療開始後第 96 週の MMSE スコア変化量の差分について両群間でその平均値を検定した場合、1.72 の群間差を検出できるものである。この標準偏差は米国 ADNI 研究グループが公開しているデータ及び田口らの研究を参照した。また、この標本数設定の方法は Julious に従った。

7 付録

8 参照資料

8.1 治験実施計画書　第 1.6 版（2018 年 7 月 2 日作成）

9 引用文献

プロトコル参照

10 改訂履歴

版	作成日	主な内容	作成者
1.0*	2019/2/18	*(TRI 初版)作成	竹綱正典
1.0	2020/4/28	1.0 版作成	竹綱正典

Copyright © 2020 Translational Research Center for Medical Innovation, Foundation for Biomedical Research and Innovation at Kobe All Rights Reserved.

医療イノベーション推進センター（TRI）による事前の承諾なくして、本資料の全て又は一部を譲渡、複製、公表、その他の無断転載することは禁止されています。

発行
〒650-0047 神戸市中央区港島南町 1-5-4
医療イノベーション推進センター
TEL: 078-303-9107　FAX: 078-303-9094
URL: http://www.tri-kobe.org

Copyright © 2020 Translational Research Center for Medical Innovation, Foundation for Biomedical Research and Innovation at Kobe All Rights Reserved.
Template 2018.4.25
