# Supplementary material for: Efficacy and Safety of Cilostazol in Mild Cognitive Impairment: A Randomized Clinical Trial
Source: JAMA Netw Open. 2023 Dec 4;6(12):e2344938. doi: 10.1001/jamanetworkopen.2023.44938 (PMC10696485; doi:10.1001/jamanetworkopen.2023.44938)
Supplement: Supplement 2. — eTable 1. Eligibility Criteria eTable 2. Drug Adherence Rate eTable 3. Change in MMSE Scores From Baseline in Stratified Patients eFigure 1. Schedule of Efficacy Assessments eFigure 2. Effects of Cilostazol on Psychological Tests eMethods. eReferences [file jamanetwopen-e2344938-s002.pdf]

Supplemental Online Content

Saito S, Suzuki K, Ohtani R, et al. Efficacy and safety of cilostazol in mild cognitive impairment: a randomized clinical trial. *JAMA Netw Open*. 2023;6(12):e2344938. doi:10.1001/jamanetworkopen.2023.44938

eTable 1. Eligibility Criteria
eTable 2. Drug Adherence Rate
eTable 3. Change in MMSE Scores From Baseline in Stratified Patients
eFigure 1. Schedule of Efficacy Assessments
eFigure 2. Effects of Cilostazol on Psychological Tests
eMethods
eReferences

This supplemental material has been provided by the authors to give readers additional information about their work.

© 2023 Saito S et al. *JAMA Network Open*.

eTable 1. Eligibility Criteria

**Inclusion criteria**
• Clinical diagnosis of MCI according to the core clinical criteria of the NIA/AA classification<br>• Age between 55 and 84 years<br>• MMSE scores ≥22 and ≤28 points<br>• CDR score of 0.5 points<br>• Written informed consent of two participants: a patient and a study partner who was sufficiently familiar with the daily life of the patient
**Exclusion criteria**
• Parkinson's disease, Huntington's disease, normal pressure hydrocephalus, progressive supranuclear palsy, epilepsy, multiple sclerosis, cerebral infection, or head trauma with sequelae<br>• Multiple cerebral infarctions, brain tumor, or subdural hematoma<br>• Major depression or bipolar disorder<br>• Poorly controlled diabetes mellitus (HbA1c >9.0%)<br>• Hypoglycemic episode with loss of consciousness<br>• Cognitive impairment caused by vitamin B_{12} or folate deficiency or thyroid function abnormality<br>• Neurosyphilis<br>• Previous history of alcohol abuse<br>• Bleeding disorders, congestive heart failure, or coronary artery stenosis<br>• Severe hypertension at the time of registration<br>• Contraindication for MRI scans<br>• Use of insulin, psychoactive drugs, anti-dementia drugs, cilostazol, anticoagulants, or more than two types of antiplatelet drugs<br>• Hypersensitivity to cilostazol<br>• Participation in any other drug trials for AD<br>• Pregnancy or breast feeding at the time of registration<br>• Difficulty in neuropsychological tests<br>• Any other reasons that would prevent patients from participating in the trial

© 2023 Saito S et al. *JAMA Network Open*.

Abbreviations: AD, Alzheimer's disease; CDR, Clinical Dementia Rating; HbA1c, glycated hemoglobin; MCI, mild cognitive impairment; MMSE, Mini-Mental State Examination; MRI, magnetic resonance imaging; NIA/AA, National Institute on Aging and the Alzheimer's Association

© 2023 Saito S et al. *JAMA Network Open*.

eTable 2. Drug Adherence Rate

	Placebo group			Cilostazol group		
	<50%	50–80%	≥80%	<50%	50–80%	≥80%
24-week visit	0 (0)	1 (1.3)	75 (98.7)	0 (0)	2 (2.9)	66 (95.7)
48-week visit	0 (0)	0 (0)	64 (98.5)	0 (0)	2 (3.3)	59 (96.7)
72-week visit	0 (0)	1 (1.7)	57 (98.3)	0 (0)	2 (3.8)	50 (96.2)
96-week visit	0 (0)	7 (12.7)	48 (87.3)	0 (0)	2 (4.2)	46 (95.8)

Data represent numbers (percentages).

© 2023 Saito S et al. *JAMA Network Open*.

eTable 3. Change in MMSE Score From Baseline in Stratified Patients

	<75 years old		≥75 years old	
	Placebo	Cilostazol	Placebo	Cilostazol
24-week visit	−0.3 (2.9)	−0.8 (2.1)	0.1 (2.2)	−0.5 (2.7)
48-week visit	−0.8 (3.0)	−0.7 (1.8)	−0.7 (2.8)	−1.2 (2.9)
72-week visit	−1.1 (2.8)	−0.9 (2.9)	−0.6 (3.1)	−1.1 (3.0)
96-week visit	−0.8 (2.6)	−1.1 (3.1)	−1.0 (2.0)	−1.7 (3.5)

	Male		Female	
	Placebo	Cilostazol	Placebo	Cilostazol
24-week visit	−0.3 (2.9)	0.1 (2.5)	0.1 (2.2)	−1.1 (2.4)
48-week visit	−0.5 (2.5)	−1.2 (2.7)	−0.8 (3.1)	−0.8 (2.4)
72-week visit	−0.7 (2.6)	−1.1 (2.7)	−0.9 (3.3)	−0.9 (3.2)
96-week visit	−1.0 (2.1)	−1.8 (3.3)	−0.9 (2.4)	−1.2 (3.3)

	≤12 years of education		>12 years of education	
	Placebo	Cilostazol	Placebo	Cilostazol
24-week visit	−0.1 (2.2)	−0.4 (2.6)	−0.1 (3.1)	−1.0 (2.1)
48-week visit	−0.5 (2.9)	−0.9 (2.6)	−1.0 (2.9)	−1.0 (2.4)
72-week visit	−0.8 (3.0)	−0.7 (2.8)	−0.9 (3.0)	−1.5 (3.3)
96-week visit	−1.0 (2.5)	−1.2 (3.1)	−0.7 (1.7)	−1.9 (3.6)

Data represent mean (standard deviation).

© 2023 Saito S et al. *JAMA Network Open*.

eFigure 1. Schedule of Efficacy Assessments

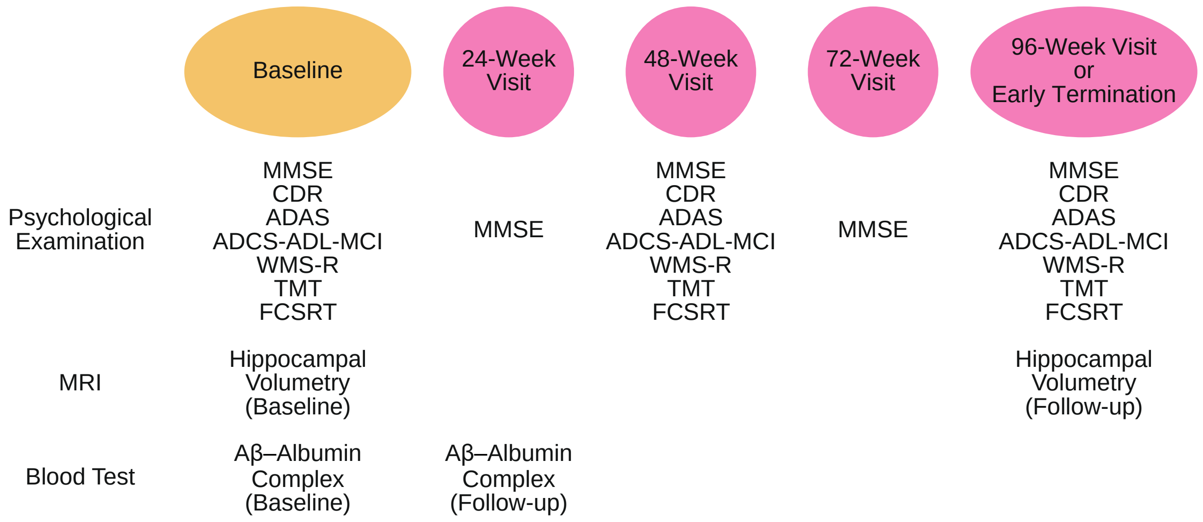

Abbreviations: Aβ, β-amyloid; ADAS, Alzheimer's Disease Assessment Scale; ADCS-ADL-MCI, Alzheimer's Disease Cooperative Study-Activities of Daily Living for Mild Cognitive Impairment; CDR, Clinical Dementia Rating; FCSRT, Free and Cued Selective Reminding Test; MRI, magnetic resonance imaging; MMSE, Mini-Mental State Examination; TMT, Trail Making Test; WMS-R, Wechsler Memory Scale-Revised

© 2023 Saito S et al. *JAMA Network Open*.

eFigure 2. Effects of Cilostazol on Psychological Tests

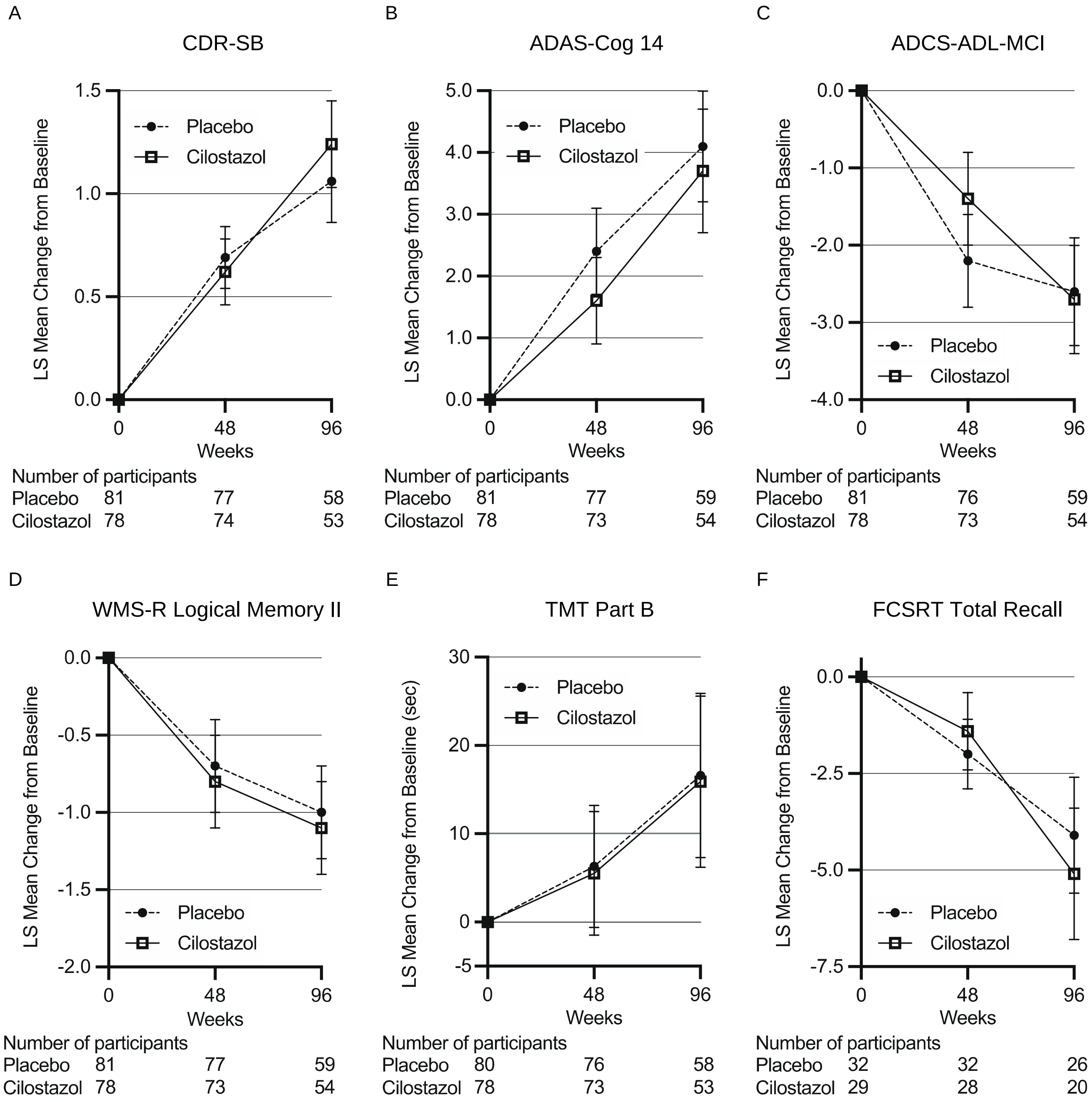

© 2023 Saito S et al. *JAMA Network Open*.

A. Least-squares mean change from baseline on the CDR-SB. No significant difference was observed between the two groups at the 48-week (*P*=.727) and 96-week (*P*=.531) visits.

B. Least-squares mean change from baseline on the ADAS-Cog 14. There was no significant difference between the two groups at the 48-week (*P*=.450) and 96-week (*P*=.749) visits.

C. Least-squares mean change from baseline in the ADCS-ADL-MCI. No difference was observed at the 48-week (*P*=.326) and 96-week (*P*=.904) visits.

D. Least-squares mean change from baseline in the WMS-R Logical Memory II. No difference between the groups was observed at the 48-week (*P*=.854) and 96-week (*P*=.753) visits.

E. Least-squares mean change from baseline on the TMT part B. No difference between groups was observed at the 48-week (*P*=0.934) or 96-week (*P*=.961) visits.

F. Least-squares mean change from baseline FCSRT score. No difference between the groups was observed at the 48-week (*P*=.659) and 96-week (*P*=.675) visits. A mixed model with repeated measures was applied by considering treatments, time points, sex, and interaction of treatments and time points as fixed effects, patients as a random effect, and baseline scores as a covariate in all analyses. The error bars indicate the standard errors.

Abbreviations: ADAS-Cog 14, 14-item Alzheimer's Disease Assessment Scale-Cognitive Subscale; ADCS-ADL-MCI, Alzheimer's Disease Cooperative Study-Activities of Daily Living for Mild Cognitive Impairment; CDR-SB, Clinical Dementia Rating Sum of Box; FCSRT, Free and Cued Selective Reminding Test; TMT, Trail Making Test; WMS-R, Wechsler Memory Scale-Revised

© 2023 Saito S et al. *JAMA Network Open*.

eMethods

Trial Procedures

In this Cilostazol for Prevention of Conversion from MCI to Dementia (COMCID) study, enrolled participants were randomly assigned to the cilostazol or placebo group using minimization methods. We adjusted the stratification factors, including age (≥75 years), sex, education (>12 years), and institution, as there were concerns on the potential distribution imbalance between the placebo and cilostazol groups, which could potentially affect the results, considering the current medium-sized randomized trial. The randomization list was kept by a vendor who was not involved in the data collection or analysis (CAC Croit Corporation, Tokyo, Japan). In this trial, no emergency unblinding was performed. A web-based electronic data capture system was used to collect clinical data from the patients' medical records. All procedures complied with the principles of the Declaration of Helsinki revised in 2013, International Council for Harmonization of Technical Requirements for Pharmaceuticals for Human Use Guidelines for Good Clinical Practice, and Japan's Pharmaceutical Affairs Law. Quality control was performed at each data-handling step by an external data manager (CMIC Co., Ltd., Tokyo, Japan). The process of data collection was monitored by a clinical research associate team (Linical Co., Ltd., Osaka, Japan). This trial has been registered with the Japanese regulatory agency.

Psychological Examinations

According to the standard operating procedure of psychological examinations, testing was performed by trained psychologists or medical doctors who completed the training programs and passed a qualifying examination. The central psychological review board,

© 2023 Saito S et al. *JAMA Network Open*.

rather than the site testers, scored the results based on the test records, which included answers from patients and their partners. The Mini-Mental State Examination (MMSE) is a 30-point questionnaire extensively used in clinical and research settings to measure cognitive impairment.[1] The Japanese version of the MMSE,[2] which has been validated and verified as reliable, was used. The Clinical Dementia Rating (CDR) is a global dementia staging instrument based on a semi-structured interview with the patient and caregiver, which can provide an index of global functioning.[3] The rater scores patients in each of six cognitive domains (memory, orientation, judgment and problem-solving, community affairs, home and hobbies, and personal care). The CDR Sum of Box score is the total score for each domain.[4] The 14-item Alzheimer's Disease Assessment Scale-Cognitive Subscale (ADAS-Cog 14) was used to assess multiple cognitive domains, including memory, language, praxis, and orientation.[5] The Wechsler Memory Scale-Revised (WMS-R) Logical Memory II is designed to measure verbal episodic memory.[6] Only Story A was used in the current study. The Alzheimer's Disease Cooperative Study-Activities of Daily Living for Mild Cognitive Impairment (ADCS-ADL-MCI) is a functional evaluation scale for patients with MCI based on the information provided by a caregiver.[7] The Free and Cued Selective Reminding test (FCSRT)[8] is a battery test that evaluates episodic memory and is performed only at the National Cerebral and Cardiovascular Center. Low scores on the MMSE, WMS-R Logical Memory II, ADCS-ADL-MCI, and FCSRT indicate a more severe condition, whereas a high score on the CDR, CDR Sum of Box, and ADAS-Cog 14 indicates impaired cognitive function. The Trail Making Test (TMT) assesses processing speed and executive function.[9] The results of the TMT Part B were reported. A longer time indicates impaired function.

© 2023 Saito S et al. *JAMA Network Open*.

Hippocampal Volumetry

Hippocampal volume was measured at baseline, at the 96-week visit, or at early termination. The participants were evaluated using a 3.0-Tesla magnetic resonance imaging (MRI) scanner manufactured by Siemens (National Cerebral and Cardiovascular Center, Verio; National Center for Geriatrics and Gerontology, TIM Trio; Kyoto University and Kyoto Prefectural University, Skyra; AG, Germany). T1-weighted images were collected using a three-dimensional sagittal magnetization-prepared rapid gradient echo sequence, according to the Alzheimer's disease Neuroimaging Initiative 3.0 Tesla MRI protocol.[10] Automated hippocampal volumetry was performed using the FreeSurfer software version 5.3 (Laboratory for Computational Neuroimaging at the Athinoula A. Martinos Center for Biomedical Imaging, Charlestown, MA, USA)[11] by the central evaluation committee, who were blinded to the clinical information.

β-Amyloid–Albumin Complexes

The blood levels of Aβ–albumin complexes were measured by a sandwich enzyme-linked immunosorbent assay (ELISA) using an anti-human Aβ N-terminal monoclonal antibody (BAN50) and an anti-human albumin purified polyclonal antibody, as previously reported.[12] Briefly, serum samples were diluted to 1:400 with Tris-buffered saline before loading. A 100-μL aliquot from each diluted sample was loaded into a microwell strip coated with BAN50, contained within a commercial kit (Human β-Amyloid$_{1-42}$ ELISA Kit; Wako Pure Chemical Industries, Osaka, Japan). The plates were sealed and incubated for 60 min at 37 °C before being washed four times with 0.05%

© 2023 Saito S et al. *JAMA Network Open*.

Tween 20 in Tris-buffered saline. After washing, 100 μL of purified horseradish peroxidase-conjugated anti-human albumin polyclonal antibody (A80-129P, Bethyl Laboratories, Montgomery, TX, USA) diluted to 1:30,000 with 10% BlockAce (Snow Brand, Tokyo, Japan) was added to the microwell strips, which were subsequently incubated for 60 min at 37 °C. After subsequent washing, 100 μL of the 3, 3', 5, 5'-tetramethylbenzidine substrate (Wako Pure Chemical Industries) was added, and the strips were further incubated for 15 min at 37 °C. Acid solution (100 μL; Wako Pure Chemical Industries) was subsequently added to each well to terminate the enzyme reaction and stabilize the developed color. The optical density of each well was subsequently measured at 450 nm using an iMark microplate reader (Bio-Rad Laboratories, Inc., Hercules, CA, USA). The assay was performed in duplicate, and the results were averaged. The intra- and inter-assay coefficients of variability were 5.1% and 13.8%, respectively. To create a standard curve for ELISA, we sequentially diluted the human serum albumin preparation used for intravenous injection (25 mg/mL, Benesis Corporation, Osaka, Japan). The sandwich ELISA successfully detected Aβ–albumin complexes in the albumin preparation, which decreased in a linear manner in the serially diluted standard. The standard curves in the present study were linear, with an r value of 0.96–0.99. The Aβ–albumin complex levels are expressed as the albumin preparation equivalent concentration from a standard curve based on reference standards. Concentrations of Aβ–albumin complexes below the lower detection limit (2.5 mg/mL) were set to zero.

Evaluation of Adverse Events

Safety analyses included all adverse events (AEs) in participants allocated to the

© 2023 Saito S et al. *JAMA Network Open*.

cilostazol or placebo groups. Serious AEs were described as events that resulted in death, were life-threatening, required in-patient hospitalization or prolongation of current hospitalization, or resulted in persistent or significant disability. Their severity was determined based on whether the events interfered with the daily activities of the participants. The causal relationship of AEs to the treatment protocol was determined as “yes,” “possible,” or “no.” These classifications were evaluated by the COMCID coordinating committee and site physician. Investigators at other sites verified the evaluation of serious adverse events. Furthermore, an independent data monitoring committee discussed the severe AEs and whether the study protocol should be revised.

© 2023 Saito S et al. *JAMA Network Open*.

eReferences

1. Folstein MF, Folstein SE, McHugh PR. "Mini-mental state". A practical method for grading the cognitive state of patients for the clinician. *J Psychiatr Res.* 1975;12(3):189-198.

2. Sugishita M, Koshizuka Y, Sudou S, et al. The Validity and Reliability of the Japanese Version of the Mini-Mental State Examination (MMSE-J) with the original procedure of the Attention and Calculation Task (2001). Article in Japanese. *Ninchi Shinkei Kagaku.* 2018;20(2):91-110.

3. Morris JC. The Clinical Dementia Rating (CDR): current version and scoring rules. *Neurology.* 1993;43(11):2412-2414.

4. Berg L, Miller JP, Baty J, Rubin EH, Morris JC, Figiel G. Mild senile dementia of the Alzheimer type. 4. Evaluation of intervention. *Ann Neurol.* 1992;31(3):242-249.

5. Rosen WG, Mohs RC, Davis KL. A new rating scale for Alzheimer's disease. *Am J Psychiatry.* 1984;141(11):1356-1364.

6. Kawano N, Awata S, Ijuin M, Iwamoto K, Ozaki N. Necessity of normative data on the Japanese version of the Wechsler Memory Scale-Revised Logical Memory subtest for old-old people. *Geriatr Gerontol Int.* 2013;13(3):726-30.

7. Galasko D, Bennett D, Sano M, et al. An inventory to assess activities of daily living for clinical trials in Alzheimer's disease. The Alzheimer's Disease Cooperative Study. *Alzheimer Dis Assoc Disord.* 1997;11 Suppl 2:S33-39.

8. Grober E, Buschke H, Crystal H, Bang S, Dresner R. Screening for dementia by memory testing. *Neurology.* 1988;38(6):900-903.

9. Reitan RM. Validity of the Trail Making Test as an indicator of organic brain

© 2023 Saito S et al. *JAMA Network Open*.

damage. *Perceptual and Motor Skills.* 1958;8(3):271-276.

10. Alzheimer’s Disease Neuroimaging Initiative. Methods & Tools: MRI ACQUISITION. Accessed 3.16, 2023. https://adni.loni.usc.edu/methods/mri-tool/mri-analysis/. Accessed March 16, 2023.

11. Fischl B, Salat DH, Busa E, et al. Whole brain segmentation: automated labeling of neuroanatomical structures in the human brain. *Neuron*. 2002;33(3):341-355.

12. Yamamoto K, Shimada H, Koh H, Ataka S, Miki T. Serum levels of albumin-amyloid beta complexes are decreased in Alzheimer's disease. *Geriatr Gerontol Int.* 2014;14(3):716-723.

© 2023 Saito S et al. *JAMA Network Open*.
