# Supplementary material for: Efficacy and Safety of Cilostazol in Mild Cognitive Impairment: A Randomized Clinical Trial
Source: JAMA Netw Open. 2023 Dec 4;6(12):e2344938. doi: 10.1001/jamanetworkopen.2023.44938 (PMC10696485; doi:10.1001/jamanetworkopen.2023.44938)
Supplement: Supplement 3. — Nonauthor Collaborators. Nonauthor members of the COMCID Trial Investigator Group [file jamanetwopen-e2344938-s003.pdf]

Supplemental Online Content: Nonauthor Collaborators
*First name, last name, and suffix (if applicable) are required and will appear in PubMed.

***Group Name(s): Nonauthor Members of the COMCID Trial Investigator Group**							
***First Name and Middle Initial(s)**	***Last Name**	***Suffix (eg, Jr, III)**	Academic Degrees	Institution	Location (city, state/province, country)	Role or Contribution, eg, chair, principal investigator	Group (if more than 1 Group listed in the byline) and/or Subgroup (eg, Steering Committee)
Masahiro	Tsuji		MD, PhD	Department of Food and Nutrition, Kyoto Women's University	Kyoto, Kyoto, Japan	Chair	The independent data and safety monitoring board
Atsushi	Ouchi			Department of Neurology, National Cerebral and Cardiovascular Center	Suita, Osaka, Japan	Board member	The central psychological review board
Miho	Yamauchi			Department of Neurology, National Cerebral and Cardiovascular Center	Suita, Osaka, Japan	Board member	The central psychological review board
