# Supplementary material for: Efficacy and Safety of Cilostazol in Mild Cognitive Impairment: A Randomized Clinical Trial
Source: JAMA Netw Open. 2023 Dec 4;6(12):e2344938. doi: 10.1001/jamanetworkopen.2023.44938 (PMC10696485; doi:10.1001/jamanetworkopen.2023.44938)
Supplement: Supplement 4. — Data Sharing Statement [file jamanetwopen-e2344938-s004.pdf]

Data Sharing Statement

Saito. Efficacy and Safety of Cilostazol in Mild Cognitive Impairment. *JAMA Netw Open*. Published December 04, 2023. doi:10.1001/jamanetworkopen.2023.44938

Data
Data available: No
